# Correction: *Drakenstein Child Health Study (DCHS): investigating determinants of early child development and cognition*

**DOI:** 10.1136/bmjpo-2018-000282corr1

**Published:** 2018-07-12

Donald KA, Hoogenhout M, du Plooy CP, *et al*. Drakenstein Child Health Study (DCHS): investigating determinants of early child development and cognition. *BMJ Paediatrics Open* 2018;2:e000282. doi:10.1136/bmjpo-2018-000282.

In the fourth paragraph of the Discussion some rogue text was inadvertently inserted into the sentence ‘This is especially true of cohorts from table 1 LMICs where youLMICsildren youLMICsildren youLMICsildreniyouLMICsildrenidemicsand non-communicable diseases.’ The sentence should read: ‘This is especially true of cohorts from LMICs where young children are exposed to overlapping epidemics of infectious and non-communicable diseases.’ We have corrected this error and replaced with a revised version.

