# Supplementary material for: Drakenstein Child Health Study (DCHS): investigating determinants of early child development and cognition
Source: BMJ Paediatr Open. 2018 Jun 13;2(1):e000282. doi: 10.1136/bmjpo-2018-000282 (PMC6014194; doi:10.1136/bmjpo-2018-000282)
Supplement: Supplementary file 1 [file bmjpo-2018-000282supp001.docx]

**Supplementary Material**

**Protocol: The Drakenstein Child Health Study (DCHS)**

**Measures used in the DCHS**

These measures aimed to assess overall infant and child development and neurobiological health longitudinally. Broadly, the methods assessed (1) social and biological risk and protective factors, (2) general neurobiological development, (3) cognitive development, and (4) socio-emotional development.

Developmental risk and protective factors

*Antenatal risk factors.* The parent psychosocial measures are described elsewhere ^1^. These included parent reports of whether the birth was planned and partner support was available; maternal depression; alcohol, nicotine and illicit substance use; maternal childhood trauma; intimate partner violence and exposure to stressful events; psychological distress; and symptoms of peri- and posttraumatic stress. The parent psychosocial measures were completed by the mothers during several antenatal and postnatal study visits. Basic demographic data and health information for the infants were obtained from participants’ hospital records.

*Exposure to traumatic events and violence.* The Paediatric Emotional Distress Scale (PEDS) is a brief screening tool for the occurrence of traumatic events during childhood, and is completed by the parent ^2^. The PEDs asks about the child’s previous experience of a traumatic event and trauma-related experience of anxiety/withdrawal, fearfulness and acting out/ externalising behaviours. Higher scores indicate greater emotional distress.

The Child Exposure to Community Violence Checklist (CECV) is a 33-item parent-report checklist that evaluates children’s exposure to violence. The questions contain information on the type and level of violence that the child has witnessed or personally experienced (as victim or perpetrator). Higher scores indicate greater exposure to violence. It has been developed and validated in the South African context. Each of the violence categories have shown good internal consistency in this context ^3^. These questionnaires are included because of the strong body of literature on the detrimental effects of childhood exposure to violence on developmental outcomes ^4-6^.

The Survey for Exposure to Community Violence (SECV) is a 12-item checklist that assesses maternal exposure to community violence ^7, 8^. As with the CECV, higher scores indicate greater exposure to violence.

*Parenting.* Parenting is measured through a combination of dyadic interaction observations and maternal self-report of parenting practices and attachment. Dyadic interaction is assessed using the Global Rating Scale (GRS) ^9^ at 14 weeks and the Emotional Availability Scale (EAS), fourth edition ^10^, at 3.5 and 4.5 years*.* Recordings of unstructured maternal-child interactions were done at all three times points. The interactions were recorded during scheduled well-baby visits to Paarl hospital. In the GRS, mothers were instructed to play with their child for 5 minutes without the use of toys. The recordings were scored by raters certified on the GRS, who assessed maternal style (13 items), child temperament (7 items), and the nature of the dyadic interaction (5 items). Infant behaviour items assess attentiveness, active communicative attempts and engagement with the environment. Maternal items assess the mother’s responsiveness and sensitivity, directiveness and affect ^9, 11^. Interaction items assess the fluidity and emotional valence of the interaction ^12^.

In the EAS, videotaped unstructured play interactions of 10 minutes in length were coded by trained raters. The EAS includes four parental scales and two child scales; that is, maternal sensitivity, structuring, non-intrusiveness and non-hostility (parental scales), as well as child responsiveness and child involvement ^10, 13^. Each scale consists of two subscales rated on a 7-point scale and five subscales rated on a 3-point scale. Higher values signify more desirable behaviour. The EAS has sound psychometric properties internationally and has been used to assess emotional availability from infancy to middle childhood in different social and cultural settings, including South Africa ^14, 15^. Previous studies report good inter-rater reliability ^15, 16^.

The Parenting and Family Adjustment Scale (PAFAS) is a 30-item parent-report measure for assessing parenting practices, parent-child relationship quality, parental emotional adjustment and teamwork, and family relationships ^17^. The inventory consists of two scales; parenting and family adjustment. Higher scores indicate greater levels of dysfunction. The PAFAS subscales have shown good internal consistency and adequate construct validity in previous research ^17^.

*Maternal attachment* was measured using the self-report Brockington Postpartum Bonding Questionnaire ^18^. The questionnaire screens for impaired bonding, rejection, anger, anxiety and incipient abuse. High scores indicate more pathological responses. The questionnaire has shown good test-retest reliability ^18^ as well as high sensitivity for identifying bonding disorders ^19^, and has been used in low-SES South African settings ^20^.

*Child resilience* was measured using the Child and Youth Resilience Measure (CYRM-28) ^21^. This 26-item parent-report questionnaire measures child resilience, with higher scores indicating greater resilience. The questionnaire focusses on seven aspects of resilience; namely, access to material resources, supportive relationships, identity, sense of social justice, feelings of power and control, cohesion, and cultural adherence. It was collaboratively developed in several low-and middle-income countries to enable cross-cultural comparison of developmental outcomes and has satisfactory internal consistency in this context ^22^.

Neurobiological development

*Brain development.* A subgroup of children in the Drakenstein Child Health Study cohort underwent multimodal neuroimaging assessment, including those at risk of exposure to alcohol, maternal depression and HIV in pregnancy. The imaging was done at the Cape Universities Brain Imaging Centre (CUBIC) using a Siemens 3 Tesla Skyra machine. The imaging modalities performed included: (1) structural magnetic resonance imaging (MRI) with T1 and T2-weighting to examine cortical and subcortical volumes; (2) diffusion tensor imaging (DTI) for white matter microstructure; (3) magnetic resonance spectroscopy (MRS) and; (4) resting state functional MRI for regional connectivity. The imaging was undertaken at 2-4 weeks of age and again at 2.5-3 years during natural sleep without sedation. Due to the logistical challenges of imaging children at this age, the imaging was conducted at a time of day when the children usually sleep. The imaging has no radiation exposure. The imaging aims to detect anatomical and functional differences associated with antenatal and childhood risk factors.

*Western Cape Developmental Screening Questionnaires* were administered to the children’s caregivers at 6 weeks, 9 months and 18 months of age by primary health care workers during routine immunization visits to the clinic. Each age-specific screening questionnaire (6 weeks, 9 months or 18 months) contains a set of yes/no questions that assess developmental milestones in the domains of hearing, vision, gross motor, fine motor, language and communication, and psychosocial development. The screening questionnaires also assesses caregiver mental health and caregiver-child interaction. The questionnaires form part of the Western Cape Government Department of Health’s primary health care screening for moderate and severe neurodevelopmental disabilities and provides a categorical risk outcome score (risk / no risk).

*Bayley Scales of Infant and Toddler Development, Third Edition* (Bayley-III) ^23^. The Bayley-III is a clinician-administered instrument designed to assess developmental functioning between the ages of 1 and 42 months. The instrument monitors children’s developmental progress in the areas of cognition, motor skills and language, as well as socio-emotional and adaptive functioning ^24^. Each subscale is scored according to age-appropriate norms. A cut-off of 7 or less in any subscale scaled score is a difference of 1 standard deviation from the mean and indicates suboptimal development in that domain ^23^. The Bayley-III was administered on a subset of infants at ages 6 months and the whole cohort at 2 years in the DCHS in order to assess childhood development over time. It has been used globally, including in LMIC settings such as South Africa ^25^.

**Cognitive development**

*General cognitive function.* A measure of general cognitive function was obtained through the Kaufman Assessment Battery for Children, second edition (KABC-II; Pearson Assessments). The KABC-II Mental Processing scale is utilised due to the diverse cultural and language demographics within the Drakenstein communities. The KABC-II was primarily designed to be a culture-fair tool used to assess cognitive function in children aged 3.0 – 18.11 years old, minority groups and children with learning disabilities ^26^. In specific, the following subtests of the KABC-II were utilised:

1. Conceptual Thinking (problem solving)
2. Face Recognition (visual-spatial processing)
3. Triangles (visual-spatial processing and problem solving)
4. Hand Movements (working memory and motor sequencing)

The Mental Processing scale of the KABC-II is reported to have excellent internal and test-retest reliability, as well as good convergent validity ^27^. The KABC-II has been used in a variety of settings in Africa and South Africa ^28-32^.

*Language.* The Peabody Picture Vocabulary Test, fourth edition (PPVT-IV) ^33^ and KABC-II Expressive Vocabulary ^34^ subtests were used to assess language. The KABC-II Expressive Vocabulary task measures the ability to verbally identify a set of pictures. The PPVT-4 measures receptive vocabulary and is designed for use in a wide age range (ages 2.5 to 90 years). The examiner reads out a word, and then the child responds by pointing to the picture they think corresponds to the word the examiner has given. The test is untimed, but takes approximately 10 – 15 minutes. The PPVT-4 has excellent test-retest reliability and internal consistency ^33^. The PPVT-4 has been translated into both Afrikaans and isiXhosa ^35^.

*Memory.* The KABC-II Atlantis tasks ^36^ were used to assess learning and recall. The Atlantis task assesses the ability to learn and remember a series of nonsense words paired with pictures. The child is instructed to point to the correct picture when a word is read. The task is repeated after a 15 – 20-minute delay.

*Executive function.* Working memory was assessed using the Picture Memory task from the Wechsler Preschool and Primary Scale of Intelligence, fourth edition (WPPSI-IV) ^37^. The WPPSI-IV was designed for children aged 2.6 – 7.7 years old. The Picture Memory subtask uses the familiarise-recognise paradigm, wherein one or more stimuli are first viewed and then recognised from among an item set. Picture Memory has good test-retest reliability and excellent internal consistency in US samples in both the 2:5 – 3:11 and 4:0 – 7:7 age bands ^38^. Previous versions of the WPPSI have been used successfully in the Western Cape region to assess cognitive functioning ^39, 40^.

*Inhibition* was assessed using the Stroop-like Day-Night task ^41^. The task tests the ability to inhibit a prepotent response by requiring the child to say the opposite of what is shown on a set of cards. Two types of card are available in the deck: Children are instructed to say “day” when presented with a black moon card (night) card, and “night” when presented with a white sun card (day). The number of correct trials are scored. Studies of Day-Night task suggest that it has good internal consistency ^42^ and adequate test-retest reliability ^43, 44^.

*Cognitive flexibility* was assessed using the Dimensional Change Card Sort (DCSS) ^45, 46^, which resembles the Wisconsin Card Sorting Test (WCST). Participants are required to sort a series of cards, which contain two salient features/dimensions (e.g., colour and shape), first according to one dimension and then according to the other. The DCCS was chosen as it is appropriate for a wide age range and has excellent convergent validity and test-retest reliability ^47, 48^.

*Selective attention* was assessed using the Sky Search subtest from the Test of Everyday Attention for Children (TEA-Ch) ^49^. The child is instructed to find all the identical pairs on an A3 sheet with spacecraft. Completion time and number of errors are recorded. Sky Search has excellent test-retest reliability ^49^.

*Motor control* is assessed using the Fine Motor subtest of the Bayley-III (described above) ^23^ and the Grooved Pegboard (Lafayette Instrument Company, Inc.). The Bayley-III Fine Motor assesses visual tracking, reaching, perceptual-motor integration, and motor planning and speed. The task has good internal consistency as well as adequate test-retest reliability and convergent validity ^23, 50^. The Grooved Pegboard (Lafayette Instrument Company, Inc.) tests manipulative dexterity in the dominant and non-dominant hands. The task has 25 grooved holes arranged in rows. The participant must rotate pegs to insert them into the holes. The total completion time (in seconds) for each trial is recorded. The Grooved Pegboard test is reported to show excellent test-retest reliability for both hands and relatively strong concurrent validity in a 3-9 year old sample ^51^. The Grooved Pegboard has been used successfully in South African samples ^52^.

*Social cognition.* Theory of Mind (ToM) was examined via subtasks from the early and basic modules of the UCT Theory of Mind battery ^39, 53^. The early ToM module contains the Desires/Understanding Intentions and Perception-Leads-to-Knowledge tasks, as well as the Diverse Desires and Diverse Belief tasks ^54^. The Diverse Desires task assesses the ability to understand that a character can have desires different from one’s own, and that these desires will influence the choices they make. Similarly, the Diverse Beliefs assesses the ability to understand that a character can have beliefs different from one’s own, and that these beliefs will influence a character’s actions. The basic ToM module contains two tasks that assess false belief, namely the Location-Change False Belief and Unexpected Contents False Belief tasks, as well as the Belief Emotion and Hidden Emotions (Real/Apparent Emotion) tasks ^54^. The false belief tasks assess whether the child knows that a character can hold a belief that is not true, and that their actions will be based on their beliefs rather than the reality of the situation. Belief Emotion assesses whether the child can infer how a character will feel, given that the character’s belief is mistaken. Hidden Emotion assesses whether the child knows that a character can outwardly display a different emotion from what they are feeling. At age 3.5, only Diverse Desires was administered; at age 4.5, Diverse Desires and Diverse Beliefs was administered. At age 5, the remaining tasks from the early module, as well as all the tasks from the basic module, were administered. The tasks have been adapted and used successfully locally with low SES participants.

*Facial emotion recognition* was assessed using the NEPSY-II Affect recognition task ^55^. The stimuli feature a set of photographs of children from various race groups displaying a specific emotion. The participant is required to match the emotion to photographs of children displaying the same affect. As the task requires matching rather than labelling, it is less reliant on language. The task has been used successfully in local research with low SES participants ^53^.

**Socio-emotional development**

The socio-emotional assessment included measures of emotion regulation, affective arousal and social attention-allocation, empathy, morality, prosocial behaviour, temperament and callous-unemotional traits.

*Emotion regulation and effortful control* were captured through both observational and parent-report measures. Effortful control was observationally assessed with the Kochanska tasks ^56^, which have been designed for this purpose. The tasks assess delay of gratification, emotion regulation, and motor inhibition abilities. At age 3.5 and 4.5 years, variants of the Snack Delay task were used; at age 5, both the Snack Delay and Gift-in-Bag are used, alongside the Whisper task. The Snack Delay and Gift-in-Bag tasks assess the effortful control ability of delaying an action, while the Whisper task assesses voice lowering ability. In the variant of the Snack Delay task used at age 3.5, the researcher and child each place a smartie (a small sweet) on their tongues and compete to see who can refrain from eating it the longest. Additionally, the child is asked to hold a smartie on their tongue for four trials of 10, 20, 30, and 15s, respectively. The number of successful delays are coded. In the variant of the Snack Delay task used at 4.5 years, the child is given an unwrapped chocolate and told that if they do not eat it for the duration of the interview with the caregiver (15 minutes), they will be given two additional chocolates at the end. The time until child eats the chocolate is scored. In the Gift-in-Bag task, a colourfully-wrapped gift is placed in front of the child. The child is asked to stay seated not to touch the gift and the experimenter leaves the room for 3 minutes. The child is given scores for staying seated and for his or her behaviour with the gift, where higher scores indicate greater behavioural control. In the Whisper task, the experimenter shows the child a series of pictures of popular cartoon characters and asks him or her to whisper the name of each character. The child’s ability to modulate their voice is recorded. These tasks were chosen because they are simple to perform and rely very little on language. From 33 months of age, the effortful control tasks show adequate consistency across tasks and have good longitudinal stability in a US sample ^56, 57^.

Parent-report of emotion regulation was captured using Rydell’s Emotion Questionnaire ^58^*.* This 16-item short form measures the child’s typical reaction to and regulation of sadness, anger, fear and exuberance. Scale totals are calculated by taking the mean of the item scores. Depending on the scale, high scores indicate either high levels of emotionality or high levels of emotion regulation ability. Good construct validity and test-retest reliability has been reported previously ^58^.

*Attentional*-*allocation* *and affective arousal to visual emotional stimuli* was assessed in a subgroup of children using eye gaze and pupil dilation measures. The eye tracking assessments were conducted in a research laboratory using a remote Tobii Studio X60 eye tracker (Tobii Technology, 2010)(60 Hz acquisition rate). Participants are shown pictures of infant faces with either positive (smiling), negative (crying) or neutral expressions ^59^. The stimuli feature Black, White and Mixed Race faces. The timing, duration and location of fixations are determined for each stimulus, and are used to index attentional-allocation. Pupil dilation during the stimulus is used as an index of autonomic arousal, and has been shown to correlate strongly with sympathetic arousal in particular ^60^. The use of eye tracking is particularly well-suited to child studies, as it does not require advanced language or motor responses, and is a non-invasive and objective measure of arousal and attention ^61, 62^. In addition, visual stimuli of facial expressions depicting various emotions is frequently used to induce arousal and measure attention-allocation ^63, 64^.

*Maternal and child empathy.* Self-reported (child) empathy was assessed via the Chicago empathy for pain task ^65^. The task features everyday situations depicting either a painful or a neutral (non-painful) event, and is suitable for children aged 3 years and up. To assess affective empathy, participants are asked to rate how much pain they think the character is experiencing on a visual analogue scale. To assess empathic concern, participants rate how sorry they the feel for the character. Participant responses are coded as 1 – 100, where 100 is the maximum possible pain/ concern. Empathic concern and affective empathy scores are calculated as the mean response over all trials (36 trials; 18 pain and 18 non-pain). Maternal empathy and child dispositional empathy were assessed with the Questionnaire of Cognitive and Affective Empathy (QCAE) ^66^. The QCAE is completed by the mother/caregiver and contains 31 questions assessing either cognitive or affective empathy. Mothers/caregivers rate the degree to which each item describes them (maternal empathy) and their child (child empathy); higher scores indicate greater empathy. The subscales of the QCAE have shown adequate to good internal consistency in low SES samples in South Africa ^67, 68^. Maternal dispositional empathy impacts on the dyadic mother-child relationship, and is thought to have some influence on the child’s developing empathy. This in turn impacts on the child’s social interactions overall, and hence on the developing socioemotional and social cognitive skill set.

*Prosocial behaviour* was assessed using a simple sharing task, a variation on the Dictator Game. The child chooses a set of desirable items (attractive stickers), and is asked if they would like to share with their siblings or friends. The child is instructed to place the items to be given away in a separate container. To avoid social-desirability bias, the administrator turns his/her back while the child does the sharing. Prosocial behaviour is coded as the number of items shared. This task has been used successfully in local research ^67^.

*Internalising and externalising behaviour* was assessed with the parent-report version of the Child Behaviour Checklist (CBCL) ^69^. The measure assesses child social competence in the school, social and activities domains, and the presence of internalising and externalising behaviours in the last 6 months. High scores on the CBCL that indicate the presence of problem behaviours and inadequate competencies in relation to age and gender-matched peers. The measure is widely used internationally, and has proved reliable in South Africa ^70, 71^.

*Temperament* was assessed via short forms of the widely-used Rothbart questionnaires. The Rothbart Infant Behaviour Questionnaire-Revised, very short form, is used to acquire retrospective report on infant temperament at 3.5 years. The Rothbart Early Child Behaviour Questionnaire, very short form ^72^, was used to measure current temperament at 4.5 years ^73^. The questionnaires measure negative emotionality, positive affect, and orienting/regulatory capacity. The very short forms have shown adequate internal consistency and high correlations with the standard forms ^72, 73^, and have been used in low-resource African settings ^74^.

*Presence of callous-unemotional traits* was assessed using the Callous Unemotional Screening Device, a 9-item parent-report measure based on Dadds et al. ^75^. The screening device combines items related to callous-unemotional traits from the Strengths and Difficulties Questionnaire (SDQ) ^76, 77^ and the Antisocial Process Screening Device (APSD) ^78^. The measure has demonstrated adequate internal consistency in local research with low SES participants ^71^. It was felt to be important to include a measure of callous-unemotional personality traits in this cohort, as these traits are influenced by early exposure to adversity and the nature of the child’s primary relationships and predict risk for psychopathology in adulthood ^79, 80^.

**References**

1. Stein D, Koen N, Donald K, et al. Investigating the psychosocial determinants of child health in Africa: The Drakenstein Child Health Study. *J Neurosci Methods* 2015; **252**: 27-35.

2. Saylor CF, Swenson CC, Stokes Reynolds S, Taylor M. The pediatric emotional distress scale: A brief screening measure for young children exposed to traumatic events. *J Clin Child Psychol* 1999; **28**: 70-81.

3. Martin L, Revington N, Seedat S. The 39-Item Child Exposure to Community Violence (CECV) Scale: Exploratory factor analysis and relationship to PTSD symptomatology in trauma-axposed children and adolescents. *Int J Behav Med* 2013; **20**: 599-608.

4. Engle PL, Fernald LC, Alderman H, et al. Strategies for reducing inequalities and improving developmental outcomes for young children in low-income and middle-income countries. *The Lancet* 2011; **378**: 1339-53.

5. Walker SP, Wachs TD, Grantham-McGregor S, et al. Inequality in early childhood: risk and protective factors for early child development. *The Lancet* 2011; **378**: 1325-38.

6. Walker SP, Wachs TD, Meeks Gardner J, et al. Child development: risk factors for adverse outcomes in developing countries. *The Lancet* 2007; **369**: 145-57.

7. DeCou CR, Lynch SM. Assessing adult exposure to community violence: A review of definitions and measures. *Trauma Violence Abuse* 2017; **18**: 51-61.

8. Richters JE, Saltzman W. *Survey of exposure to community violence: Self-report version*: JE Richters; 1990.

9. Murray L, Fiori-Cowley A, Hooper R, Cooper P. The impact of postnatal depression and associated adversity on early mother-infant interactions and later infant outcome. *Child Dev* 1996; **67**: 2512-26.

10. Biringen Z. *The emotional availability (EA) scales*. Fourth ed. Boulder, CO; 2008.

11. Humber N, Moss E. The relationship of preschool and early school age attachment to mother-child interaction. *Am J Orthopsychiatry* 2005; **75**: 128-41.

12. Kim S, Kochanska G. Child temperament moderates effects of parent–child mutuality on self-regulation: A relationship-based path for emotionally negative infants. *Child Dev* 2012; **83**: 1275-89.

13. Biringen Z. Emotional availability: Conceptualization and research findings. *Am J Orthopsychiatry* 2000; **70**: 104-14.

14. Bain K. "New beginnings" in South African shelters for the homeless: Piloting of a group psychotherapy intervention for high-risk mother-infant dyads. *Infant Ment Health J* 2014; **35**: 591-603.

15. Gul H, Erol N, Pamir A, et al. Emotional availability in early mother-child interactions for children with autism spectrum disorders, other psychiatric disorders, and developmental delay. *Infant Ment Health J* 2016; **37**: 151-9.

16. van Ee E, Kleber RJ, Mooren TTM. War trauma lingers on: Associations between maternal posttraumatic stress disorder, parent-child interaction, and child development. *Infant Ment Health J* 2012; **33**: 459-68.

17. Sanders MR, Morawska A, Haslam DM, Filus A, Fletcher R. Parenting and Family Adjustment Scales (PAFAS): Validation of a brief parent-report measure for use in assessment of parenting skills and family relationships. *Child Psychiatry Hum Dev* 2014; **45**: 255-72.

18. Brockington IF, Oates J, George S, et al. A Screening Questionnaire for mother-infant bonding disorders. *Arch Womens Ment Health* 2001; **3**: 133-40.

19. Brockington IF, Fraser C, Wilson D. The Postpartum Bonding Questionnaire: a validation. *Arch Womens Ment Health* 2006; **9**: 233-42.

20. Rotheram-Borus MJ, Richter LM, Heerden Av, et al. A cluster randomized controlled trial evaluating the efficacy of peer mentors to support South African women living with HIV and their infants. *PLoS One* 2014; **9**: e84867.

21. Ungar M, Liebenberg L. Assessing resilience across cultures using mixed methods: Construction of the child and youth resilience measure. *J Mix Methods Res* 2011; **5**: 126-49.

22. Liebenberg L, Ungar M, Vijver FVd. Validation of the Child and Youth Resilience Measure-28 (CYRM-28) among Canadian youth. *Res Soc Work Pract* 2012; **22**: 219-26.

23. Bayley N. *Bayley Scales of Infant and Toddler Development*. Third ed. San Antonio, TX: Psychological Corporation; 2006.

24. Albers CA, Grieve AJ. Review of Bayley Scales of Infant and Toddler Development--Third Edition. *J Psychoeduc Assess* 2007; **25**: 180-90.

25. Ballot DE, Potterton J, Chirwa T, Hilburn N, Cooper PA. Developmental outcome of very low birth weight infants in a developing country. *BMC Pediatr* 2012; **12**: 11.

26. Lichtenberger EO, Sotelo-Dynega M. The Kaufman Assessment Battery for Children—Second Edition. *Practitioner's Guide to Assessing Intelligence and Achievement* 2009: 61.

27. Kaufman AS, Lichtenberger EO, Fletcher-Janzen E, Kaufman NL. *Essentials of KABC-II assessment*. Hoboken, NJ: John Wiley & Sons; 2005.

28. Baumgartner J, Smuts CM, Malan L, et al. Effects of iron and n−3 fatty acid supplementation, alone and in combination, on cognition in school children: a randomized, double-blind, placebo-controlled intervention in South Africa. *Am J Clin Nutr* 2012; **96**: 1327-38.

29. Boivin MJ, Vokhiwa M, Sikorskii A, Magen JG, Beare N. Cerebral malaria retinopathy predictors of persisting neurocognitive outcomes in Malawian children. *Pediatr Infect Dis J* 2014.

30. Greenop K, Fry J, de Sousa D. The Kaufman Assesment Battery in South Africa. In: Laher SC, K., editor. *Psychological assessment in South Africa: Research and Applications*. Johannesburg: Wits University Press; 2013. p. 86-103.

31. Ruel TD, Boivin MJ, Boal HE, et al. Neurocognitive and motor deficits in HIV-infected Ugandan children with high CD4 cell counts. *Clin Infect Dis* 2012; **54**: 1001-9.

32. Taljaard C, Covic NM, Van Graan AE, et al. Effects of a multi-micronutrient-fortified beverage, with and without sugar, on growth and cognition in South African schoolchildren: a randomised, double-blind, controlled intervention. *Br J Nutr* 2013; **110**: 2271-84.

33. Dunn LM, Dunn DM. *PPVT-4: Peabody Picture Vocabulary Test*. Minneapolis, MN: Pearson Assessments; 2007.

34. Kaufman AS, Kaufman NL. *KABC-II: Kaufman Assessment Battery for Children*. Circle Pines, MN: AGS Pub.; 2004.

35. Dawes A, Biersteker L, Hendricks L. *Towards integrated early childhood development. An evaluation of the Sobambisana Initiative*. ilifa labantwana 2012 [cited 2017 12 August]; Available from: http://ilifalabantwana.co.za/an-evaluation-of-the-sobambisana-initiative

36. Kaufman AS, Kaufman NL. Kaufman Assessment Battery for Children, Second Edition. *Encyclopedia of Special Education*. Hoboken, NJ: John Wiley & Sons, Inc.; 2013.

37. Wechsler D. *WPPSI-IV: Wechsler Preschool and Primary Scale of Intelligence: Manual*. London, United Kingdom: Pearson Education; 2012.

38. Williams ME, Sando L, Soles TG. Cognitive tests in early childhood: Psychometric and cultural considerations. *J Psychoeduc Assess* 2014; **32**: 455-76.

39. Hoogenhout M, Malcolm-Smith S. Theory of mind in autism spectrum disorder: Does DSM classification predict development? *Res Autism Spectr Disord* 2014; **8**: 597-607.

40. Pileggi L-A, Malcolm-Smith S, Solms M. Investigating the role of social-affective attachment processes in cradling bias: The absence of cradling bias in children with Autism Spectrum Disorders. *Laterality* 2015; **20**: 154-70.

41. Gerstadt CL, Hong YJ, Diamond A. The relationship between cognition and action: performance of children 312–7 years old on a Stroop- like day-night test. *Cognition* 1994; **53**: 129-53.

42. Rhoades BL, Greenberg MT, Domitrovich CE. The contribution of inhibitory control to preschoolers' social–emotional competence. *J Appl Dev Psychol* 2009; **30**: 310-20.

43. Hughes C, Ensor R. Does executive function matter for preschoolers’ problem behaviors? *J Abnorm Child Psychol* 2008; **36**: 1-14.

44. Thorell LB, Wåhlstedt C. Executive functioning deficits in relation to symptoms of ADHD and/or ODD in preschool children. *Infant Child Dev* 2006; **15**: 503-18.

45. Frye D, Zelazo PD, Palfai T. Theory of mind and rule-based reasoning. *Cogn Dev* 1995; **10**: 483-527.

46. Zelazo PD. The Dimensional Change Card Sort (DCCS): A method of assessing executive function in children. *Nat Protoc* 2006; **1**: 297-301.

47. Beck DM, Schaefer C, Pang K, Carlson SM. Executive function in preschool children: Test–retest reliability. *J Cogn Dev* 2011; **12**: 169-93.

48. Zelazo PD, Bauer PJ, Fox NA. *National Institutes of Health Toolbox Cognition Battery (NIH Toolbox CB): Validation for children between 3 and 15 years*: Wiley; 2013.

49. Manly T, Anderson V, Nimmo-Smith I, Turner A, Watson P, Robertson IH. The differential assessment of children's attention: The Test of Everyday Attention for Children (TEA-Ch), normative sample and ADHD performance. *J Child Psychol Psychiatry* 2001; **42**: 1065-81.

50. Folio MR, Fewell RR. *Peabody developmental motor scales: Examiner's manual*: Pro-ed; 2000.

51. Wang Y-C, Magasi SR, Bohannon RW, et al. Assessing dexterity function: a comparison of two alternatives for the NIH Toolbox. *J Hand Ther* 2011; **24**: 313-21.

52. Meyer A, Sagvolden T. Fine motor skills in South African children with symptoms of ADHD: influence of subtype, gender, age, and hand dominance. 2006; **2**: 33.

53. Lindinger NM, Malcolm-Smith S, Dodge NC, et al. Theory of mind in children with fetal alcohol spectrum disorders. *Alcohol Clin Exp Res* 2016; **40**: 367-76.

54. Wellman HM, Liu D. Scaling of theory‐of‐mind tasks. *Child Dev* 2004; **75**: 523-41.

55. Korkman M, Kirk U, Kemp S. *NEPSY II Clinical & Interpretative Manual*. San Antonio, Texas: The Psychological Corporation; 2007.

56. Kochanska G, Murray KT, Harlan ET. Effortful control in early childhood: Continuity and change, antecedents, and implications for social development. *Dev Psychol* 2000; **36**: 220-32.

57. Murray KT, Kochanska G. Effortful control: Factor structure and relation to externalizing and internalizing behaviors. *J Abnorm Child Psychol* 2002; **30**: 503-14.

58. Rydell A-M, Berlin L, Bohlin G. Emotionality, emotion regulation, and adaptation among 5- to 8-year-old children. *Emotion* 2003; **3**: 30-47.

59. Yrttiaho S, Niehaus D, Thomas E, Leppänen JM. Mothers’ pupillary responses to infant facial expressions. *Behav Brain Func* 2017; **13**: 2.

60. Bradley MM, Miccoli L, Escrig MA, Lang PJ. The pupil as a measure of emotional arousal and autonomic activation. *Psychophysiology* 2008; **45**: 602-7.

61. Jackson I, Sirois S. Infant cognition: going full factorial with pupil dilation. *Dev Sci* 2009; **12**: 670-9.

62. Laeng B, Sirois S, Gredebäck G. Pupillometry: A window to the preconscious? *Perspect Psychol Sci* 2012; **7**: 18-27.

63. Kylliäinen A, Hietanen JK. Skin conductance responses to another person’s gaze in children with autism. *J Autism Dev Disord* 2006; **36**: 517-25.

64. Kylliäinen A, Wallace S, Coutanche MN, et al. Affective–motivational brain responses to direct gaze in children with autism spectrum disorder. *J Child Psychol Psychiatry* 2012; **53**: 790-7.

65. Decety J, Michalska KJ, Akitsuki Y. Who caused the pain? An fMRI investigation of empathy and intentionality in children. *Neuropsychologia* 2008; **46**: 2607-14.

66. Reniers RL, Corcoran R, Drake R, Shryane NM, Völlm BA. The QCAE: A questionnaire of cognitive and affective empathy. *J Pers Assess* 2011; **93**: 84-95.

67. Decety J, Cowell Jason M, Lee K, et al. The negative association between religiousness and children’s altruism across the world. *Curr Biol* 2015; **25**: 2951-5.

68. Nefdt K. *Empathy across socioeconomic status and its association with aggressive behaviour in Western Cape children* [Unpublished honours thesis]. Cape Town, South Africa: University of Cape Town; 2013.

69. Achenbach TM, Rescorla LA. *Manual for the ASEBA Preschool Forms & Profiles*. Burlington, Vermont: University of Vermont, Research Center for Children, Youth & Families; 2001.

70. Barbarin OA, Richter L, deWet T. Exposure to violence, coping resources, and psychological adjustment of South African children. *Am J Orthopsychiatry* 2001; **71**: 16-25.

71. Malcolm-Smith S, Woolley D, Ward CL. Examining empathy and its association with aggression in young Western Cape children. *J* *Child Adolesc Ment Health* 2015; **27**: 135-47.

72. Putnam SP, Helbig AL, Gartstein MA, Rothbart MK, Leerkes E. Development and assessment of Short and Very Short Forms of the Infant Behavior Questionnaire–Revised. *J Pers Assess* 2014; **96**: 445-58.

73. Putnam SP, Rothbart MK. Development of Short and Very Short Forms of the Children's Behavior Questionnaire. *J Pers Assess* 2006; **87**: 102-12.

74. Gartstein MA, Bogale W, Meehan CL. Adaptation of the Infant Behavior Questionnaire-Revised for use in Ethiopia: Expanding cross-cultural investigation of temperament development. Infant *Behav Dev* 2016; **45, Part A**: 51-63.

75. Dadds MR, Fraser J, Frost A, Hawes DJ. Disentangling the underlying dimensions of psychopathy and conduct problems in childhood: a community study. *J Consult Clin Psychol* 2005; **73**: 400-10.

76. Goodman R. The strengths and difficulties questionnaire: A research note. *J Child Psychol Psychiatry* 1997; **38**: 581-6.

77. Goodman R, Meltzer H, Bailey V. The strengths and difficulties questionnaire: A pilot study on the validity of the self-report version. *Eur Child Adolesc Psychiatry* 1998; **7**: 125-30.

78. Frick PJ, Hare RD. *Antisocial process screening device: APSD.* Toronto: Multi-Health Systems; 2001.

79. Frick PJ, White SF. Research Review: The importance of callous-unemotional traits for developmental models of aggressive and antisocial behavior. *J Child Psychol Psychiatry* 2008; **49**: 359-75.

80. Viding E, Price TS, Jaffee SR, et al. Genetics of callous-unemotional behavior in children. *PLoS One* 2013; **8**: e65789.
