# Supplementary material for: Drakenstein Child Health Study (DCHS): investigating determinants of early child development and cognition
Source: BMJ Paediatr Open. 2018 Jun 13;2(1):e000282. doi: 10.1136/bmjpo-2018-000282 (PMC6014194; doi:10.1136/bmjpo-2018-000282)
Supplement: Author's manuscript [file bmjpo-2018-000282.draft_revisions.pdf]

BMJ Paediatrics Open

BMJ Paediatrics Open is committed to open peer review. As part of this commitment we make the peer review history of every article we publish publicly available.

When an article is published we post the peer reviewers' comments and the authors' responses online. We also post the versions of the paper that were used during peer review. These are the versions that the peer review comments apply to.

The versions of the paper that follow are the versions that were submitted during the peer review process. They are not the versions of record or the final published versions. They should not be cited or distributed as the published version of this manuscript.

BMJ Paediatrics Open is an open access journal and the full, final, typeset and author-corrected version of record of the manuscript is available on our site with no access controls, subscription charges or pay-per-view fees (http://bmjpaedsopen.bmj.com).

If you have any questions on BMJ Paediatrics Open's open peer review process please email info.bmjpo@bmj.com

BMJ Paediatrics Open

Confidential

Protocol: The Drakenstein Child Health Study (DCHS)- Investigating determinants of early child development and cognition

Journal:	*BMJ Paediatrics Open*
Manuscript ID	bmjpo-2018-000282
Article Type:	Protocol
Date Submitted by the Author:	01-Mar-2018
Complete List of Authors:	Donald, Kirsten; University of Cape Town Department of Paediatrics and Child Health, Division of Developmental Paediatrics<br>Hoogenhout, Michelle; University of Cape Town Department of Paediatrics and Child Health, Division of Developmental Paediatrics<br>du Plooy, Christopher; University of Cape Town Department of Paediatrics and Child Health, Division of Developmental Paediatrics<br>Wedderburn, Catherine; University of Cape Town Department of Paediatrics and Child Health, Division of Developmental Paediatrics; London School of Hygiene and Tropical Medicine Department of Clinical Research<br>Nhapi, Raymond; South African Medical Research Council, Unit on Child & Adolescent Health<br>Barnett, Whitney; South African Medical Research Council, Unit on Child & Adolescent Health; University of Cape Town Department of Paediatrics and Child Health<br>Hoffman, Nadia; UCT Department of Psychiatry and Mental Health<br>Malcolm-Smith, Susan; University of cape town Department of Psychology, Applied Cognitive Science and Experimental Neuropsychology Team<br>Zar, Heather; South African Medical Research Council, Unit on Child & Adolescent Health; University of Cape Town Department of Paediatrics and Child Health<br>Stein, Dan; UCT Department of Psychiatry and Mental Health; South African Medical Research Council, Unit on Risk and Resilience in Mental Disorders
Keywords:	Neurodevelopment

SCHOLARONE™
Manuscripts

Protocol: The Drakenstein Child Health Study (DCHS)-Investigating determinants of early child development and cognition

Author List: Kirsten A Donald[1], Michelle Hoogenhout[1], Christopher P du Plooy[1], Catherine J Wedderburn[1,2], Raymond T Nhapi[3], Whitney Barnett[3,4], Nadia Hoffman[5], Susan Malcolm-Smith[6], Heather J Zar[3,4], Dan J Stein[5,7].

Affiliation:

[1]Division of Developmental Paediatrics, Department of Paediatrics & Child Health, Red Cross War Memorial Children's Hospital, University of Cape Town, South Africa

[2]Department of Clinical Research, London School of Hygiene & Tropical Medicine, UK

[3]South African Medical Research Council (SAMRC), Unit on Child & Adolescent Health, Cape Town, South Africa

[4]Department of Paediatrics & Child Health, Red Cross War Memorial Children's Hospital, University of Cape Town, South Africa

[5]Department of Psychiatry & Mental Health, University of Cape Town, South Africa

[6]Applied Cognitive Science and Experimental Neuropsychology Team, Department of Psychology, University of Cape Town, South Africa

[7]South African Medical Research Council (SAMRC), Unit on Risk and Resilience in Mental Disorders, Cape Town, South Africa

Corresponding author:

Kirsten A Donald

Email: Kirsty.Donald@uct.ac.za

Phone: 021 6585322

Word count: 2325

Confidential: For Review Only

What is known about the subject?

There is growing awareness that psychosocial risk and resilience factors in early life play a key role in influencing later health.

Most work has been done in high income settings, rather than low- and middle-income countries (LMICs), where the majority of the global childhood population resides.

The few studies with well-defined cohorts in LMICs have employed various methods and measures, making comparisons across studies challenging.

What this study hopes to add

The Drakenstein Child Health Study (DCHS) aims to provide an understanding of the effects of multiple risk and mitigating factors on child health and development in a LMIC.

For Review Only

Abstract

Introduction: There is growing awareness that psychosocial risk and resilience factors in early life play a key role in influencing later health. Most work has been done in high income settings, rather than low- and middle-income countries (LMICs), where the majority of the global childhood population resides. The few studies with well-defined cohorts in LMICs have employed various methods and measures, making comparisons across studies challenging. This presentation describes the methodology for infant and child developmental measures used in the Drakenstein Child Health Study (DCHS), a multi-disciplinary longitudinal birth cohort study in South Africa.

Methods and analysis. We outline a multilevel approach combining a range of measures including parental reports, behaviour observations, clinician-administered scales and brain imaging. Using this approach, we aim at a longitudinal perspective of developmental, cognitive, socioemotional and neurophysiological outcomes in a birth cohort of children in a LMIC.

Ethics and dissemination: The study was approved by the faculty of Health Sciences, Human Research Ethics Committee, University of Cape Town (401/2009), by Stellenbosch University (N12/02/0002), and the Western Cape Provincial Health Research committee (2011RP45).

Discussion: Children in the DCHS develop in a context typical of many communities in South Africa and other LMICs. There is a critical need for research in LMICs to elucidate underlying factors that inform risk for, and resilience to, poor developmental outcomes in infants born into high risk communities. Such work may inform effective intervention strategies appropriate to this context.

Keywords: global health, child development, assessment, cognition, socio-emotional development, resilience

Confidential: For Review Only

Introduction

Risk and resilience factors encountered during the early years of life have an enduring influence on later physiological and psychological outcomes [1-3]. A number of risk factors are already apparent in utero; for example, antepartum maternal psychological distress and depression can adversely affect infant physical, neurocognitive and socioemotional developmental outcomes [4-6]. During early childhood, exposure to stressors such as familial violence and abuse has been associated with increased risk of behaviour problems, autoimmune disorders, cardiovascular disease, and premature mortality [7-10]. In LMICs, key risk factors such as HIV infection and prenatal maternal malnutrition are responsible for millions of children failing to reach their full developmental potential [11-13]. Poor child outcomes may have intergenerational effects, so exacerbating their impact [12].

At the same time, protective factors may be associated with increased resilience, and so with positive mental health and developmental outcomes in the face of stressors [14]. Resilience is thought to arise from the interplay between factors at the individual, family and community levels [15]. Protective factors can be highly context-specific and can exert different effects at different timepoints [15 16]. Thus, longitudinal studies provide the best opportunities to identify protective factors at various stages of development, as well as sensitive periods for intervention. However, most studies on resilience have been done in high-income countries, where contextual factors may be different.

Indeed, the vast majority of previously reported studies have focused on psychobiological and psychosocial risk profiles in well-resourced countries. These profiles differ considerably in LMICs. For instance, there is, in general, considerably higher prevalence of low birth weight, childhood malnutrition and infectious diseases in LMICs [17 18]. In addition, critical psychosocial factors that are known to have impact on child development

Confidential: For Review Only

such as maternal depression and exposure to violence frequently have a greater prevalence in these high risk communities [12 13 19]. There is considerable work in LMICs, including work that is longitudinal and that is culturally appropriate. However, various methods and measures haven been used, so that cross-cohort comparison is not always possible.

The Drakenstein Child Health Study (DCHS) is a multi-disciplinary longitudinal study investigating the early life determinants of child health in two peri-urban communities in the Western Cape Province, South Africa [20]. The early-life component focuses on a broad spectrum of child health outcomes, including physical health and growth as well as neurodevelopmental, cognitive and psychological health. The study investigates the role and interaction of environmental, infectious, psychosocial, nutritional, genetic, maternal and immunological risk and protective factors for development. The DCHS follows an extensively phenotyped cohort over critical early years, aiming to provide an understanding into the effects of multiple risk and mitigating factors, and their interactions, on child health and development in a LMIC. This paper describes the methodology for the infant and child measures of the psychosocial component of the DCHS. By documenting the measures used and our reasons for choosing these, we hope to improve future harmonisation between cohort studies.

Methods and analysis

Design and Setting

The DCHS is located in the peri-urban Drakenstein district, Western Cape, South Africa. The communities surrounding the Mbekweni and TC Newman clinics are ethnically, culturally and linguistically heterogeneous. Mbekweni consists of a mostly isiXhosa-speaking black African population, whereas TC Newman consists of a mostly Afrikaans-speaking mixed

Confidential: For Review Only

race population [20]. However, both communities are characterised by low socio-economic status and feature a high prevalence of multiple psychosocial risk factors; including single parent households, high rates of psychological distress, and exposure to violence, HIV and substance abuse[21]. In particular, risk factors that may be highly prevalent in these communities and similar communities in the region include high rates of HIV exposure [20 22 23], high prevalence of maternal psychological distress and depression [5 21 24-26], high rates of drug and alcohol usage [21 24 27 28], high levels of violence and intimate partner violence [29], and low levels of employment and educational attainment [21]. The population is stable, with little immigration or emigration. More than 90% of people in the district use the public health system. In this regard, the communities are representative of many other communities in South Africa and other low and middle-income countries (LMICs).

Participants

Pregnant women were recruited while attending routine antenatal care at Mbekweni or TC Newman clinic between March 2012 and March 2015. Women were enrolled in the DCHS at 20 to 28 weeks' gestation and were followed through birth and postnatally. Pregnant mothers were eligible for the study if they were 18 years or older, planned to attend antenatal care at one of the two clinics and intended to remain in the area for at least a year. Expecting mothers provided informed written consent at enrolment and were re-consented annually following childbirth.

Mothers were provided informed consent in their preferred language, English, Afrikaans or isiXhosa, by trained study staff from the community. Informed consent forms described the scope and aims of the study, including potential harm or benefits. In total, 1137 mother-child dyads were enrolled in the study; of which four mothers had twins and one had

Confidential: For Review Only

triplets. Thus, 1143 children were enrolled in the study. A conservative cumulative attrition of 20% over five years was estimated, and the sample size was calculated accordingly.

Measures

Mothers were followed during pregnancy and childbirth. Following birth, infants and mothers returned to the clinics and were asked to complete self-report and clinician-administered measures at time points ranging from 2-4 weeks to 5 years (see Figure 1). At the time of submission, the oldest children in the cohort are 5 years old and the youngest children are currently 2 years old.

[Fig. 1 here]

The infant and child developmental and psychosocial measures are described here. The overview methods of the larger DCHS are described elsewhere [20], as is the maternal and paternal psychosocial evaluation component of the study [21]. Broadly, the child measures assessed (1) social and biological risk and protective factors, (2) general neurobiological development, (3) cognitive development, and (4) socio-emotional development. These measures were chosen for their ability to identify multivariate risk and protective factors for child health outcomes. All measures were administered by trained research assistants, with the aid of trained interpreters when necessary. Measures were translated from English to isiXhosa and Afrikaans using the standard forward and backwards-translation method [30]. The tests used are detailed in Table 1 with detailed description in supplementary appendix.

[Table 1 here]

Confidential: For Review Only

Descriptive statistics

We use descriptive statistics (medians, interquartile ranges, counts & percentages) to present the sociodemographic variables of the cohort. These statistics are presented on the two study sites (Mbekweni and TC Newman) as well as the overall cohort. Chi-Square and Mann-Whitney tests were used to test for differences between the sites. As the study is still ongoing, the data presented here was collected antenatally and at birth.

Child sociodemographic characteristics

The cohort followed 1137 mothers over the course of three years during the initial recruitment period. During this time there was a total of 1143 live births including 4 sets of twins and 1 set of triplets (see Table 2). Within this group, 17.2% of children were born preterm (defined here as less than 37 weeks' gestation). The birth weight and lengths of the children have been converted to z-scores according to WHO standardisation and adjusted for gestation. In line with our previous reports [31 32], the children at TC Newman clinic were born significantly smaller in weight compared to Mbekweni children; however, the IQR falls within two z-scores of 0 in both clinics.

[Table 2 here]

Maternal and family sociodemographic characteristics

The maternal and family characteristics are presented in Table 3. Socioeconomic status was low across both clinics as shown by the low household income levels, maternal employment rates of 27% and maternal educational attainment. The median maternal age at enrolment was 26 years (IQR 22–31) and the majority of mothers had completed some secondary schooling by this point. The mothers reported that over 65% of pregnancies were

Confidential: For Review Only

unplanned. Approximately 40% were currently married or cohabiting with their partner, and a high proportion reported that partners were supportive, although this differed across clinics.

[Table 3 here]

Mothers and children in both communities were frequently exposed to community violence (see Table 4). Levels of violence exposure were higher in TC Newman than in Mbekweni, but both communities from this cohort reported greater exposure to violence than reported in a previous study in South Africa [33]. Exposure to violence is thus highly prevalent in these communities both within the home and external community environment. Both communities reported less consistent parenting (i.e. higher consistency scores), but otherwise similar parenting styles and family adjustment, compared to European samples [34]. Mothers in Mbekweni reported more coercive parenting, less consistency and less encouragement, but also better family relationships and parental teamwork than in TC Newman.

[Table 4 here]

Confidential: For Review Only

Ethics and dissemination

The study was approved by the faculty of Health Sciences, Human Research Ethics Committee, University of Cape Town (401/2009), by Stellenbosch University (N12/02/0002), and the Western Cape Provincial Health Research committee (2011RP45).

Confidential: For Review Only

Discussion

This paper highlights the rationale and approach to assessing both psychosocial risk and protective factors impacting the development of children in a high-risk South African communities. The study follows a multilevel approach that targets developmental, cognitive, socioemotional and physiological outcomes. . As can be seen from some of our baseline data (*n* = 1143), there are a broad number of sociodemographic risk and resilience factors for children in this region. Demographic and sociodemographic data show that although these communities are in close proximity, they differ substantively in social and financial resources.

Given these sorts of risk and resilience factors, it is important to assess child outcomes using a multidimensional approach [35]. This includes three important components which are built into this cohort. Firstly, the DCHS collects biomedical and psychosocial risk factors across a wide range of factors in the prenatal period and first years of life. These include both factors which are known to put children at risk for poor outcome such as maternal mental health, substance use disorders, poor nutrition and factors known to be protective or hypothesised to potentially support development including early infant feeding choices, pregnancy support and maternal bonding and attachment styles. Secondly, the outcome measures described in this manuscript are also multidimensional and allow examination of outcomes in terms of the dyadic relationship and the family system into which these children are born. Thirdly, the cohort is following these mother infant pairs over time. Longitudinal data (with repeat measures) allows the investigation of developmental, cognitive and socio-emotional trajectories as well as the interactions between exposures within the context of this cohort. The investigation of the timing of maximum windows of vulnerability becomes possible with this approach.

Confidential: For Review Only

Attention to the ethical issues requires consideration in a study of this type. The DCHS maintains an active programme focused on community engagement, including regular engagement with study participants for feedback on study involvement, active dissemination of research results to key local stakeholders and distribution of health promotion information to study participants. Given the context, a key ethical obligation includes screening within the study population for physical and mental health issues, in both mothers and children. Screening is done in the DCHS in conjunction with an active referral system and is bolstered by close relationships between study staff and provincial health staff. All women involved in the study, independent of identified mental or physical health problems, receive information regarding service providers in the area. The network of investigators in the DCHS with strong and relevant clinical background in the South African public health environment is a strength of the research and has allowed realistic and integrated referral systems to be developed and implemented as part of the study.

Very few cohorts are reported which take into account the composite effects of multiple factors on health and development in the early years. This is especially true of cohorts from LMICs where young children are exposed to overlapping epidemics of infectious and non-communicable diseases. Sensitivity to exposures, individually and together, in both external and internal environments are different at specific ages and periods in development. The developmental window spanning the 40 weeks of pregnancy and the first years of life appear to be a critical period where environmental exposure may cause embedded effects which may have impact across the lifespan. There is a critical need for research in this field to elucidate the underlying factors that inform risk for and resilience from poor developmental outcomes in infants born into high risk communities which may ultimately inform effective preventative and ameliorating intervention strategies appropriate to this context. From a public health perspective, a better understanding of the relevant mechanisms is critical, as this

Confidential: For Review Only

may ultimately drive preventative and targeted therapeutic approaches. The United Nation's Sustainable Development Goals (SDGs) were officially adopted in 2015. These cross cutting goals consistently forefront the importance of addressing programmes for targeting maternal and child health, and in particular the theme of early child growth and development, as being key in the global strategy to optimize human health and well-being. In the South African setting, where children make up around a third of the population expectant mothers and their young infants are a particularly important focus. Much more attention is needed to address maternal and infant health, in order to decrease early mortality and later morbidity in this vulnerable population.

Limitations

Limitations of the study include the fact that though extremely well characterised, the cohort is a modest size. Though measures investigating aspects of child health, development and cognition have been administered to as much of the cohort as possible, certain measures have been administered only on a subset of the group (e.g., neuroimaging) due to participant burden and the cost of assessment. Although care was taken to translate measures into Afrikaans and isiXhosa, there will always be some difficulty in interpreting the results of measures designed in a different language and cultural context. Every effort was made to use tools which minimised problems in this area.

Confidential: For Review Only

Data sharing statement

Collaborations for the analysis of data are welcome. The DCHS has a large and active group of investigators and postgraduate students and many have successfully partnered with researchers from other institutions. In particular, we encourage collaborations that lead to skills transfer and capacity building for postgraduate students. Researchers who are interested in datasets or collaborations can contact the PI, Heather Zar [heather.zar@uct.ac.za] with a concept note outlining the request. More information can be found on our website [http://www.paediatrics.uct.ac.za/scah/dclhs].

Confidential: For Review Only

References

1. Miller GE, Chen E, Parker KJ. Psychological stress in childhood and susceptibility to the chronic diseases of aging: moving toward a model of behavioral and biological mechanisms. *Psychol Bull* 2011;137(6):959.
2. Gluckman PD, Hanson MA, Cooper C, et al. Effect of in utero and early-life conditions on adult health and disease. *N Engl J Med* 2008;359(1):61-73. doi: 10.1056/NEJMra0708473 [published Online First: 2008/07/04]
3. Heim C, Shugart M, Craighead WE, et al. Neurobiological and psychiatric consequences of child abuse and neglect. *Dev Psychobiol* 2010;52(7):671-90. doi: 10.1002/dev.20494
4. Kingston D, Tough S, Whitfield H. Prenatal and postpartum maternal psychological distress and infant development: a systematic review. *Child Psychiatry Hum Dev* 2012;43(5):683-714.
5. Gelaye B, Rondon M, Araya R, et al. Epidemiology of maternal depression, risk factors, and child outcomes in low-income and middle-income countries. *Lancet Psychiatry* 2016;3(10):973-82. doi: 10.1016/S2215-0366(16)30284-X
6. Davalos DB, Yadon CA, Tregellas HC. Untreated prenatal maternal depression and the potential risks to offspring: a review. *Arch Womens Ment Health* 2012;15(1):1-14.
7. Dong M, Giles WH, Felitti VJ, et al. Insights into causal pathways for ischemic heart disease adverse childhood experiences study. *Circulation* 2004;110(13):1761-66.
8. Dube SR, Fairweather D, Pearson WS, et al. Cumulative childhood stress and autoimmune diseases in adults. *Psychosom Med* 2009;71(2):243.
9. Anda RF, Dong M, Brown DW, et al. The relationship of adverse childhood experiences to a history of premature death of family members. *BMC Public Health* 2009;9(1):1.
10. Chander P, Kvalsvig J, Mellins CA, et al. Intimate Partner Violence and Child Behavioral Problems in South Africa. *Pediatrics* 2017 doi: 10.1542/peds.2016-1059
11. Grantham-McGregor S, Cheung YB, Cueto S, et al. Developmental potential in the first 5 years for children in developing countries. *The Lancet* 2007;369:60-70. doi: 10.1016/S0140-6736(07)60032-4
12. Walker SP, Wachs TD, Meeks Gardner J, et al. Child development: risk factors for adverse outcomes in developing countries. *The Lancet* 2007;369:145-57. doi: 10.1016/S0140-6736(07)60076-2
13. Walker SP, Wachs TD, Grantham-McGregor S, et al. Inequality in early childhood: risk and protective factors for early child development. *The Lancet* 2011;378(9799):1325-38.
14. Luthar SS, Cicchetti D, Becker B. The construct of resilience: A critical evaluation and guidelines for future work. *Child Dev* 2000;71:543-62. doi: 10.1111/1467-8624.00164
15. Ungar M, Ghazinour M, Richter J. Annual Research Review: What is resilience within the social ecology of human development? *J Child Psychol Psychiatry* 2013;54:348-66. doi: 10.1111/jcpp.12025
16. Masten AS. Global perspectives on resilience in children and youth. *Child Dev* 2014;85:6-20. doi: 10.1111/cdev.12205
17. Black RE, Victora CG, Walker SP, et al. Maternal and child undernutrition and overweight in low-income and middle-income countries. *The Lancet* 2013;382:427-51. doi: 10.1016/S0140-6736(13)60937-X
18. Remais JV, Zeng G, Li G, et al. Convergence of non-communicable and infectious diseases in low- and middle-income countries. *Int J Epidemiol*:221-27. doi: 10.1093/ije/dys135
19. Rahman A, Iqbal Z, Harrington R. Life events, social support and depression in childbirth: perspectives from a rural community in the developing world. *Psychol Med* 2003;33 doi: 10.1017/s0033291703008286
20. Zar HJ, Barnett W, Myer L, et al. Investigating the early-life determinants of illness in Africa: the Drakenstein Child Health Study. *Thorax* 2014:thoraxjnl-2014-206242.
21. Stein DJ, Koen N, Donald KA, et al. Investigating the psychosocial determinants of child health in Africa: the Drakenstein Child Health Study. *J Neuroscience methods* 2015;70(6):592-94.

Confidential: For Review Only

22. Zar HJ, Barnett W, Stadler A, et al. Aetiology of childhood pneumonia in a well vaccinated South African birth cohort: a nested case-control study of the Drakenstein Child Health Study. *The Lancet Respiratory Medicine* 2016;4(6):463-72.
23. Shisana O, Rehle T, Simbayi LC, et al. South African national HIV prevalence, incidence and behaviour survey, 2012. 2014
24. Dewing S, Tomlinson M, le Roux IM, et al. Food insecurity and its association with co-occurring postnatal depression, hazardous drinking, and suicidality among women in peri-urban South Africa. *J Affect Disord* 2013;150(2):460-5. doi: 10.1016/j.jad.2013.04.040 [published Online First: 2013/05/28]
25. Hartley M, Tomlinson M, Greco E, et al. Depressed mood in pregnancy: prevalence and correlates in two Cape Town peri-urban settlements. *Reproductive health* 2011;8(1):9.
26. Roos A, Faure S, Lochner C, et al. Predictors of distress and anxiety during pregnancy. *African journal of psychiatry* 2013;16(2):118-22.
27. Petersen Williams P, Jordaan E, Mathews C, et al. Alcohol and other drug use during pregnancy among women attending midwife obstetric units in the Cape Metropole, South Africa. *Advances in preventive medicine* 2014;2014
28. Vythilingum B, Roos A, Faure SC, et al. Risk factors for substance use in pregnant women in South Africa. *SAMJ: South African Medical Journal* 2012;102(11):853-54.
29. Groves AK, Moodley D, McNaughton-Reyes L, et al. Prevalence, rates and correlates of intimate partner violence among South African women during pregnancy and the postpartum period. *Matern Child Health J* 2015;19(3):487-95. doi: 10.1007/s10995-014-1528-6 [published Online First: 2014/06/04]
30. Smit J, Van den Berg C, Bekker L, et al. Translation and cross-cultural adaptation of a mental health battery in an African setting. *African Health Sciences* 2006;6(4)
31. Brittain K, Myer L, Koen N, et al. Risk Factors for Antenatal Depression and Associations with Infant Birth Outcomes: Results From a South African Birth Cohort Study. *Paediatric and perinatal epidemiology* 2015;29(6):505-14.
32. Vanker A, Barnett W, Brittain K, et al. Antenatal and early life tobacco smoke exposure in an African birth cohort study. *The International Journal of Tuberculosis and Lung Disease* 2016;20(6):729-37.
33. Martin L, Revington N, Seedat S. The 39-item Child Exposure to Community Violence (CECV) Scale: Exploratory factor analysis and relationship to PTSD symptomatology in trauma-exposed children and adolescents. *International journal of behavioral medicine* 2013;20(4):599-608.
34. Mejia A, Filus A, Calam R, et al. Measuring parenting practices and family functioning with brief and simple instruments: validation of the Spanish version of the PAFAS. *Child Psychiatry & Human Development* 2015;46(3):426-37.
35. Robinson O, Basagaña X, Agier L, et al. The pregnancy exposome: Multiple environmental exposures in the INMA-Sabadell birth cohort. *Environ Sci Technol* 2015;49:10632-41. doi: 10.1021/acs.est.5b01782

Confidential: For Review Only

Authors' contributions

This study requires multidisciplinary expertise in the execution of measures of this type. DJS is PI of the psychosocial aspects of the DCHS cohort and contributed to the design and decision making involving psychosocial tools and measures used as well as general study design. HJZ is PI of the umbrella DCHS cohort and played a central role in the operational aspects and design of the study. KAD, PI of the child psychosocial aspects of the study, was involved in the design of the study, operational aspects of the study, and played a key role in the child psychosocial measures used. SMS, MH, and CP contributed to decision making involving tools used. NH, WB, and CJW contributed to the operational aspects of the study, QC of data described and data management. RTN contributed to data management. Authors contributed to sections relating to their area of expertise in the manuscript. All authors reviewed and approved the final version of this manuscript.

Confidential: For Review Only

Funding statement

This work was supported by the Bill and Melinda Gates Foundation [OPP 1017641]. Additional support for HJZ and DJS by the MRC of South Africa. Additional aspects of the work reported here are supported by the South African NRF and MRC, by an Academy of Medical Sciences Newton Advanced Fellowship (NAF002\1001), funded by the UK Government's Newton Fund, by NIAAA via (R21AA023887) and by the Collaborative Initiative on Fetal Alcohol Spectrum Disorders (CIFASD) developmental grant (U24 AA014811), by the US Brain and Behaviour Foundation Independent Investigator grant (24467). WB is supported by the SAMRC National Health Scholars programme, and CJW is supported by the Wellcome Trust through a Research Training Fellowship [203525/Z/16/Z].

Confidential: For Review Only

Competing interests statement

The authors declare no competing interests.

Confidential: For Review Only

Acknowledgements

We thank the study staff in Paarl, the study data team and lab teams, the clinical and administrative staff of the Western Cape Government Health Department at Paarl Hospital and at the clinics for support of the study. We acknowledge the advice from members of the study International Advisory Board and thank our collaborators. We thank the families and children who participated in this study.

Confidential: For Review Only

Table 1 Child psychosocial and neurodevelopmental measures

Domain	Measure
**Demographic data**	Household income and assets, maternal education, employment
**Infant health information**	Weight, height, diagnoses
**Infant neurodevelopment**	MRI, DTI, MRS, fMRI
**Antenatal risk factors**	Birth planning; partner support; maternal depression; alcohol, nicotine and illicit substance use; maternal childhood trauma; intimate partner violence; exposure to stressful events; psychological distress; symptoms of peri- and posttraumatic stress
**Trauma and exposure**	
Emotional distress	Paediatric emotional distress scale
Exposure to violence	Child exposure to community violence
	Survey for exposure to community violence
**Parenting**	
Dyadic interaction[a]	Global rating scale
	Emotional availability scale
Parenting practices	Parenting and family adjustment scale
Attachment	Brockington postpartum bonding questionnaire
**Resilience**	Child and youth resilience measure
**General development**	Western Cape screen
	Bayley Scales of Infant and Toddler Development, Third Edition (Bayley-III)
**General cognitive function**	Kaufman assessment battery for children (KABC-II)
Problem solving	KABC-II Conceptual thinking
Visual-spatial memory/processing	KABC-II Face recognition
Visual-spatial processing and problem solving	KABC-II Triangles
Working memory and motor sequencing	KABC-II Hand movements
**Language**	Peabody picture vocabulary test, fourth edition (PPVT-IV)
	KABC-II Expressive vocabulary
**Memory**	
Learning	KABC-II Atlantis
Delayed recall	KABC-II Atlantis delayed
**Executive function**	
Working memory	Wechsler preschool and primary scale of intelligence, fourth edition (WPPSI-IV): Picture memory
Inhibition	Day-night task
Cognitive flexibility	Dimension change card sort
Attention	Test of everyday attention (TEA-Ch): Sky search
**Motor control**	Bayley-III: Fine motor
	Grooved peg board

Confidential: For Review Only

**Social cognition**	
Theory of mind	Diverse desires
	Diverse beliefs
	Understanding intentions
	Perception-leads-to-knowledge
	Location-change false belief
	Unexpected contents false belief
	Belief emotion
	Hidden emotions
Emotion recognition	NEPSY-II: Affect recognition
**Effortful control / emotion regulation**	Snack delay
	Gift-in-bag
	Whisper
	Rydell's emotion questionnaire
**Social competence**	
Affective arousal	Pupil dilation
Attention allocation	Gaze fixation
Empathy	Chicago empathy for pain task
	Questionnaire of cognitive and affective empathy
Prosocial behaviour	Dictator game
Internalising and externalising behaviour	Child behavior checklist
**Temperament/ Personality**	
Temperament	Rothbart infant and early child behavior questionnaires[a]
Callous-unemotional traits	Callous unemotional screening device

Note. References for the measures are given in the appendix. MRI: Magnetic Resonance Imaging, DTI: Diffusion Tensor Imaging, MRS: Magnetic Resonance Spectroscopy, fMRI: functional Magnetic Resonance Imaging
[a]very short forms

Confidential: For Review Only

Table 2 Child sociodemographic characteristics

**Variable**	**Mbekweni**	**TC Newman**	**Total**	***p*-value**
	Number (% of each clinic)			
Mothers	628	509	1137	
Live births	634	509	1143	
Twin sets	4	0	4	
Triplet sets	1	0	1	
Preterm births	107 (16.88)	83 (16.31)	190 (16.32)	0.797
Child's Race				
Black	627 (99.05)	6 (0.95)	633 (55.38)	
Mixed	7 (1.11)	503 (98.82)	510 (44.62)	< 0.001
	Median (IQR)			
Birth weight z-score [a]	-0.4 (-1.2 – 0.2)	-0.7 (-1.4 – -0.1)	-0.5 (-1.3 – 0.1)	< 0.001
Birth length z-score [a]	0.1 (-0.9 – 1.0)	-0.03 (-0.9 – 0.8)	0.003 (-0.9 – 0.9)	0.151

[a] Adjusted for gestation

Confidential: For Review Only

Table 3 Maternal and family sociodemographic characteristics

Variable	Mbekweni	TC Newman	Total	*p*-value
	Number (% of each clinic)			
Maternal educational attainment				0.038
Primary	49 (7.8)	37 (7.3)	86 (7.6)	
Some secondary	340(54.1)	266 (52.3)	606 (53.3)	
Completed secondary	189 (30.1)	183 (36.0)	372 (32.7)	
Any tertiary	50 (8.0)	23 (4.5)	73 (6.4)	
Currently employed	157 (25.0)	149 (29.3)	306 (27.0)	0.106
Married / Cohabiting	237 (37.8)	221 (43.4)	458 (40.3)	0.054
Unplanned pregnancy	366 (68.0)	286 (62.7)	652 (65.6)	0.079
Partner support				< 0.001
Not at all / slightly supportive	42 (7.8)	46 (10.13)	88 (8.9)	
Moderately supportive	86 (16.0)	19 (4.2)	105 (10.6)	
Considerably / extremely supportive	409 (76.1)	389 (85.7)	798 (80.5)	
Reliance on partner for help				< 0.001
Not at all / not very often	53 (9.9)	46 (10.1)	99 (10.0)	
Some of the time	170 (31.7)	28 (6.2)	198 (20.0)	
Most / all of the time	314 (58.5)	380 (83.7)	694 (70.0)	
Monthly income				< 0.001
< R1000 (< $75)	263 (41.9)	167 (32.8)	430 (37.8)	
R1000-R5000 ($75 - $374)	299 (47.6)	254 (49.9)	553 (48.6)	
> R5000 (> $374)	66 (10.5)	88 (17.3)	152 (13.5)	
	Median (IQR)			
Household size	4 (3-6)	5 (4-7)	4 (3-6)	< 0.001
Mother's age at enrolment	27 (22 –32)	25 (21 –29)	26 (22 –31)	< 0.001

Confidential: For Review Only

Table 4 Family risk and protective factors

Variable	Mbekweni	TC Newman	Total	*p*-value
	Mean (SD)			
PAFAS Parenting				
Consistency	7.95 (2.01)	6.24 (2.92)	7.17 (2.61)	< 0.001
Coercive parenting	6.24 (3.38)	5.19 (3.98)	5.76 (3.70)	< 0.001
Encouragement	1.23 (1.66)	1.00 (1.50)	1.13 (1.59)	0.113
Parent-child relationship	0.63 (1.48)	1.37 (1.76)	0.97 (1.65)	< 0.001
PAFAS Family Adjustment				
Parental adjustment	2.72 (2.67)	2.36 (2.86)	2.56 (2.76)	0.025
Family relationships	1.46 (2.01)	2.45 (2.85)	1.91 (2.48)	< 0.001
Parental teamwork	1.32 (1.67)	1.93 (2.08)	1.59 (1.89)	< 0.001
SECV Total	21.27 (6.57)	26.92 (7.46)	23.84 (7.53)	< 0.001
CECV Total	38.65 (3.88)	40.13 (4.21)	38.65 (3.88)	< 0.001

Note. The questionnaires were administered at 2.5 years. Higher scores on the PAFAS indicate higher levels of dysfunction, i.e. higher consistency scores indicate less consistent parenting and higher coercive parenting scores indicate more coercive parenting.
PAFAS: Parenting and Family Adjustment Scale, SECV: Survey for Exposure to Community Violence, CECV: Child Exposure to Community Violence Checklist

Confidential: For Review Only

Figure captions

Figure 1 Time line for child assessment

Confidential: For Review Only

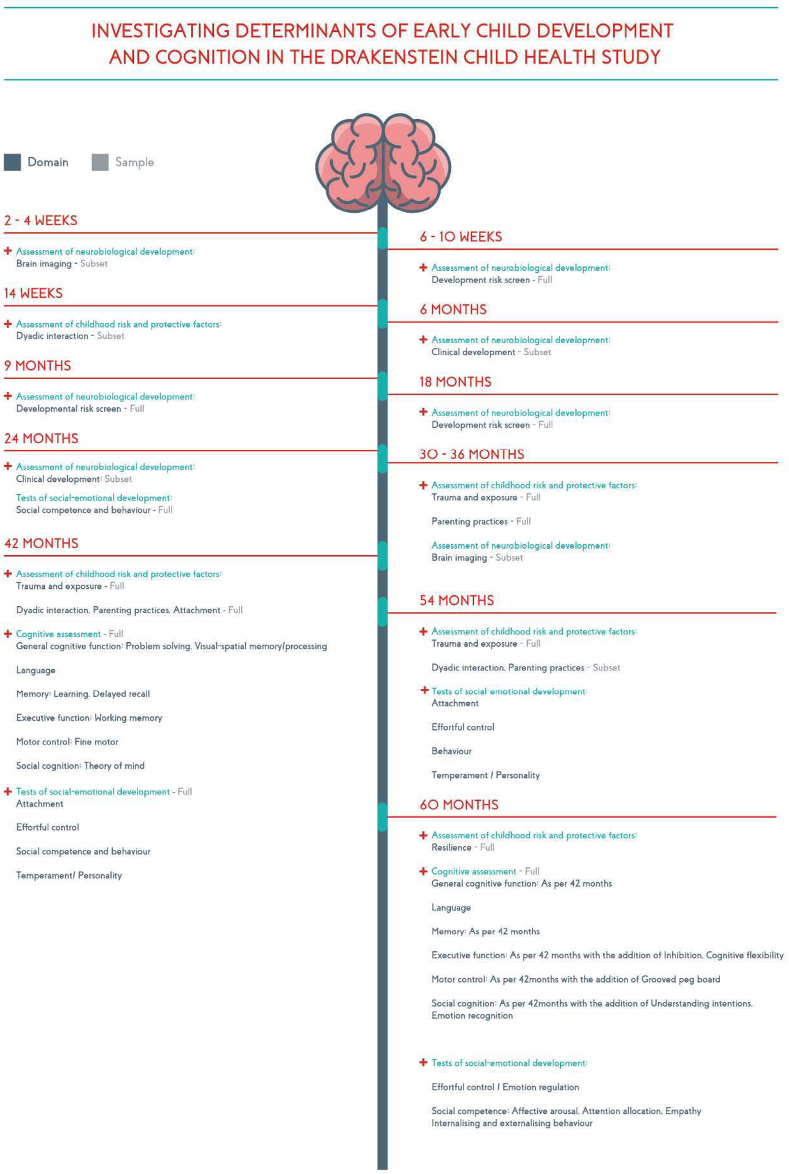
INVESTIGATING DETERMINANTS OF EARLY CHILD DEVELOPMENT AND COGNITION IN THE DRAKENSTEIN CHILD HEALTH STUDY
Domain
Sample
2 - 4 WEEKS
Assessment of neurobiological development:
Brain imaging - Subset
6 - 10 WEEKS
Assessment of neurobiological development:
Development risk screen - Full
14 WEEKS
Assessment of childhood risk and protective factors:
Dyadic interaction - Subset
6 MONTHS
Assessment of neurobiological development:
Clinical development - Subset
9 MONTHS
Assessment of neurobiological development:
Developmental risk screen - Full
18 MONTHS
Assessment of neurobiological development:
Development risk screen - Full
24 MONTHS
Assessment of neurobiological development:
Clinical development: Subset
Tests of social-emotional development:
Social competence and behaviour - Full
30 - 36 MONTHS
Assessment of childhood risk and protective factors:
Trauma and exposure - Full
Parenting practices - Full
Assessment of neurobiological development:
Brain imaging - Subset
42 MONTHS
Assessment of childhood risk and protective factors:
Trauma and exposure - Full
Dyadic interaction, Parenting practices, Attachment - Full
Cognitive assessment - Full
General cognitive function: Problem solving, Visual-spatial memory/processing
Language
Memory: Learning, Delayed recall
Executive function: Working memory
Motor control: Fine motor
Social cognition: Theory of mind
Tests of social-emotional development - Full
Attachment
Effortful control
Social competence and behaviour
Temperament/ Personality
54 MONTHS
Assessment of childhood risk and protective factors:
Trauma and exposure - Full
Dyadic interaction, Parenting practices - Subset
Tests of social-emotional development:
Attachment
Effortful control
Behaviour
Temperament / Personality
60 MONTHS
Assessment of childhood risk and protective factors:
Resilience - Full
Cognitive assessment - Full
General cognitive function: As per 42 months
Language
Memory: As per 42 months
Executive function: As per 42 months with the addition of Inhibition, Cognitive flexibility
Motor control: As per 42months with the addition of Grooved peg board
Social cognition: As per 42months with the addition of Understanding intentions, Emotion recognition
Tests of social-emotional development:
Effortful control / Emotion regulation
Social competence: Affective arousal, Attention allocation, Empathy
Internalising and externalising behaviour

Supplementary Material

Protocol: The Drakenstein Child Health Study (DCHS)

Measures used in the DCHS

These measures aimed to assess overall infant and child development and neurobiological health longitudinally. Broadly, the methods assessed (1) social and biological risk and protective factors, (2) general neurobiological development, (3) cognitive development, and (4) socio-emotional development.

Developmental risk and protective factors

Antenatal risk factors. The parent psychosocial measures are described elsewhere [1]. These included parent reports of whether the birth was planned and partner support was available; maternal depression; alcohol, nicotine and illicit substance use; maternal childhood trauma; intimate partner violence and exposure to stressful events; psychological distress; and symptoms of peri- and posttraumatic stress. The parent psychosocial measures were completed by the mothers during several antenatal and postnatal study visits. Basic demographic data and health information for the infants were obtained from participants' hospital records.

Exposure to traumatic events and violence. The Paediatric Emotional Distress Scale (PEDS) is a brief screening tool for the occurrence of traumatic events during childhood, and is completed by the parent [2]. The PEDs asks about the child's previous experience of a traumatic event and trauma-related experience of anxiety/withdrawal, fearfulness and acting out/ externalising behaviours. Higher scores indicate greater emotional distress.

The Child Exposure to Community Violence Checklist (CECV) is a 33-item parent-report checklist that evaluates children's exposure to violence. The questions contain information on the type and level of violence that the child has witnessed or personally experienced (as victim or perpetrator). Higher scores indicate greater exposure to violence. It has been developed and validated in the South African context. Each of the violence categories have shown good internal consistency in this context [3]. These questionnaires are included because of the strong body of literature on the detrimental effects of childhood exposure to violence on developmental outcomes [4-6].

Confidential: For Review Only

The Survey for Exposure to Community Violence (SECV) is a 12-item checklist that assesses maternal exposure to community violence [7, 8]. As with the CECV, higher scores indicate greater exposure to violence.

Parenting. Parenting is measured through a combination of dyadic interaction observations and maternal self-report of parenting practices and attachment. Dyadic interaction is assessed using the Global Rating Scale (GRS) [9] at 14 weeks and the Emotional Availability Scale (EAS), fourth edition [10], at 3.5 and 4.5 years. Recordings of unstructured maternal-child interactions were done at all three times points. The interactions were recorded during scheduled well-baby visits to Paarl hospital. In the GRS, mothers were instructed to play with their child for 5 minutes without the use of toys. The recordings were scored by raters certified on the GRS, who assessed maternal style (13 items), child temperament (7 items), and the nature of the dyadic interaction (5 items). Infant behaviour items assess attentiveness, active communicative attempts and engagement with the environment. Maternal items assess the mother's responsiveness and sensitivity, directiveness and affect [9, 11]. Interaction items assess the fluidity and emotional valence of the interaction [12].

In the EAS, videotaped unstructured play interactions of 10 minutes in length were coded by trained raters. The EAS includes four parental scales and two child scales; that is, maternal sensitivity, structuring, non-intrusiveness and non-hostility (parental scales), as well as child responsiveness and child involvement [10, 13]. Each scale consists of two subscales rated on a 7-point scale and five subscales rated on a 3-point scale. Higher values signify more desirable behaviour. The EAS has sound psychometric properties internationally and has been used to assess emotional availability from infancy to middle childhood in different social and cultural settings, including South Africa [14, 15]. Previous studies report good inter-rater reliability [15, 16].

The Parenting and Family Adjustment Scale (PAFAS) is a 30-item parent-report measure for assessing parenting practices, parent-child relationship quality, parental emotional adjustment and teamwork, and family relationships [17]. The inventory consists of two scales; parenting and family adjustment. Higher scores indicate greater levels of dysfunction. The PAFAS subscales have shown good internal consistency and adequate construct validity in previous research [17].

Maternal attachment was measured using the self-report Brockington Postpartum Bonding Questionnaire [18]. The questionnaire screens for impaired bonding, rejection, anger, anxiety and incipient abuse. High scores indicate more pathological responses. The questionnaire has shown good test-retest reliability [18] as well as high sensitivity for identifying bonding disorders [19], and has been used in low-SES South African settings [20].

Child resilience was measured using the Child and Youth Resilience Measure (CYRM-28) [21]. This 26-item parent-report questionnaire measures child resilience, with higher scores indicating greater resilience. The questionnaire focusses on seven aspects of resilience; namely, access to material resources, supportive relationships, identity, sense of social justice, feelings of power and control, cohesion, and cultural adherence. It was collaboratively developed in several low-and middle-income countries to enable cross-cultural comparison of developmental outcomes and has satisfactory internal consistency in this context [22].

Neurobiological development

Brain development. A subgroup of children in the Drakenstein Child Health Study cohort underwent multimodal neuroimaging assessment, including those at risk of exposure to alcohol, maternal depression and HIV in pregnancy. The imaging was done at the Cape Universities Brain Imaging Centre (CUBIC) using a Siemens 3 Tesla Skyra machine. The imaging modalities performed included: (1) structural magnetic resonance imaging (MRI) with T1 and T2-weighting to examine cortical and subcortical volumes; (2) diffusion tensor imaging (DTI) for white matter microstructure; (3) magnetic resonance spectroscopy (MRS) and; (4) resting state functional MRI for regional connectivity. The imaging was undertaken at 2-4 weeks of age and again at 2.5-3 years during natural sleep without sedation. Due to the logistical challenges of imaging children at this age, the imaging was conducted at a time of day when the children usually sleep. The imaging has no radiation exposure. The imaging aims to detect anatomical and functional differences associated with antenatal and childhood risk factors.

Western Cape Developmental Screening Questionnaires were administered to the children's caregivers at 6 weeks, 9 months and 18 months of age by primary health care workers during routine immunization visits to the clinic. Each age-specific screening questionnaire (6 weeks, 9 months or 18 months) contains a set of yes/no questions that assess

Confidential: For Review Only

developmental milestones in the domains of hearing, vision, gross motor, fine motor, language and communication, and psychosocial development. The screening questionnaires also assesses caregiver mental health and caregiver-child interaction. The questionnaires form part of the Western Cape Government Department of Health's primary health care screening for moderate and severe neurodevelopmental disabilities and provides a categorical risk outcome score (risk / no risk).

Bayley Scales of Infant and Toddler Development, Third Edition (Bayley-III) [23]. The Bayley-III is a clinician-administered instrument designed to assess developmental functioning between the ages of 1 and 42 months. The instrument monitors children's developmental progress in the areas of cognition, motor skills and language, as well as socio-emotional and adaptive functioning [24]. Each subscale is scored according to age-appropriate norms. A cut-off of 7 or less in any subscale scaled score is a difference of 1 standard deviation from the mean and indicates suboptimal development in that domain [23]. The Bayley-III was administered on a subset of infants at ages 6 months and the whole cohort at 2 years in the DCHS in order to assess childhood development over time. It has been used globally, including in LMIC settings such as South Africa [25].

Cognitive development

General cognitive function. A measure of general cognitive function was obtained through the Kaufman Assessment Battery for Children, second edition (KABC-II; Pearson Assessments). The KABC-II Mental Processing scale is utilised due to the diverse cultural and language demographics within the Drakenstein communities. The KABC-II was primarily designed to be a culture-fair tool used to assess cognitive function in children aged 3.0 – 18.11 years old, minority groups and children with learning disabilities [26]. In specific, the following subtests of the KABC-II were utilised:

i. Conceptual Thinking (problem solving)
ii. Face Recognition (visual-spatial processing)
iii. Triangles (visual-spatial processing and problem solving)
iv. Hand Movements (working memory and motor sequencing)

Confidential: For Review Only

The Mental Processing scale of the KABC-II is reported to have excellent internal and test-retest reliability, as well as good convergent validity [27]. The KABC-II has been used in a variety of settings in Africa and South Africa [28-32].

Language. The Peabody Picture Vocabulary Test, fourth edition (PPVT-IV) [33] and KABC-II Expressive Vocabulary [34] subtests were used to assess language. The KABC-II Expressive Vocabulary task measures the ability to verbally identify a set of pictures. The PPVT-4 measures receptive vocabulary and is designed for use in a wide age range (ages 2.5 to 90 years). The examiner reads out a word, and then the child responds by pointing to the picture they think corresponds to the word the examiner has given. The test is untimed, but takes approximately 10 – 15 minutes. The PPVT-4 has excellent test-retest reliability and internal consistency [33]. The PPVT-4 has been translated into both Afrikaans and isiXhosa [35].

Memory. The KABC-II Atlantis tasks [36] were used to assess learning and recall. The Atlantis task assesses the ability to learn and remember a series of nonsense words paired with pictures. The child is instructed to point to the correct picture when a word is read. The task is repeated after a 15 – 20-minute delay.

Executive function. Working memory was assessed using the Picture Memory task from the Wechsler Preschool and Primary Scale of Intelligence, fourth edition (WPPSI-IV) [37]. The WPPSI-IV was designed for children aged 2.6 – 7.7 years old. The Picture Memory subtask uses the familiarise-recognise paradigm, wherein one or more stimuli are first viewed and then recognised from among an item set. Picture Memory has good test-retest reliability and excellent internal consistency in US samples in both the 2:5 – 3:11 and 4:0 – 7:7 age bands [38]. Previous versions of the WPPSI have been used successfully in the Western Cape region to assess cognitive functioning [39, 40].

Inhibition was assessed using the Stroop-like Day-Night task [41]. The task tests the ability to inhibit a prepotent response by requiring the child to say the opposite of what is shown on a set of cards. Two types of card are available in the deck: Children are instructed to say “day” when presented with a black moon card (night) card, and “night” when presented with a white sun card (day). The number of correct trials are scored. Studies of Day-Night task suggest that it has good internal consistency [42] and adequate test-retest reliability [43, 44].

Cognitive flexibility was assessed using the Dimensional Change Card Sort (DCSS) [45, 46], which resembles the Wisconsin Card Sorting Test (WCST). Participants are required to

Confidential: For Review Only

sort a series of cards, which contain two salient features/dimensions (e.g., colour and shape), first according to one dimension and then according to the other. The DCCS was chosen as it is appropriate for a wide age range and has excellent convergent validity and test-retest reliability [47, 48].

Selective attention was assessed using the Sky Search subtest from the Test of Everyday Attention for Children (TEA-Ch) [49]. The child is instructed to find all the identical pairs on an A3 sheet with spacecraft. Completion time and number of errors are recorded. Sky Search has excellent test-retest reliability [49].

Motor control is assessed using the Fine Motor subtest of the Bayley-III (described above) [23] and the Grooved Pegboard (Lafayette Instrument Company, Inc.). The Bayley-III Fine Motor assesses visual tracking, reaching, perceptual-motor integration, and motor planning and speed. The task has good internal consistency as well as adequate test-retest reliability and convergent validity [23, 50]. The Grooved Pegboard (Lafayette Instrument Company, Inc.) tests manipulative dexterity in the dominant and non-dominant hands. The task has 25 grooved holes arranged in rows. The participant must rotate pegs to insert them into the holes. The total completion time (in seconds) for each trial is recorded. The Grooved Pegboard test is reported to show excellent test-retest reliability for both hands and relatively strong concurrent validity in a 3-9 year old sample [51]. The Grooved Pegboard has been used successfully in South African samples [52].

Social cognition. Theory of Mind (ToM) was examined via subtasks from the early and basic modules of the UCT Theory of Mind battery [39, 53]. The early ToM module contains the Desires/Understanding Intentions and Perception-Leads-to-Knowledge tasks, as well as the Diverse Desires and Diverse Belief tasks [54]. The Diverse Desires task assesses the ability to understand that a character can have desires different from one's own, and that these desires will influence the choices they make. Similarly, the Diverse Beliefs assesses the ability to understand that a character can have beliefs different from one's own, and that these beliefs will influence a character's actions. The basic ToM module contains two tasks that assess false belief, namely the Location-Change False Belief and Unexpected Contents False Belief tasks, as well as the Belief Emotion and Hidden Emotions (Real/Apparent Emotion) tasks [54]. The false belief tasks assess whether the child knows that a character can hold a belief that is not true, and that their actions will be based on their beliefs rather than the reality of the situation. Belief Emotion assesses whether the child can infer how a character will feel, given that the character's belief is mistaken. Hidden Emotion assesses whether the

Confidential: For Review Only

child knows that a character can outwardly display a different emotion from what they are feeling. At age 3.5, only Diverse Desires was administered; at age 4.5, Diverse Desires and Diverse Beliefs was administered. At age 5, the remaining tasks from the early module, as well as all the tasks from the basic module, were administered. The tasks have been adapted and used successfully locally with low SES participants.

Facial emotion recognition was assessed using the NEPSY-II Affect recognition task [55]. The stimuli feature a set of photographs of children from various race groups displaying a specific emotion. The participant is required to match the emotion to photographs of children displaying the same affect. As the task requires matching rather than labelling, it is less reliant on language. The task has been used successfully in local research with low SES participants [53].

Socio-emotional development

The socio-emotional assessment included measures of emotion regulation, affective arousal and social attention-allocation, empathy, morality, prosocial behaviour, temperament and callous-unemotional traits.

Emotion regulation and effortful control were captured through both observational and parent-report measures. Effortful control was observationally assessed with the Kochanska tasks [56], which have been designed for this purpose. The tasks assess delay of gratification, emotion regulation, and motor inhibition abilities. At age 3.5 and 4.5 years, variants of the Snack Delay task were used; at age 5, both the Snack Delay and Gift-in-Bag are used, alongside the Whisper task. The Snack Delay and Gift-in-Bag tasks assess the effortful control ability of delaying an action, while the Whisper task assesses voice lowering ability. In the variant of the Snack Delay task used at age 3.5, the researcher and child each place a smartie (a small sweet) on their tongues and compete to see who can refrain from eating it the longest. Additionally, the child is asked to hold a smartie on their tongue for four trials of 10, 20, 30, and 15s, respectively. The number of successful delays are coded. In the variant of the Snack Delay task used at 4.5 years, the child is given an unwrapped chocolate and told that if they do not eat it for the duration of the interview with the caregiver (15 minutes), they will be given two additional chocolates at the end. The time until child eats the chocolate is scored. In the Gift-in-Bag task, a colourfully-wrapped gift is placed in front of the child. The child is asked to stay seated not to touch the gift and the experimenter leaves the room for 3 minutes. The child is given scores for staying seated and for his or her

Confidential: For Review Only

behaviour with the gift, where higher scores indicate greater behavioural control. In the Whisper task, the experimenter shows the child a series of pictures of popular cartoon characters and asks him or her to whisper the name of each character. The child's ability to modulate their voice is recorded. These tasks were chosen because they are simple to perform and rely very little on language. From 33 months of age, the effortful control tasks show adequate consistency across tasks and have good longitudinal stability in a US sample [56, 57].

Parent-report of emotion regulation was captured using Rydell's Emotion Questionnaire [58]. This 16-item short form measures the child's typical reaction to and regulation of sadness, anger, fear and exuberance. Scale totals are calculated by taking the mean of the item scores. Depending on the scale, high scores indicate either high levels of emotionality or high levels of emotion regulation ability. Good construct validity and test-retest reliability has been reported previously [58].

Attentional-allocation and affective arousal to visual emotional stimuli was assessed in a subgroup of children using eye gaze and pupil dilation measures. The eye tracking assessments were conducted in a research laboratory using a remote Tobii Studio X60 eye tracker (Tobii Technology, 2010)(60 Hz acquisition rate). Participants are shown pictures of infant faces with either positive (smiling), negative (crying) or neutral expressions [59]. The stimuli feature Black, White and Mixed Race faces. The timing, duration and location of fixations are determined for each stimulus, and are used to index attentional-allocation. Pupil dilation during the stimulus is used as an index of autonomic arousal, and has been shown to correlate strongly with sympathetic arousal in particular [60]. The use of eye tracking is particularly well-suited to child studies, as it does not require advanced language or motor responses, and is a non-invasive and objective measure of arousal and attention [61, 62]. In addition, visual stimuli of facial expressions depicting various emotions is frequently used to induce arousal and measure attention-allocation [63, 64].

Maternal and child empathy. Self-reported (child) empathy was assessed via the Chicago empathy for pain task [65]. The task features everyday situations depicting either a painful or a neutral (non-painful) event, and is suitable for children aged 3 years and up. To assess affective empathy, participants are asked to rate how much pain they think the character is experiencing on a visual analogue scale. To assess empathic concern, participants rate how sorry they the feel for the character. Participant responses are coded as 1 – 100, where 100 is the maximum possible pain/ concern. Empathic concern and affective empathy scores are calculated as the mean response over all trials (36 trials; 18 pain and 18 non-pain).

Confidential: For Review Only

Maternal empathy and child dispositional empathy were assessed with the Questionnaire of Cognitive and Affective Empathy (QCAE) [66]. The QCAE is completed by the mother/caregiver and contains 31 questions assessing either cognitive or affective empathy. Mothers/caregivers rate the degree to which each item describes them (maternal empathy) and their child (child empathy); higher scores indicate greater empathy. The subscales of the QCAE have shown adequate to good internal consistency in low SES samples in South Africa [67, 68]. Maternal dispositional empathy impacts on the dyadic mother-child relationship, and is thought to have some influence on the child's developing empathy. This in turn impacts on the child's social interactions overall, and hence on the developing socioemotional and social cognitive skill set.

Prosocial behaviour was assessed using a simple sharing task, a variation on the Dictator Game. The child chooses a set of desirable items (attractive stickers), and is asked if they would like to share with their siblings or friends. The child is instructed to place the items to be given away in a separate container. To avoid social-desirability bias, the administrator turns his/her back while the child does the sharing. Prosocial behaviour is coded as the number of items shared. This task has been used successfully in local research [67].

Internalising and externalising behaviour was assessed with the parent-report version of the Child Behaviour Checklist (CBCL) [69]. The measure assesses child social competence in the school, social and activities domains, and the presence of internalising and externalising behaviours in the last 6 months. High scores on the CBCL that indicate the presence of problem behaviours and inadequate competencies in relation to age and gender-matched peers. The measure is widely used internationally, and has proved reliable in South Africa [70, 71].

Temperament was assessed via short forms of the widely-used Rothbart questionnaires. The Rothbart Infant Behaviour Questionnaire-Revised, very short form, is used to acquire retrospective report on infant temperament at 3.5 years. The Rothbart Early Child Behaviour Questionnaire, very short form [72], was used to measure current temperament at 4.5 years [73]. The questionnaires measure negative emotionality, positive affect, and orienting/regulatory capacity. The very short forms have shown adequate internal consistency and high correlations with the standard forms [72, 73], and have been used in low-resource African settings [74].

Confidential: For Review Only

Presence of callous-unemotional traits was assessed using the Callous Unemotional Screening Device, a 9-item parent-report measure based on Dadds et al. [75]. The screening device combines items related to callous-unemotional traits from the Strengths and Difficulties Questionnaire (SDQ) [76, 77] and the Antisocial Process Screening Device (APSD) [78]. The measure has demonstrated adequate internal consistency in local research with low SES participants [71]. It was felt to be important to include a measure of callous-unemotional personality traits in this cohort, as these traits are influenced by early exposure to adversity and the nature of the child's primary relationships and predict risk for psychopathology in adulthood [79, 80].

References

1. Stein D, Koen N, Donald K, et al. Investigating the psychosocial determinants of child health in Africa: The Drakenstein Child Health Study. *J Neurosci Methods* 2015; **252**: 27-35.
2. Saylor CF, Swenson CC, Stokes Reynolds S, Taylor M. The pediatric emotional distress scale: A brief screening measure for young children exposed to traumatic events. *J Clin Child Psychol* 1999; **28**: 70-81.
3. Martin L, Revington N, Seedat S. The 39-Item Child Exposure to Community Violence (CECV) Scale: Exploratory factor analysis and relationship to PTSD symptomatology in trauma-exposed children and adolescents. *Int J Behav Med* 2013; **20**: 599-608.
4. Engle PL, Fernald LC, Alderman H, et al. Strategies for reducing inequalities and improving developmental outcomes for young children in low-income and middle-income countries. *The Lancet* 2011; **378**: 1339-53.
5. Walker SP, Wachs TD, Grantham-McGregor S, et al. Inequality in early childhood: risk and protective factors for early child development. *The Lancet* 2011; **378**: 1325-38.
6. Walker SP, Wachs TD, Meeks Gardner J, et al. Child development: risk factors for adverse outcomes in developing countries. *The Lancet* 2007; **369**: 145-57.
7. DeCou CR, Lynch SM. Assessing adult exposure to community violence: A review of definitions and measures. *Trauma Violence Abuse* 2017; **18**: 51-61.
8. Richters JE, Saltzman W. *Survey of exposure to community violence: Self-report version*: JE Richters; 1990.
9. Murray L, Fiori-Cowley A, Hooper R, Cooper P. The impact of postnatal depression and associated adversity on early mother-infant interactions and later infant outcome. *Child Dev* 1996; **67**: 2512-26.
10. Biringen Z. *The emotional availability (EA) scales*. Fourth ed. Boulder, CO; 2008.
11. Humber N, Moss E. The relationship of preschool and early school age attachment to mother-child interaction. *Am J Orthopsychiatry* 2005; **75**: 128-41.
12. Kim S, Kochanska G. Child temperament moderates effects of parent–child mutuality on self-regulation: A relationship-based path for emotionally negative infants. *Child Dev* 2012; **83**: 1275-89.
13. Biringen Z. Emotional availability: Conceptualization and research findings. *Am J Orthopsychiatry* 2000; **70**: 104-14.
14. Bain K. "New beginnings" in South African shelters for the homeless: Piloting of a group psychotherapy intervention for high-risk mother-infant dyads. *Infant Ment Health J* 2014; **35**: 591-603.
15. Gul H, Erol N, Pamir A, et al. Emotional availability in early mother-child interactions for children with autism spectrum disorders, other psychiatric disorders, and developmental delay. *Infant Ment Health J* 2016; **37**: 151-9.

Confidential: For Review Only

16. van Ee E, Kleber RJ, Mooren TTM. War trauma lingers on: Associations between maternal posttraumatic stress disorder, parent-child interaction, and child development. *Infant Ment Health J* 2012; **33**: 459-68.
17. Sanders MR, Morawska A, Haslam DM, Filus A, Fletcher R. Parenting and Family Adjustment Scales (PAFAS): Validation of a brief parent-report measure for use in assessment of parenting skills and family relationships. *Child Psychiatry Hum Dev* 2014; **45**: 255-72.
18. Brockington IF, Oates J, George S, et al. A Screening Questionnaire for mother-infant bonding disorders. *Arch Womens Ment Health* 2001; **3**: 133-40.
19. Brockington IF, Fraser C, Wilson D. The Postpartum Bonding Questionnaire: a validation. *Arch Womens Ment Health* 2006; **9**: 233-42.
20. Rotheram-Borus MJ, Richter LM, Heerden Av, et al. A cluster randomized controlled trial evaluating the efficacy of peer mentors to support South African women living with HIV and their infants. *PLoS One* 2014; **9**: e84867.
21. Ungar M, Liebenberg L. Assessing resilience across cultures using mixed methods: Construction of the child and youth resilience measure. *J Mix Methods Res* 2011; **5**: 126-49.
22. Liebenberg L, Ungar M, Vijver FVd. Validation of the Child and Youth Resilience Measure-28 (CYRM-28) among Canadian youth. *Res Soc Work Pract* 2012; **22**: 219-26.
23. Bayley N. *Bayley Scales of Infant and Toddler Development*. Third ed. San Antonio, TX: Psychological Corporation; 2006.
24. Albers CA, Grieve AJ. Review of Bayley Scales of Infant and Toddler Development--Third Edition. *J Psychoeduc Assess* 2007; **25**: 180-90.
25. Ballot DE, Potterton J, Chirwa T, Hilburn N, Cooper PA. Developmental outcome of very low birth weight infants in a developing country. *BMC Pediatr* 2012; **12**: 11.
26. Lichtenberger EO, Sotelo-Dynega M. The Kaufman Assessment Battery for Children—Second Edition. *Practitioner's Guide to Assessing Intelligence and Achievement* 2009: 61.
27. Kaufman AS, Lichtenberger EO, Fletcher-Janzen E, Kaufman NL. *Essentials of KABC-II assessment*. Hoboken, NJ: John Wiley & Sons; 2005.
28. Baumgartner J, Smuts CM, Malan L, et al. Effects of iron and n−3 fatty acid supplementation, alone and in combination, on cognition in school children: a randomized, double-blind, placebo-controlled intervention in South Africa. *Am J Clin Nutr* 2012; **96**: 1327-38.
29. Boivin MJ, Vokhiwa M, Sikorskii A, Magen JG, Beare N. Cerebral malaria retinopathy predictors of persisting neurocognitive outcomes in Malawian children. *Pediatr Infect Dis J* 2014.
30. Greenop K, Fry J, de Sousa D. The Kaufman Assesment Battery in South Africa. In: Laher SC, K., editor. *Psychological assessment in South Africa: Research and Applications*. Johannesburg: Wits University Press; 2013. p. 86-103.
31. Ruel TD, Boivin MJ, Boal HE, et al. Neurocognitive and motor deficits in HIV-infected Ugandan children with high CD4 cell counts. *Clin Infect Dis* 2012; **54**: 1001-9.
32. Taljaard C, Covic NM, Van Graan AE, et al. Effects of a multi-micronutrient-fortified beverage, with and without sugar, on growth and cognition in South African schoolchildren: a randomised, double-blind, controlled intervention. *Br J Nutr* 2013; **110**: 2271-84.
33. Dunn LM, Dunn DM. *PPVT-4: Peabody Picture Vocabulary Test*. Minneapolis, MN: Pearson Assessments; 2007.
34. Kaufman AS, Kaufman NL. *KABC-II: Kaufman Assessment Battery for Children*. Circle Pines, MN: AGS Pub.; 2004.
35. Dawes A, Biersteker L, Hendricks L. *Towards integrated early childhood development. An evaluation of the Sobambisana Initiative*. ilifa labantwana 2012 [cited 2017 12 August]; Available from: http://ilifalabantwana.co.za/an-evaluation-of-the-sobambisana-initiative
36. Kaufman AS, Kaufman NL. Kaufman Assessment Battery for Children, Second Edition. *Encyclopedia of Special Education*. Hoboken, NJ: John Wiley & Sons, Inc.; 2013.
37. Wechsler D. *WPPSI-IV: Wechsler Preschool and Primary Scale of Intelligence: Manual*. London, United Kingdom: Pearson Education; 2012.
38. Williams ME, Sando L, Soles TG. Cognitive tests in early childhood: Psychometric and cultural considerations. *J Psychoeduc Assess* 2014; **32**: 455-76.
39. Hoogenhout M, Malcolm-Smith S. Theory of mind in autism spectrum disorder: Does DSM classification predict development? *Res Autism Spectr Disord* 2014; **8**: 597-607.

Confidential: For Review Only

40. Pileggi L-A, Malcolm-Smith S, Solms M. Investigating the role of social-affective attachment processes in cradling bias: The absence of cradling bias in children with Autism Spectrum Disorders. *Laterality* 2015; **20**: 154-70.
41. Gerstadt CL, Hong YJ, Diamond A. The relationship between cognition and action: performance of children 312–7 years old on a Stroop- like day-night test. *Cognition* 1994; **53**: 129-53.
42. Rhoades BL, Greenberg MT, Domitrovich CE. The contribution of inhibitory control to preschoolers' social–emotional competence. *J Appl Dev Psychol* 2009; **30**: 310-20.
43. Hughes C, Ensor R. Does executive function matter for preschoolers' problem behaviors? *J Abnorm Child Psychol* 2008; **36**: 1-14.
44. Thorell LB, Wåhlstedt C. Executive functioning deficits in relation to symptoms of ADHD and/or ODD in preschool children. *Infant Child Dev* 2006; **15**: 503-18.
45. Frye D, Zelazo PD, Palfai T. Theory of mind and rule-based reasoning. *Cogn Dev* 1995; **10**: 483-527.
46. Zelazo PD. The Dimensional Change Card Sort (DCCS): A method of assessing executive function in children. *Nat Protoc* 2006; **1**: 297-301.
47. Beck DM, Schaefer C, Pang K, Carlson SM. Executive function in preschool children: Test–retest reliability. *J Cogn Dev* 2011; **12**: 169-93.
48. Zelazo PD, Bauer PJ, Fox NA. *National Institutes of Health Toolbox Cognition Battery (NIH Toolbox CB): Validation for children between 3 and 15 years*: Wiley; 2013.
49. Manly T, Anderson V, Nimmo-Smith I, Turner A, Watson P, Robertson IH. The differential assessment of children's attention: The Test of Everyday Attention for Children (TEA-Ch), normative sample and ADHD performance. *J Child Psychol Psychiatry* 2001; **42**: 1065-81.
50. Folio MR, Fewell RR. *Peabody developmental motor scales: Examiner's manual*: Pro-ed; 2000.
51. Wang Y-C, Magasi SR, Bohannon RW, et al. Assessing dexterity function: a comparison of two alternatives for the NIH Toolbox. *J Hand Ther* 2011; **24**: 313-21.
52. Meyer A, Sagvolden T. Fine motor skills in South African children with symptoms of ADHD: influence of subtype, gender, age, and hand dominance. 2006; **2**: 33.
53. Lindinger NM, Malcolm-Smith S, Dodge NC, et al. Theory of mind in children with fetal alcohol spectrum disorders. *Alcohol Clin Exp Res* 2016; **40**: 367-76.
54. Wellman HM, Liu D. Scaling of theory□of□mind tasks. *Child Dev* 2004; **75**: 523-41.
55. Korkman M, Kirk U, Kemp S. *NEPSY II Clinical & Interpretative Manual*. San Antonio, Texas: The Psychological Corporation; 2007.
56. Kochanska G, Murray KT, Harlan ET. Effortful control in early childhood: Continuity and change, antecedents, and implications for social development. *Dev Psychol* 2000; **36**: 220-32.
57. Murray KT, Kochanska G. Effortful control: Factor structure and relation to externalizing and internalizing behaviors. *J Abnorm Child Psychol* 2002; **30**: 503-14.
58. Rydell A-M, Berlin L, Bohlin G. Emotionality, emotion regulation, and adaptation among 5- to 8-year-old children. *Emotion* 2003; **3**: 30-47.
59. Yrttiaho S, Niehaus D, Thomas E, Leppänen JM. Mothers' pupillary responses to infant facial expressions. *Behav Brain Func* 2017; **13**: 2.

60. Bradley MM, Miccoli L, Escrig MA, Lang PJ. The pupil as a measure of emotional arousal and autonomic activation. *Psychophysiology* 2008; **45**: 602-7.
61. Jackson I, Sirois S. Infant cognition: going full factorial with pupil dilation. *Dev Sci* 2009; **12**: 670-9.
62. Laeng B, Sirois S, Gredebäck G. Pupillometry: A window to the preconscious? *Perspect Psychol Sci* 2012; **7**: 18-27.
63. Kylliäinen A, Hietanen JK. Skin conductance responses to another person's gaze in children with autism. *J Autism Dev Disord* 2006; **36**: 517-25.
64. Kylliäinen A, Wallace S, Coutanche MN, et al. Affective–motivational brain responses to direct gaze in children with autism spectrum disorder. *J Child Psychol Psychiatry* 2012; **53**: 790-7.
65. Decety J, Michalska KJ, Akitsuki Y. Who caused the pain? An fMRI investigation of empathy and intentionality in children. *Neuropsychologia* 2008; **46**: 2607-14.

Confidential: For Review Only

66. Reniers RL, Corcoran R, Drake R, Shryane NM, Völlm BA. The QCAE: A questionnaire of cognitive and affective empathy. *J Pers Assess* 2011; **93**: 84-95.
67. Decety J, Cowell Jason M, Lee K, et al. The negative association between religiousness and children's altruism across the world. *Curr Biol* 2015; **25**: 2951-5.
68. Nefdt K. *Empathy across socioeconomic status and its association with aggressive behaviour in Western Cape children* [Unpublished honours thesis]. Cape Town, South Africa: University of Cape Town; 2013.
69. Achenbach TM, Rescorla LA. *Manual for the ASEBA Preschool Forms & Profiles*. Burlington, Vermont: University of Vermont, Research Center for Children, Youth & Families; 2001.
70. Barbarin OA, Richter L, deWet T. Exposure to violence, coping resources, and psychological adjustment of South African children. *Am J Orthopsychiatry* 2001; **71**: 16-25.
71. Malcolm-Smith S, Woolley D, Ward CL. Examining empathy and its association with aggression in young Western Cape children. *J Child Adolesc Ment Health* 2015; **27**: 135-47.
72. Putnam SP, Helbig AL, Gartstein MA, Rothbart MK, Leerkes E. Development and assessment of Short and Very Short Forms of the Infant Behavior Questionnaire–Revised. *J Pers Assess* 2014; **96**: 445-58.
73. Putnam SP, Rothbart MK. Development of Short and Very Short Forms of the Children's Behavior Questionnaire. *J Pers Assess* 2006; **87**: 102-12.
74. Gartstein MA, Bogale W, Meehan CL. Adaptation of the Infant Behavior Questionnaire-Revised for use in Ethiopia: Expanding cross-cultural investigation of temperament development. *Infant Behav Dev* 2016; **45, Part A**: 51-63.
75. Dadds MR, Fraser J, Frost A, Hawes DJ. Disentangling the underlying dimensions of psychopathy and conduct problems in childhood: a community study. *J Consult Clin Psychol* 2005; **73**: 400-10.
76. Goodman R. The strengths and difficulties questionnaire: A research note. *J Child Psychol Psychiatry* 1997; **38**: 581-6.
77. Goodman R, Meltzer H, Bailey V. The strengths and difficulties questionnaire: A pilot study on the validity of the self-report version. *Eur Child Adolesc Psychiatry* 1998; **7**: 125-30.
78. Frick PJ, Hare RD. *Antisocial process screening device: APSD.* Toronto: Multi-Health Systems; 2001.
79. Frick PJ, White SF. Research Review: The importance of callous-unemotional traits for developmental models of aggressive and antisocial behavior. *J Child Psychol Psychiatry* 2008; **49**: 359-75.
80. Viding E, Price TS, Jaffee SR, et al. Genetics of callous-unemotional behavior in children. *PLoS One* 2013; **8**: e65789.

Confidential: For Review Only

Journal of Neuroscience Methods 252 (2015) 27–35

Contents lists available at ScienceDirect

Journal of Neuroscience Methods

journal homepage: www.elsevier.com/locate/jneumeth

Clinical Neuroscience

Investigating the psychosocial determinants of child health in Africa: The Drakenstein Child Health Study

D.J. Stein[a,b,*], N. Koen[a,b], K.A. Donald[c], C.M. Adnams[a], S. Koopowitz[a], C. Lund[a], A. Marais[a], B. Myers[a,e], A. Roos[b,d], K. Sorsdahl[a], M. Stern[a], M. Tomlinson[f], C. van der Westhuizen[a], B. Vythilingum[a], L. Myer[g], W. Barnett[c], K. Brittain[c], H.J. Zar[c]

[a] Department of Psychiatry and Mental Health, University of Cape Town, South Africa
[b] Medical Research Council (MRC), Unit on Anxiety & Stress Disorders, South Africa
[c] Department of Paediatrics and Child Health, Red Cross War Memorial Children's Hospital and Medical Research Council Unit on Child & Adolescent Health, University of Cape Town, South Africa
[d] Department of Psychiatry, Stellenbosch University, South Africa
[e] Alcohol, Tobacco and Other Drug Research Unit, South African Medical Research Council, South Africa
[f] Department of Psychology, Stellenbosch University, South Africa
[g] School of Public Health and Family Medicine, University of Cape Town, South Africa

HIGHLIGHTS

- The Drakenstein Child Health Study (DCHS) is a multidisciplinary birth cohort in South Africa.
- We review the psychosocial measures used in the DCHS and provide initial data.
- We found a high prevalence of antenatal depression, substance use, and intimate partner violence.
- Perinatal psychosocial risk factors may affect child neurodevelopmental and health outcomes.
- Longitudinal assessment may clarify the underlying mechanisms which impact on child health.

ARTICLE INFO

Article history:
Received 13 October 2014
Received in revised form 11 March 2015
Accepted 12 March 2015
Available online 19 March 2015

Keywords:
Birth cohort
Child health
Depression
Substance use
Intimate partner violence
South Africa

ABSTRACT

Background: Early life psychobiological and psychosocial factors play a key role in influencing child health outcomes. Longitudinal studies may help elucidate the relevant risk and resilience profiles, and the underlying mechanisms that impact on child health, but there is a paucity of birth cohort data from low and middle-income countries (LMIC). We describe the rationale for and present baseline findings from the psychosocial component of the Drakenstein Child Health Study (DCHS).

Methods: We review the psychosocial measures used in the DCHS, a multidisciplinary birth cohort study in a peri-urban area in South Africa, and provide initial data on psychological distress, depression, substance use, and exposure to traumatic stressors and intimate partner violence (IPV). These and other measures will be assessed longitudinally in mothers in order to investigate associations with child neurodevelopmental and health outcomes.

Results: Baseline psychosocial data is presented for mothers ($n = 634$) and fathers ($n = 75$) who have completed antenatal assessments to date. The sample of pregnant mothers is characterized by multiple psychosocial risk factors, including a high prevalence of psychological distress and depression, high levels of substance use, and high exposure to traumatic stressors and IPV.

Discussion: These data are consistent with prior South African studies which have documented a high prevalence of a multitude of risk factors during pregnancy. Further longitudinal assessment of mothers and children may clarify the underlying psychobiological and psychosocial mechanisms which impact on child health, and so inform clinical and public health interventions appropriate to the South African and other LMIC contexts.

© 2015 Elsevier B.V. All rights reserved.

* Corresponding author at: Department of Psychiatry, Groote Schuur Hospital J2, Anzio Road, Observatory 7925, Cape Town, South Africa. Tel.: +27 21 406 6566.
E-mail address: dan.stein@uct.ac.za (D.J. Stein).

http://dx.doi.org/10.1016/j.jneumeth.2015.03.016
0165-0270/© 2015 Elsevier B.V. All rights reserved.

Confidential: For Review Only

1. Background

There is growing evidence that early life events have a profound impact on later health outcomes (Gluckman et al., 2008; Heim et al., 2010). The first few years of life constitute a critical period, during which psychobiological and psychosocial factors may influence not only developmental outcomes, but also lifelong health trajectories (Anda et al., 2006; Maggi et al., 2010). Examples of psychosocial risk factors include low socioeconomic status (SES) during childhood; psychological distress and exposure to stressors during pregnancy and the postnatal period; and an adverse early family environment (Taylor, 2010; Kingston et al., 2012). The underlying mechanisms which account for these associations are also increasingly understood (Gluckman et al., 2008; Heim et al., 2010).

Early psychobiological and psychosocial risk factors may impact a range of organs, including the nervous and respiratory systems, and parental psychological factors may influence both biological and psychological infant outcomes. Maternal prenatal anxiety, for example, has been associated with infant respiratory illness (Beijers et al., 2010), while perinatal depression has been associated with infant lower respiratory tract infections (LRTI) (Ban et al., 2010). Use of nicotine (Hollams et al., 2014; Stocks et al., 2013) and alcohol (Stocks et al., 2013) during pregnancy have been associated with both reduced lung function and detrimental neurodevelopmental outcomes, with the underlying mechanisms being partially elucidated. Associations have also been found between intimate partner violence (IPV) and child or adolescent behavioral problems (Carter et al., 2010; Flach et al., 2011).

The majority of such work has, however, taken place in high-income countries, where only a relatively small percentage of the world's population lives. Psychobiological and psychosocial risk factor profiles in low and middle-income countries (LMICs) differ from those of developed countries. In LMICs, maternal depression and exposure to violence may be more prevalent, and there is a higher prevalence of low birthweight, childhood under-nutrition, and infectious disease, and such differences may be associated with lifetime health trajectories (Walker et al., 2007, 2011). Still, there is a paucity of data from LMICs, and much remains unknown about risk and protective factors within these contexts – particularly about how multiple risk factors intersect to impact on developmental and health outcomes (Lund, 2014).

Given the paucity of longitudinal birth cohort data in LMICs, we have undertaken the Drakenstein Child Health Study (DCHS), a multidisciplinary early life study investigating the determinants of child health, including childhood respiratory function and infant neurodevelopmental outcomes, in two peri-urban communities in the Western Cape Province of South Africa (Zar et al., 2014). The study investigates the role and interaction of risk factors in the environmental, infectious, nutritional, genetic, psychosocial, maternal and immunological spheres, and is one of the first birth cohort studies globally to investigate multiple risk factors for child health. Recruitment from two communities characterized by substantially different psychosocial risk factors will enable a comparison of the impact of such factors on child health and development.

The methods of the larger DCHS study have been described elsewhere (Zar et al., 2014). This paper describes the design and methods of the psychosocial component of the study specifically, and provides baseline psychosocial findings from pregnant women and fathers enrolled in the cohort to date. Given the prevalence and potential impact of parental psychosocial risk factors in these communities and in other LMIC populations, this paper provides data which will potentially be useful in understanding the effects of a range of risk factors on infant health and developmental outcomes both locally and worldwide.

2. Methods

2.1. Design

The birth cohort design recruits pregnant women attending one of two primary health care clinics in the Drakenstein sub-district of the Cape Winelands, Western Cape, South Africa – Mbekweni (serving a black African population) and TC Newman (serving a mixed race population). Consenting mothers are enrolled during pregnancy, and mother–infant dyads are followed longitudinally until children reach 5 years of age. Mothers are asked to request that the father of the index pregnancy attend a single antenatal study visit. Follow-up visits for mother–infant dyads take place at the two primary health care clinics and at Paarl Hospital.

2.2. Setting

The Drakenstein sub-district is a peri-urban area which includes the town of Paarl, and is located 60 km outside of Cape Town, South Africa. This is a stable, accessible, low socioeconomic community that is characterized by a high prevalence of a range of risk factors such as alcohol abuse (Watt et al., 2014), tobacco smoke exposure, HIV, single-parent households and poverty; as well as a high burden of childhood diseases, including infant pneumonia. In this respect, it is representative of many peri-urban regions in South Africa, as well as in other LMICs. The majority of the population accesses health care in the public sector.

2.3. Participants

Pregnant women are eligible to participate if they are 18 years or older, are accessing one of the two primary health care clinics for antenatal care, state no intention to move out of the district within the following year, and sign written informed consent (the study was approved by the Faculty of Health Sciences, Human Research Ethics Committee, University of Cape Town (401/2009), by Stellenbosch University (N12/02/0002), and by the Western Cape Provincial Health Research committee (2011RP45)). Participants are enrolled between 20 and 28 weeks' gestation, upon presenting for an initial antenatal care visit. In addition, consenting fathers of the index pregnancy are enrolled in the study and attend a single antenatal study visit.

2.4. Sample size

The projected sample size for the cohort is 1000 mother–infant dyads. This sample size is designed to provide at least 550 pneumonia episodes, as a primary aim of the DCHS is to investigate risk factors for childhood pneumonia. This sample will provide adequate statistical power to detect relative associations of at least 1.5-fold for prevalent risk factors in the cohort, taking into account an estimated cumulative attrition of 20% over 5 years (including losses due to child mortality). Given potential loss to follow-up between enrolment and birth, approximately 1150 women will be enrolled in the cohort in order to achieve this target sample. At the time of writing, enrolment is ongoing.

3. Measures

After providing consent, participants are asked to complete a battery of self-report and clinician-administered measures at a number of antenatal and postnatal study visits (see Fig. 1), as described below. These measures aim to assess maternal psychosocial risk factors over time, paternal psychosocial risk factors antenatally, and infant development. This combination of methods

Confidential: For Review Only

Measure	Risk factor assessed by measure	Time point of assessment – study visit[1]			
		Enrolment	Antenatal	Birth	Postnatal
Maternal psychosocial measures					
Planning of the Birth/Partner Support	Pregnancy intention and male partner support		X		
SRQ-20	Psychological distress		X		X
Beck Depression Inventory (BDI-II)	Primary tool used to assess depression		X		
Edinburgh Postnatal Depression Rating Scale (EPDS)	Recent depressive symptoms during the ante- and postnatal period		X		X
Alcohol, Smoking and Substance Involvement Screening Test (ASSIST)	Substance use and substance-related risk		X		X
Fagerström Test of Nicotine Dependence	Nicotine dependence	X			X
Urine cotinine	Objective measure of tobacco smoke exposure		X	X	X
Rapid urine dipstick testing	Objective measure of recent illicit substance use		X		
Childhood Trauma Questionnaire – Short Form (CTQ-SF)	Childhood abuse (physical, emotional, and sexual) and childhood neglect (physical and emotional)		X		
Intimate Partner Violence (IPV) Questionnaire	Lifetime and recent (past-year) exposure to emotional, physical and sexual intimate partner violence		X		X
Life Events Questionnaire	Exposure to stressful/negative life events during the past 12 months		X		X
Peritraumatic Distress Inventory	Level of distress experienced during and immediately after a traumatic event		X		
Modified Posttraumatic Stress Disorder Symptom Scale (MPSS)	Rapid screening for PTSD		X		
Paternal psychosocial measures (a subset of the measures detailed above)					
Planning of the Birth/Partner Support (adapted for the paternal context); BDI-II; EPDS (adapted for paternal use); SRQ-20; ASSIST; Fagerström; CTQ-SF; Life Events Questionnaire; Peritraumatic Distress Inventory; MPSS	Birth planning and partner support; depression and psychological distress; substance use and nicotine dependence; childhood trauma; life events; and PTSD		X		
Infant psychosocial measures					
Western Cape Developmental Screening Tool	Infant neurodevelopment	6 week visit; 9 month visit; 18 month visit			
Bayley Scales of Infant and Toddler Development (Bayley-III)	Infant development and developmental delay in a subset of infants	6 month visit; 18 month visit; 24 month visit			

[1] Enrolment, 20-28 weeks' gestation; Antenatal, 28-32 weeks' gestation; Postnatal, various postnatal time points

Fig. 1. Table of antenatal and perinatal psychosocial measures.

will allow the exploration of a range of risk factors and their underlying mechanisms, and will enable a comparison of their impact on child health and developmental outcomes. As such, the DCHS is one of the first birth cohort studies globally to investigate multiple risk factors for child neurodevelopment. Although the majority of measures were originally designed to be self-administered, questionnaires are administered by trained fieldworkers in the participants' preferred language of English, Afrikaans, or isiXhosa, as literacy levels are low in this context. Translation of measures from English to Afrikaans and isiXhosa was done using a standard forward and back-translation process (Smit et al., 2006).

3.1. *Sociodemographic characteristics*

Following an initial enrolment assessment, sociodemographic characteristics are assessed longitudinally. The interviewer-administered sociodemographic questionnaire designed for the purposes of this study was adapted from items used in the South African Stress and Health Study (SASH) (Myer et al., 2008), and includes an assessment of employment status, highest level of education completed, and participants' population group. Race classification was historically required under the apartheid system in South Africa. While we do not intend to reify these categories, information on population group is collected in this study, as persistent health disparities exist across groups in South Africa, and disparities in child health and development related to ethnicity and SES are well-documented worldwide (Cheng and Goodman, 2015).

3.2. *Planning of pregnancy and partner support*

The Planning of the Birth/Partner Support Questionnaire was developed for the purposes of this. The questionnaire assesses pregnancy intention and support received from a male partner. Partner support and ability to rely on a male partner for help are assessed on a frequency scale ranging from 1 ("not at all") to 5 ("extremely"), with higher scores indicating greater support and partner reliability.

3.3. *Psychological distress, depression severity, and psychiatric disorders*

The SRQ-20 (Beusenberg and Orley, 1994) is a WHO-endorsed measure of psychological distress. This tool has been widely used internationally and in South African settings, and has shown good reliability and face validity (Harpham et al., 2003; Rumble et al., 1996). The SRQ-20 consists of 20 items which assess non-psychotic symptoms, including symptoms of depressive and anxiety disorders. Each item is scored according to whether the participant responds in the affirmative (scored as 1) or negative (scored as 0) to the presence of a symptom. Individual items are summed to generate a total score. A cut-off score of $\geq$8 can be used to dichotomize participants into "high risk" versus "low risk", as has been widely used elsewhere (Harpham et al., 2003; Ventevogel et al., 2007).

The Beck Depression Inventory (BDI-II) is a widely-used and reliable measure of depressive symptoms (Beck et al., 1961, 1988, 1996). The BDI-II has shown good validity and internal consistency when used in both psychiatric and non-psychiatric populations (Beck et al., 1988, 1996) and has been used in numerous studies conducted in South Africa (Kagee et al., 2014; Kagee and Martin, 2010; Nel and Kagee, 2013). The BDI-II comprises 21 items, each of which assesses the severity of a symptom of major depression. Each item is assessed on a severity scale ranging from 0 (absence of symptoms) to 3 (severe, often with functional impairment). A total score is then obtained by summing individual item responses, with a higher score indicative of more severe depressive symptoms. A cut-off score of $\geq$20 has been used to dichotomize participants into "probable moderate/severe clinical cases" versus "probable sub-threshold participants" (Lasa et al., 2000).

Confidential: For Review Only

The Edinburgh Postnatal Depression Rating Scale (EPDS) (Cox et al., 1987) is a 10-item self-report measure of recent depressive symptoms. The tool was originally developed for use in postnatal women, and assesses the presence of mood changes characteristic of postnatal depression, under the assumption that normal symptoms experienced during the perinatal period (for example, changes in sleep and appetite) could be misattributed to a depressive disorder on many standard screening tools for depression (Cox et al., 1987). This tool has been validated for use in pregnancy (Murray and Cox, 1990), and is used alongside the BDI-II to measure depression in this study. Each item is scored on a frequency scale ranging from 0 to 3. A total score is then obtained by summing individual item responses, with a higher score indicative of more severe depressive symptoms. A cut-off score of ≥13 has been used to indicate probable depression, as described in the original development of the scale (Cox et al., 1987) and in South Africa (Hartley et al., 2011).

The Mini International Neuropsychiatric Interview (MINI) is an abridged version of the Structured Clinical Interview for DSM-IV (Lecrubier et al., 1997; Sheehan et al., 1997, 1998). This clinician-administered interview provides diagnoses of a range of common mental disorders, and shows good psychometric properties (Lecrubier et al., 1997; Sheehan et al., 1997, 1998). The MINI is administered to a sample of participants, partly in order to obtain data on the clinical validity of measures such as the SRQ-20 and BDI-II in our sample, and partly to study women with PTSD in more detail. As nested sub-studies, these protocols and analyses will be reported elsewhere.

3.4. Substance use

The Alcohol, Smoking and Substance Involvement Screening Test (ASSIST) (WHO ASSIST Working Group, 2002) is a tool that was developed by the WHO to detect and manage substance use among people attending primary health care services. It has shown good reliability and validity in international, multi-site studies (Humeniuk et al., 2008; WHO ASSIST Working Group, 2002). The tool assesses substance use and substance-related risk across 10 categories (tobacco, alcohol, cannabis, cocaine, amphetamine-type stimulants, inhalants, sedatives/sleeping pills, hallucinogens, opioids, and other substances), as well as enquiring about a history of intravenous drug use. Total scores are obtained for each substance by summing individual item responses, with a higher score indicative of greater risk for substance-related health problems. Scores of 0–10 for alcohol and 0–3 for illicit drugs have been used to indicate that a participant is at low risk for substance-related health problems from their current pattern of use; scores of 11–26 for alcohol and 4–26 for illicit drugs to indicate moderate risk; and scores >26 to indicate that a participant is at high risk of experiencing severe problems as a result of their current pattern of use and is likely to be dependent (World Health Organization, 2010).

The Fagerström Test for Nicotine Dependence is a 6-item tool that has been widely-used and provides a rapid assessment of nicotine dependence (Fagerström et al., 1990; Heatherton et al., 1991). It is used in this study to supplement the findings of the tobacco category of the ASSIST. Individual items are scored based on the original development of the scale (Heatherton et al., 1991) and are summed to generate a total score, with a higher score indicative of greater nicotine dependence. Participants are then categorized according to this level of dependence, with scores of 1–2 indicating low dependence; scores of 3–4 indicating low-moderate dependence; scores of 5–7 indicating moderate dependence; and scores of 8 or above indicating high dependence.

Objective measures of substance use are also included in the DCHS. Maternal urine cotinine is measured antenatally and at the time of birth; and for infants at birth, 6–10 weeks, annually, and at the time of pneumonia episodes, as an objective measure of tobacco smoke exposure. In addition, maternal urine samples are tested antenatally via rapid urine dipstick testing for recent use of common illicit substances, including methamphetamines, cocaine, cannabis, methaqualone (locally referred to as mandrax), opiates, and MDMA.

3.5. Exposure to trauma and posttraumatic stress disorder

The Childhood Trauma Questionnaire–Short Form (CTQ-SF) (Bernstein et al., 1994) is a 28-item inventory assessing childhood abuse (physical, emotional, and sexual) and childhood neglect (physical and emotional) occurring up until the age of 12 years. Each item is scored on a frequency scale from 1 ("never true") to 5 ("very often true"), such that each subscale (domain of abuse or neglect) is scored on a spectrum from 5 (no history of abuse or neglect) to 25 (very extreme history of abuse or neglect). Thus, higher scores in each domain signify a greater severity of abuse or neglect. Participants can also be categorized according to the cut-off scores for each domain, as defined in the CTQ manual (Bernstein and Fink, 1998). Respondents may then be dichotomized into those with a history of childhood trauma (those scoring within the "low to moderate", "moderate to severe", or "severe to extreme" category) versus those without (those scoring within the "none or minimal" category). Three items are also included as a Minimization/Denial scale to detect potential under-reporting of abuse by participants. These items are dichotomized (with a response of "never" scored 0, and all other responses scored 1) and added, with a sum total ≥ 1 indicating possible under-reporting (Bernstein and Fink, 1998; Villano et al., 2004).

The Intimate Partner Violence (IPV) Questionnaire used in this study was adapted from the WHO multi-country study (Jewkes, 2002) and the Women's Health Study in Zimbabwe (Shamu et al., 2011) in order to assess lifetime and recent (past-year) exposure to emotional, physical and sexual IPV. Each category of violence is assessed across multiple items measuring the frequency of a number of specified violent acts. Each item is scored using a frequency scale from 1 ("never") to 4 ("many times"). Scoring guidelines were devised for the purposes of this study, and were based on prior work in South Africa (Dunkle et al., 2004). Participants are categorized as having experienced no IPV if all responses are "never"; an isolated incident of IPV if one response is "once"; a low frequency of violence if the response is "once" to more than one item; a mid frequency if they respond "a few times" to at least one item, but do not respond "many times" to any item; and a high frequency if there are any responses of "many times". In addition, those who respond that the violence has occurred during the past 12 months are categorized as having experienced recent (past-year) violence.

The World Mental Health Life Events Questionnaire is a 17-item tool which assesses exposure to stressful/negative life events during the past 12 months. The questionnaire used in this study is based on the items used in the SASH study in South Africa (Myer et al., 2008). A total score is obtained by summing the total number of life events that participants report experiencing during the past 12 months, with higher scores indicating greater exposure to stressful life events.

The Peritraumatic Distress Inventory is a 13-item tool which assesses the level of distress experienced during and immediately after a traumatic event (Brunet et al., 2001). Each item assesses the extent to which a participant experienced a symptom of distress related to the event. Each item is scored using a frequency scale from 0 ("not at all") to 4 ("extremely"). A total score is obtained by calculating the mean response across all 13 items, with higher scores indicating greater distress related to the traumatic event.

The Modified Posttraumatic Stress Disorder Symptom Scale (MPSS) (Foa et al., 1993) is a 17-item interview that mirrors the

Confidential: For Review Only

DSM-IV criteria for posttraumatic stress disorder (PTSD). The tool is used as a rapid screening for PTSD in this study, and was selected for use due to its reasonably good diagnostic validity for PTSD. The MPSS includes items assessing three symptom clusters for PTSD, namely re-experiencing, avoidance/emotional numbing, and increased arousal. Each item is scored on a frequency scale ranging from 0 (absence of symptom) to 3 (symptom occurs five or more times per week/very much/almost always). A final item was added to assess the duration of symptoms, with response options of <1 month; 1–3 months; 3 months–1 year; and >1 year. A proxy variable for PTSD diagnosis can be generated when re-experiencing symptoms total ≥ 1, avoidance/emotional numbing symptoms ≥ 3, increased arousal symptoms ≥ 2, and symptom duration is of at least 1 month.

The Clinician Administered PTSD Scale (CAPS) is an interviewer-administered diagnostic instrument which measures PTSD (Blake et al., 1990, 1995; Weathers et al., 2001). The CAPS has been shown to have excellent psychometric properties (Blake et al., 1990, 1995; Weathers et al., 2001), and is administered to those who score above threshold for PTSD on the MINI in this study, in order to assess PTSD symptom severity.

3.6. Paternal psychosocial measures

A subset of the measures described above is additionally completed by fathers enrolled in the study at one antenatal visit. Specifically, fathers complete the measures related to birth planning and partner support; psychological distress and depression; childhood trauma; life events; self-reported substance use and nicotine dependence; and PTSD, using the MPSS and Peritraumatic Distress Inventory.

3.7. Infant development

The Western Cape Developmental Screening Questionnaire is administered to caregivers of the infants at 6 weeks, 9 months and 18 months of age. The screening tools were developed as part of a Western Cape Government Department of Health provincial primary health care screening program that aims to identify children with moderate and severe neurodevelopmental disabilities for referral to health services for further formal evaluation and management. Each age-specific tool consists of yes/no questions pertaining to the acquisition of developmental milestones in the domains of hearing, vision, gross motor, fine motor, language and communication and psychosocial development. The tools were designed to be administered to children's caregivers by primary health care workers in a clinical setting during routine immunization visits. Prior to the screening program implementation during 2001, the tools were piloted in provincial primary health care settings, including the Drakenstein sub-district. Domains within the 9-month and 18-month screening tools have been validated. In the present study, infants who are identified by the screening tools as being at-risk for neurodevelopmental problems are referred for further assessment via the accepted child health service pathways for the sub-district.

The Bayley Scales of Infant and Toddler Development (Bayley-III) is a comprehensive assessment tool for measuring infant development and assessing developmental delay in children aged 0–2 years (Bayley, 2006). This tool is a gold-standard measure of infant development globally, and has been widely used in LMIC settings, including South Africa (Ballot et al., 2012). In the DCHS, the Bayley-III is administered by a trained professional on a subset of infants at ages 6 months and 2 years in order to assess childhood development over time. The tool consists of five scales of infant development, namely cognitive, language, motor, socio-emotional, and adaptive behavior, and assesses development using direct observation of the infant as well as caregiver report. Each scale is scored according to the Bayley-III manual (Bayley, 2006) using specialized software (Bayley-III Scoring Assistant Update Version 2.0.2 with Bayley-III PDA conduit). Scaled scores for each subscale are calculated using age-specific reference norms, and a cut-off score of 1 or more standard deviations below the mean in any of the subscales has been used to identify infants with poor developmental outcomes.

Nested studies of mother–infant interactions and of infant brain imaging are being conducted. However, as these only include a subsample of participants, they are not detailed or reported here.

4. Results

Enrolment into the DCHS cohort began in March 2012, with approximately 950 women enrolled to date. As fewer than 40% of participants report that they are currently in a stable relationship, the number of fathers enrolled in the study is far lower (less than 10% of the total number of mothers enrolled). Complete baseline psychosocial data is available and presented below for 634 mothers, and 75 fathers.

4.1. Sociodemographic characteristics

Baseline maternal sociodemographic characteristics are presented in Table 1. The median age of mothers at enrolment was 25.6 years (inter-quartile range: 21.8–30.6). Education and employment levels are low at both clinics, with less than half of participants having completed secondary education, and only a quarter reporting that they are currently employed. A minority of participants (39%) reported that they are currently married or cohabiting.

4.2. Pregnancy planning and partner support

Self-report information regarding pregnancy planning and support received from a male partner is presented in Table 1. The majority of participants (64%) had previously been pregnant, and a high proportion (65%) reported that the current pregnancy was unplanned. Most participants reported high levels of male partner support and reliance on their partners for help.

4.3. Psychological distress and depression

Reported rates of psychological distress and depression are high in this population (Table 2; Fig. 2a). Many participants at both clinics ($n = 74$, 23% and $n = 72$, 23%) scored above threshold scores for psychological distress. Overall, 24% of women scored above threshold scores for depression on the BDI-II, with the prevalence of depression significantly higher at Mbekweni (28%) than at TC Newman (20%).

4.4. Exposure to traumatic stressors and PTSD

High levels of exposure to traumatic stressors were reported (Table 2), with many mothers having experienced neglect and abuse during childhood. In addition, reported levels of lifetime and recent IPV are high in this population, particularly among women from TC Newman (Table 2 and Fig. 2b). Almost half of the mothers from this clinic (46%) reported experiencing lifetime emotional IPV, with 38% and 14% reportedly experiencing lifetime physical and sexual IPV respectively. More than half (52%) of the women who reported experiencing a recent negative life event fit the MPSS-criteria for suspected PTSD related to this event.

Confidential: For Review Only

Table 1
Maternal baseline sociodemographic characteristics, pregnancy planning and partner support.

Variable	Mbekweni – n (%)	TC Newman – n (%)	Total – n (%)	P-value[a]
Number of mothers	317 (50)	317 (50)	634 (100)	
Median age at enrolment (IQR)	26.7 (22.0–31.7)	24.7 (21.4–29.0)	25.6 (21.8–30.6)	<0.001
Race				
Black/African	317 (100)	7 (2)	324 (51)	
White	0 (0)	3 (1)	3 (0.5)	
Mixed race	0 (0)	305 (96)	305 (48)	
Other	0 (0)	2 (0.6)	2 (0.3)	
Married/cohabiting	113 (36)	133 (42)	246 (39)	0.103
Educational attainment				
Primary	34 (11)	22 (7)	56 (9)	0.111
Some secondary	168 (53)	158 (50)	326 (51)	
Completed secondary	97 (31)	122 (38)	219 (35)	
Any tertiary	18 (6)	15 (5)	33 (5)	
Currently employed	61 (19)	97 (31)	158 (25)	0.001
Primigravida	103 (32)	126 (40)	229 (36)	0.057
Unplanned pregnancy	219 (69)	196 (62)	415 (65)	0.055
Partner support				
Not at all/slightly supportive	31 (10)	30 (9)	61 (10)	<0.001
Moderately supportive	52 (16)	13 (4)	65 (10)	
Extremely supportive	234 (74)	274 (86)	508 (80)	
Reliance on partner for help				
Not at all/not very often	39 (12)	31 (10)	70 (11)	<0.001
Some of the time	102 (32)	17 (5)	119 (19)	
Most of the time/all of the time	176 (56)	269 (85)	445 (70)	

[a] Variables compared across site using χ^2 tests for categorical variables and Wilcoxon rank sum tests (Mann–Whitney tests) for continuous variables.

4.5. Substance use

Self-reported lifetime use of tobacco (37%), alcohol (41%), and cannabis (10%) was high among mothers, particularly those at TC Newman (Fig. 2c). Lifetime use of other substances was negligible (lower than 4% of participants) in this sample. Among those who reported lifetime substance use, many scored above the threshold for current moderate-high substance-related problems (Table 3). Substance-related problems (moderate and high) over the past 3 months were most frequently reported for tobacco, with 189 mothers (30%) reporting at-risk tobacco use, followed by alcohol use ($n = 68$, 11%) and cannabis use ($n = 14$, 2%). The Fagerström test indicated moderate levels of nicotine dependence, with 35 mothers (6%) reporting moderate-high dependence. Objective measures of tobacco smoke exposure among those for whom these measures were available ($n = 580$) indicated a high prevalence of current exposure to tobacco smoke ($n = 266$, 46%) and current active smoking ($n = 172$, 30%). Objectives measures of illicit substance use confirmed a low prevalence of recent substance use. However, many participants who screened positive for recent substance use showed evidence of recent use of multiple substances.

4.6. Paternal measures

Paternal measures are presented in Table 4. Given the low number of fathers, however, data are not presented separately for each clinic. Among fathers, there is a moderate prevalence of depression (15% according to the BDI-II), and a high lifetime prevalence of substance use, with 65% reporting tobacco use; 65% reporting alcohol use; and 17% reporting cannabis use. Substance-related risk (moderate and high) is particularly prevalent, with 44 fathers (59%) reporting at-risk tobacco use, followed by alcohol use ($n = 37$, 50%) and cannabis use ($n = 9$, 12%). The Fagerström test indicates fairly high levels of nicotine dependence, with 19 fathers (25%) reporting moderate-high dependence. High levels of childhood neglect and abuse were also reported, particularly in the domain of physical neglect (reported by 41% of fathers).

Table 2
Maternal psychological distress and depression; exposure to trauma and PTSD.

Variable	Mbekweni – n (%)	TC Newman – n (%)	Total – n (%)	P-value[a]
Psychological distress and depression				
SRQ-20 – median score (IQR)	4 (1–7)	4 (2–7)	4 (2–7)	0.042
Beck Depression Inventory – median score (IQR)	12 (6–21)	10 (5–18)	11 (5–19)	0.018
Edinburgh Postnatal Depression Rating Scale – median score (IQR)	11 (8–13)	8 (4–13)	10 (6–13)	<0.001
Exposure to trauma and PTSD				
Childhood trauma				
Emotional neglect – above threshold	82 (26)	123 (39)	205 (32)	<0.001
Physical neglect – above threshold	111 (35)	112 (35)	223 (35)	0.934
Emotional abuse – above threshold	71 (22)	130 (41)	201 (32)	<0.001
Physical abuse – above threshold	82 (26)	57 (18)	139 (22)	0.016
Sexual abuse – above threshold	56 (18)	57 (18)	113 (18)	0.917
Lifetime intimate partner violence (IPV)				
Emotional IPV – above threshold	83 (26)	145 (46)	228 (36)	<0.001
Physical IPV – above threshold	87 (27)	121 (38)	208 (33)	0.004
Sexual IPV – above threshold	24 (8)	45 (14)	69 (11)	0.007
Life events – median (IQR)	1 (0–3)	2 (1–4)	2 (0–3)	<0.001
Peritraumatic Distress Inventory – median (IQR)	1.2 (0.5–1.8)	1.2 (0.5–1.8)	1.2 (0.5–1.8)	0.668

[a] Variables compared across site using χ^2 tests for categorical variables and Wilcoxon rank sum tests (Mann–Whitney tests) for continuous variables.

Confidential: For Review Only

Table 3
Maternal substance use.

Variable	Mbekweni – n (%)	TC Newman – n (%)	Total – n (%)
Tobacco use: substance-related risk			
Moderate risk	13 (4)	140 (44)	153 (24)
High risk	3 (1)	33 (10)	36 (6)
Tobacco dependence (self-reported)			
Low dependence	3 (1)	60 (19)	63 (10)
Low-moderate dependence	6 (2)	45 (14)	51 (8)
Moderate dependence	2 (0.6)	31 (10)	33 (5)
High dependence	0 (0)	2 (0.6)	2 (0.3)
Alcohol use: Substance-related risk			
Moderate risk	9 (3)	40 (13)	49 (8)
High risk	14 (4)	5 (2)	19 (3)
Cannabis use: Substance-related risk			
Moderate risk	1 (0.3)	9 (3)	10 (2)
High risk	0 (0)	4 (1)	4 (0.6)
Tobacco smoke exposure (objective; $n = 580$)			
Non-smoker	106 (36)	36 (13)	142 (24)
Passive smoker/exposed	157 (53)	109 (38)	266 (46)
Active smoker	31 (11)	141 (49)	172 (30)
Illicit substance use (objective; $n = 569$)			
Methamphetamines	0 (0)	14 (5)	14 (2)
Cocaine	0 (0)	0 (0)	0 (0)
Cannabis	1 (0.3)	20 (7)	21 (4)
Methaquolone (mandrax)	0 (0)	6 (2)	6 (1)
Opiates	1 (0.3)	1 (0.4)	2 (0.4)
MDMA	0 (0)	3 (1)	3 (0.5)

5. Discussion

Several aspects of the methods outlined here deserve further consideration, and several findings from these baseline data warrant further discussion.

Table 4
Paternal psychosocial risk factors.

Variable	n (%)
Number of fathers	75
Beck Depression Inventory – median score (IQR)	5 (1–15)
Beck Depression Inventory – above threshold ≥20	11 (15)
Edinburg Postnatal Depression Rating Scale – median score (IQR)	7 (4–10)
Edinburg Postnatal Depression Rating Scale – above threshold ≥13	12 (16)
SRQ-20 – median score (IQR)	2 (0–4)
SRQ-20 – above threshold ≥8	9 (12)
Tobacco use	
Self-reported lifetime use	49 (65)
Substance-related risk	
Moderate risk	29 (39)
High risk	15 (20)
Tobacco dependence (self-reported)	
Low dependence	13 (17)
Low-moderate dependence	13 (17)
Moderate dependence	16 (21)
High dependence	3 (4)
Alcohol use	
Self-reported lifetime use	49 (65)
Substance-related risk	
Moderate risk	11 (15)
High risk	26 (35)
Cannabis use	
Self-reported lifetime use	13 (17)
Substance-related risk	
Moderate risk	7 (9)
High risk	2 (3)
Childhood trauma	
Emotional neglect – above threshold	20 (27)
Physical neglect – above threshold	31 (41)
Emotional abuse – above threshold	19 (25)
Physical abuse – above threshold	21 (28)
Sexual abuse – above threshold	13 (17)
Life events – median (IQR)	2 (0–4)
Suspected PTSD	14 (19)
Peritraumatic Distress Inventory – median (IQR)	0.9 (0.2–1.5)

First, these data reinforce the point that in many parts of the globe, pregnant mothers and their infants face considerable health challenges. This cohort has low levels of educational attainment and employment, and unplanned pregnancy is common. There is a high prevalence of antenatal psychological distress and depression, consistent with previous work in the Western Cape (Dewing et al., 2013; Hartley et al., 2011; Roos et al., 2013; Tomlinson et al., 2014). Substance use, particularly tobacco and alcohol use, is common, again consistent with prior local work (Dewing et al., 2013; Petersen Williams et al., 2014; Vythilingum et al., 2012). In addition, there is a high reported prevalence of IPV within this cohort, as has been previously found during pregnancy in South Africa (Groves et al., 2014).

Second, there is considerable variance within the sample. The legacy of apartheid has affected different communities disparately, and it is notable that the two clinics studied here exhibit differing baseline characteristics. For example, participants at Mbekweni are shown to have higher levels of depression; with those at TC Newman reporting a higher prevalence of substance use and IPV. The higher levels of both substance use and reported IPV observed in TC Newman participants is consistent with previous literature which demonstrates a clear link between substance use and both perpetration of and victimization from IPV (World Health Organization, 2012). Furthermore, despite the adverse circumstances that many pregnant women must endure, there is some evidence of social capital in this cohort (e.g. many consider their partners a support), and a considerable number do not show evidence of psychological distress or depression. While clinical and public mental health interventions should be made available to those in need, it is also important to emphasize the resilience of the communities and their members.

Third, within a complex multidisciplinary study such as this, there are inevitably many claims on participants' time. Thus, only a limited number of psychosocial measures can be administered, and a balance needs to be struck between efficiently assessing a limited range of key issues (e.g. psychological distress, substance use, IPV), and addressing a broader range of possibly relevant factors. One strategy employed is the use of nested sub-studies, which allow exploration of particular questions in greater depth. A particular advantage of our site in Cape Town, for example, is that such substudies can include advanced methods such as infant brain imaging.

Confidential: For Review Only

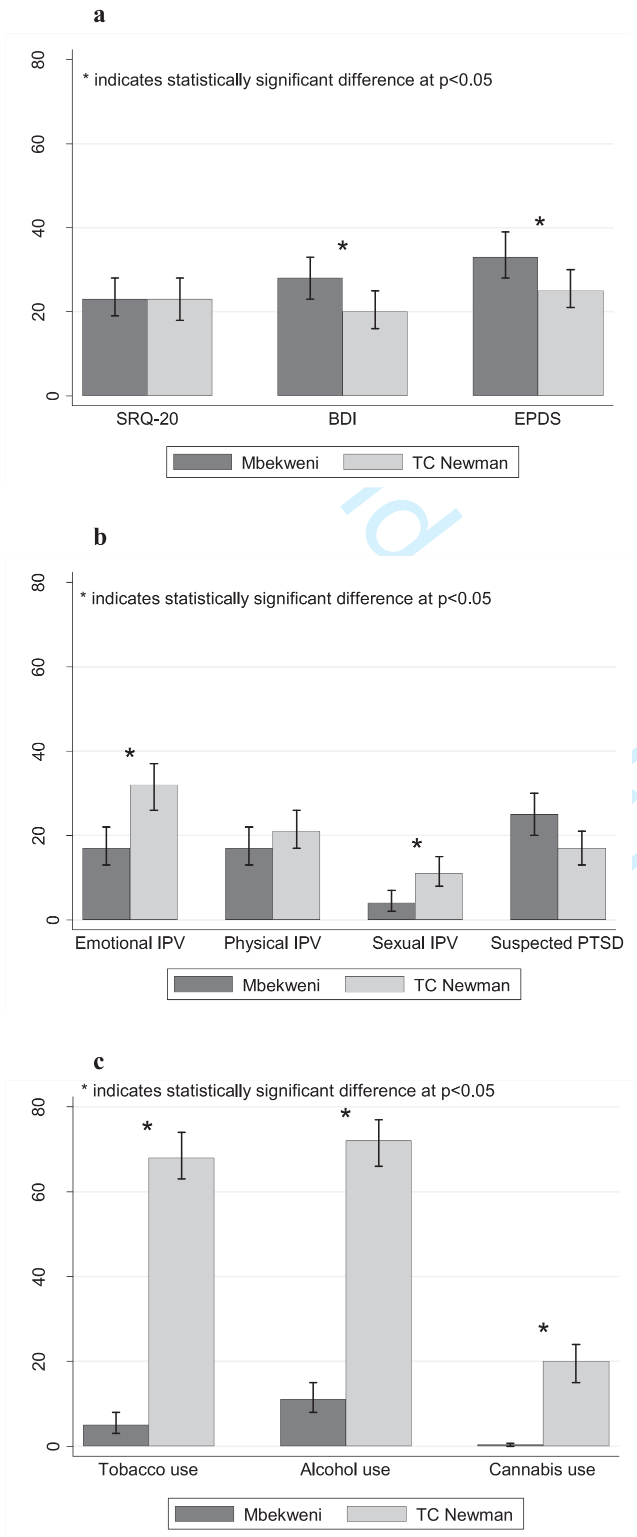

Fig. 2. (a) Prevalence of maternal psychological distress and depression across recruitment site. (b) Prevalence of recent intimate partner violence (IPV) and suspected PTSD across recruitment site. (c) Prevalence of self-reported lifetime substance use across recruitment site.

While our focus here has been on presenting the baseline findings on a small number of core measures, we hope to present sub-study results in the future.

Fourth, it is worth mentioning the range of methodological issues that arise when assessing participants from diverse cultural and language groups. For example, translation of questionnaires needs to be done with great care in order to ensure semantic equivalence (Smit et al., 2006). Furthermore, given the low levels of literacy among our respondents, fieldworkers provide help in administering questionnaires that are ordinarily self-administered. Important limitations may of course ensue, for example respondents may be reluctant to disclose personal or embarrassing information. However, in our setting the benefits of interviewer-administration outweigh the potential for bias, as understanding and correct completion of the questionnaires is ensured. In our view, the longitudinal design of this study fosters a close and open relationship between participants and members of the research team.

Finally, it is worth considering some of the ethical aspects surrounding research such as this. Given the high levels of psychopathology and of exposure to traumatic stressors in this cohort, there was a need to develop standardized clinical protocols for referral of at-risk participants. Fortunately, a basic public health system is in place in the Drakenstein sub-district, and is able to provide care for women with severe psychological distress or depression. However, there remains an insufficiency of public health resources to assist all women in the community. It is our hope that by enrolling in the study, women are guaranteed a minimal level of care; and that the data emerging from this study will be useful in understanding health outcomes in this and other LMIC populations, thus contributing to the advancement of relevant interventions.

In summary, the DCHS cohort is representative of many populations of pregnant mothers and their infants – both locally and worldwide – who face considerable health challenges. While much has been discovered about the effects of early adversity on subsequent health outcomes, relatively little work has been done in LMICs, where psychobiological and psychosocial risk factor profiles differ from those in high-income countries. Further longitudinal assessment of mothers and children may therefore address a critical gap by clarifying the ways in which risk factors intersect and interact, and delineating underlying mechanisms which impact on child health. Such work may ultimately inform clinical and public health interventions appropriate to the South African and other LMIC contexts.

Acknowledgments

We thank the study staff and the staff at Paarl Hospital, and at Mbekweni and TC Newman clinics for their support of the study. We thank the families and children who participated in this study. Support for this study was provided by the Bill and Melinda Gates Foundation [OPP 1017641] and by the National Institute of Mental Health (NIMH) Brain Disorders in the Developing World: Research Across the Lifespan program (grant number R21 MH098662). DJS, NK, AR and HJZ are supported by the Medical Research Council of South Africa.

References

Anda RF, Felitti VJ, Bremner JD, Walker JD, Whitfield C, Perry BD, et al. The enduring effects of abuse and related adverse experiences in childhood: a convergence of evidence from neurobiology and epidemiology. Eur Arch Psychiatry Clin Neurosci 2006;256:174–86.
Ballot DE, Potterton J, Chirwa T, Hilburn N, Cooper PA. Developmental outcome of very low birth weight infants in a developing country. BMC Pediatrics 2012;12:11.
Ban L, Gibson JE, West J, Tata LJ. Association between perinatal depression in mothers and the risk of childhood infections in offspring: a population-based cohort study. BMC Public Health 2010;10:799.
Bayley N. Bayley Scales of Infant and Toddler Development. 3rd ed. San Antonio, TX: Harcourt Assessment, Inc.; 2006.
Beck AT, Steer RA, Brown GK. Manual for the Beck Depression Inventory-II. San Antonio: Psychological Corporation; 1996.

For peer review only

Beck AT, Steer RA, Garbin MG. Psychometric properties of the Beck Depression Inventory: twenty-five years of evaluation. Clin Psychol Rev 1988;8:77–100.

Beck AT, Ward CH, Mendelson M, Mock J, Erbaugh J. An inventory for measuring depression. Arch Gen Psychiatry 1961;4:53–63.

Beijers R, Jansen J, Riksen-Walraven M, de Weerth C. Maternal prenatal anxiety and stress predict infant illnesses and health complaints. Pediatrics 2010;126(2):e401.

Bernstein D, Fink L. Childhood Trauma Questionnaire: a retrospective self-report. San Antonio: The Psychological Corporation; 1998.

Bernstein DP, Fink L, Handelsman L, Foote J, Lovejoy M, Wenzel K, et al. Initial reliability and validity of a new retrospective measure of child abuse and neglect. Am J Psychiatry 1994;151(8):1132–6.

Beusenberg M, Orley J. A user's guide to the self reporting questionnaire (SRQ). Division of Mental Health. Geneva: World Health Organization; 1994.

Blake DD, Weathers FW, Nagy LM, Kaloupek DG, Klauminzer G, Charney DS, et al. A clinician rating scale for assessing current lifetime PTSD: the CAPS-1. Behav Ther 1990;13:187–8.

Blake DD, Weathers FW, Nagy LM, Kaloupek DG, Gusman FD, Charney DS, et al. The development of a clinician-administered PTSD scale. J Trauma Stress 1995;8:75–90.

Brunet A, Weiss DS, Metzler TJ, Best SR, Neylan TC, Rogers C, et al. The Peritraumatic Distress Inventory: a proposed measure of PTSD criterion A2. Am J Psychiatry 2001;158:1480–5.

Carter AS, Wagmiller RJ, Gray SAO, McCarthy KJ, Horwitz SM, Briggs-Gowan MJ. Prevalence of DSM-IV disorder in a representative, healthy birth cohort at school entry: sociodemographic risks and social adaptation. J Am Acad Child Adolesc Psychiatry 2010;49(7):686–98.

Cheng TL, Goodman E. The Committee on Pediatric Research, Race, ethnicity, and socioeconomic status in research on child health. Pediatrics 2015;135(1):e225–37.

Cox JL, Holden JM, Sagovsky R. Detection of postnatal depression: development of the 10- item Edinburgh Postnatal Depression Scale. Br J Psychotherapy 1987;150:782–6.

Dewing S, Tomlinson M, le Roux IM, Chopra M, Tsai AC. Food insecurity and its association with co-occurring postnatal depression, hazardous drinking, and suicidality among women in peri-urban South Africa. J Affect Disord 2013;150:460–5.

Dunkle KL, Jewkes RK, Brown HC, Gray GE, McIntryre JA, Harlow SD. Gender-based violence, relationship power, and risk of HIV infection in women attending antenatal clinics in South Africa. Lancet 2004;363(9419):1415–21.

Fagerström KO, Heatherton TF, Kozlowski LT. Nicotine addiction and its assessment. Ear Nose Throat J 1990;69:763–8.

Flach C, Leese M, Heron J, Evans J, Feder G, Sharp D, et al. Antenatal domestic violence, maternal mental health and subsequent child behaviour: a cohort study. BJOG 2011;118:1383–91.

Foa EB, Riggs DS, Dancu CV, Rothbaum BO. Reliability and validity of a brief instrument for assessing posttraumatic stress disorder. J Trauma Stress 1993;6(4):459–73.

Gluckman PD, Hanson MA, Cooper C, Thornburg KL. Effect of in utero and early-life conditions on adult health and disease. N Engl J Med 2008;359(1):61–73.

Groves AK, Moodley D, McNaughton-Reyes L, Martin SL, Foshee V, Maman S. Prevalence, rates and correlates of intimate partner violence among South African women during pregnancy and the postpartum period. Matern Child Health J 2014.

Harpham T, Reichenheim M, Oser R, Thomas E, Hamid N, Jaswal S, et al. Measuring mental health in a cost-effective manner. Health Policy Plan 2003;18(3):344–9.

Hartley M, Tomlinson M, Greco E, Comulada WS, Stewart J, le Roux I, et al. Depressed mood in pregnancy: prevalence and correlates in two Cape Town peri-urban settlements. Reprod Health 2011;8:9.

Heatherton TF, Kozlowski LT, Frecker RC, Fagerström KO. The Fagerström Test for Nicotine Dependence: a revision of the Fagerstrom Tolerance Questionnaire. Br J Addict 1991;86:1119–27.

Heim C, Shugart M, Craighead WE, Nemeroff CB. Neurobiological and psychiatric consequences of child abuse and neglect. Dev Psychobiol 2010;52:671–90.

Hollams EM, de Klerk NH, Holt PG, Sly PD. Persistent effects of maternal smoking during pregnancy on lung function and asthma in adolescents. Am J Respir Crit Care Med 2014;189(4):401–7.

Humeniuk RE, Ali RA, Babor TF, Farrell M, Formigoni ML, Jittiwutikarn J, et al. Validation of the Alcohol Smoking and Substance Involvement Screening Test (ASSIST). Addiction 2008;103(6):1039–47.

Jewkes. R Intimate partner violence: causes and prevention. Lancet 2002;359(9315):1423–9.

Kagee A, Martin L. Symptoms of depression and anxiety among a sample of South African patients living with HIV. AIDS Care 2010;22(3):381–8.

Kagee A, Nel A, Saal W. Factor structure of the Beck Depression Inventory-II among South Africans receiving antiretroviral therapy. AIDS Care 2014;26(2):257–62.

Kingston D, Tough S, Whitfield H. Prenatal and postpartum maternal psychological distress and infant development: a systematic review. Child Psychiatry Hum Dev 2012;43:683–714.

Lasa L, Ayuso-Mateos JL, Vázquez-Barquero JL, Díez-Manrique FJ, Dowrick CF. The use of the Beck Depression Inventory to screen for depression in the general population: a preliminary analysis. J Affect Disord 2000;57(1-3):261–5.

Lecrubier Y, Sheehan D, Weiller E, Amorim P, Bonora I, Sheehan K, et al. The MINI International Neuropsychiatric Interview (M.I.N.I.) a short diagnostic structured interview: reliability and validity according to the CIDI. Eur Psychiatry 1997;12:224–31.

Lund C. Poverty and mental health: towards a research agenda for low and middle-income countries, Commentary on Tampubolon and Hanandita. Soc Sci Med 2014;111:134–6.

Maggi S, Irwin LJ, Siddiqi A, Hertzman C. The social determinants of early child development: an overview. J Paediatr Child Health 2010;46:627–35.

Murray D, Cox JL. Screening for depression during pregnancy with the Edinburgh Depression Scale (EPDS). J Reprod Infant Psychol 1990;8:99–107.

Myer L, Stein DJ, Grimsrud A, Seedat S, Williams DR. Social determinants of psychological distress in a nationally-representative sample of South African adults. Soc Sci Med 2008;66(8):1828–40.

Nel A, Kagee A. The relationship between depression, anxiety and medication adherence among patients receiving antiretroviral treatment in South Africa. AIDS Care 2013;25(8):948–55.

Petersen Williams P, Jordaan E, Mathews C, Lombard C, Parry CDH. Alcohol and other drug use during pregnancy among women attending midwife obstetric units in the Cape Metropole, South Africa. Adv Prev Med 2014 (Article ID 871427).

Roos A, Faure S, Lochner C, Vythilingum B, Stein DJ. Predictors of distress and anxiety during pregnancy. Afr J Psychiatry 2013;16:118–22.

Rumble S, Swartz L, Parry C, Zwarenstein M. Prevalence of psychiatric morbidity in the adult population of a rural South African village. Psychol Med 1996;26:997–1007.

Shamu S, Abrahams N, Temmerman M, Musekiwa A, Zarowsky C. A systematic review of African studies on intimate partner violence against pregnant women: prevalence and risk factors. PLoS ONE 2011;6(3):e17591.

Sheehan DV, Lecrubier Y, Harnett-Sheehan K, Amorim P, Janavs J, Weiller E, et al. The Mini-International Neuropsychiatric Interview (M.I.N.I.): the development and validation of a structured diagnostic psychiatric interview for DSMIV and ICD-10. J Clin Psychiatry 1998;59(Suppl. 20):22–33.

Sheehan DV, Lecrubier Y, Harnett-Sheehan K, Janavs J, Weiller E, Bonora LI, et al. Reliability and validity of the MINI International Neuropsychiatric Interview (M.I.N.I.): according to the SCID-P. Eur Psychiatry 1997;12:232–41.

Smit J, van den Berg CE, Bekker L-G, Seedat S, Stein DJ. Translation and cross-cultural adaptation of a mental health battery in an African setting. Afr Health Sci 2006;6(4):215–22.

Stocks J, Hislop A, Sonnappa S. Early lung development: lifelong effect on respiratory health and disease. Lancet Respir Med 2013;1:728–42.

Taylor SE. Mechanisms linking early life stress to adult health outcomes. Proc Natl Acad Sci USA 2010;107(19):8507–12.

Tomlinson M, O'Connor MJ, le Roux IM, Stewart J, Mbewu N, Harwood J, et al. Multiple risk factors during pregnancy in South Africa: the need for a horizontal approach to perinatal care. Prev Sci 2014;15:277–82.

Ventevogel P, De Vries G, Scholte WF, Shinwari NR, Faiz H, Nassery R, et al. Properties of the Hopkins Symptom Checklist-25 (HSCL-25) and the Self-Reporting Questionnaire (SRQ-20) as screening instruments used in primary care in Afghanistan. Soc Psychiatry Psychiatr Epidemiol 2007;42(4):328–35.

Villano CL, Cleland C, Rosenblum A, Fong C, Nuttbrock L, Marthol M, et al. Psychometric utility of the childhood trauma questionnaire with female street-based sex workers. J Trauma Dissociation 2004;5(3):33–41.

Vythilingum B, Roos A, Faure SC, Geerts L, Stein DJ. Risk factors for substance use in pregnant women in South Africa. S Afr Med J 2012;102(11).

Walker SP, Wachs TD, Grantham-McGregor S, Black MM, Nelson CA, Huffman SL, et al. Inequality in early childhood: risk and protective factors for early child development. Lancet 2011;378:1325–38.

Walker SP, Wachs TD, Meeks Gardner J, Lozoff B, Wasserman GA, Pollitt E, et al. Child development: risk factors for adverse outcomes in developing countries. Lancet 2007;369:145–57.

Watt MH, Eaton LA, Choi KW, Velloza J, Kalichman SC, Skinner D, et al. It's better for me to drink, at least the stress is going away: perspectives on alcohol use during pregnancy among South African women attending drinking establishments. Soc Sci Med 2014;116:119–25.

Weathers FW, Keane TM, Davidson JRT. Clinician-administered PTSD scale: a review of the first ten years of research. Depress Anxiety 2001;13:132–56.

WHO ASSIST Working Group. The Alcohol, Smoking and Substance Involvement Screening Test (ASSIST): development, reliability and feasibility. Addiction 2002;97(9):1183–94.

World Health Organization. Intimate Partner Violence. Geneva: World Health Organization; 2012.

World Health Organization. The Alcohol, Smoking and Substance Involvement Screening Test (ASSIST): Manual for Use in Primary Care. Geneva: World Health Organization; 2010.

Zar HJ, Barnett W, Myer L, Stein DJ, Nicol MP. Investigating the early-life determinants of illness in Africa: the Drakenstein Child Health Study. Thorax 2014., http://dx.doi.org/10.1136/thoraxjnl-2014-206242, pii:thoraxjnl-2014-206242.

Confidential: For Review Only

Downloaded from http://thorax.bmj.com/ on March 5, 2018 - Published by group.bmj.com

AUDIT, RESEARCH AND GUIDELINE UPDATE

Investigating the early-life determinants of illness in Africa: the Drakenstein Child Health Study

H J Zar,[1] W Barnett,[1] L Myer,[2] D J Stein,[3] M P Nicol[4]

OPEN ACCESS

[1]Department of Paediatrics and Child Health, Red Cross War Memorial Children's Hospital, University of Cape Town, Cape Town, South Africa
[2]Division of Epidemiology and Biostatistics, School of Public Health & Family Medicine, University of Cape Town, Cape Town, South Africa
[3]Department of Psychiatry and Mental Health and MRC Unit on Anxiety & Stress Disorders, University of Cape Town, Cape Town, South Africa
[4]Department of Clinical Laboratory Sciences, University of Cape Town, Cape Town, South Africa

Correspondence to
Professor Heather J Zar, Department of Paediatrics and Child Health, 5th Floor, ICH Building, Red Cross War Memorial Children's Hospital, Cape Town 7700, South Africa; heather.zar@uct.ac.za

Received 29 August 2014
Accepted 30 August 2014
Published Online First 16 September 2014

To cite: Zar HJ, Barnett W, Myer L, *et al*. *Thorax* 2015;**70**:592–594.

ABSTRACT
Respiratory disease is the predominant cause of illness in children globally. We describe a unique multidisciplinary South African birth cohort, the Drakenstein Child Health Study (DCHS), to investigate the incidence, risk factors, aetiology and long-term impact of early lower respiratory tract infection (LRTI) on child health. Pregnant women from a poor, peri-urban community with high exposure to infectious diseases and environmental risk factors are enrolled with 1000 mother–child pairs followed for at least 5 years. Biomedical, environmental, psychosocial and demographic risk factors are longitudinally measured. Environmental exposures are measured using monitors placed at home visits. Lung function is measured in children at 6 weeks, annually and during LRTI episodes. Microbiological investigations including microbiome and multiplex PCR measures are done longitudinally and at LRTI episodes. The DCHS is a unique African birth cohort study that uses sophisticated measures to comprehensively investigate the early-life determinants of child health in an impoverished area of the world.

BACKGROUND
Globally, childhood respiratory illness is a major cause of morbidity and mortality.[1] Childhood pneumonia or lower respiratory tract infection (LRTI) remains the main cause of mortality, while asthma is the most common non-communicable disease. The burden of childhood respiratory illness is highest in low and middle income countries (LMICs), reflecting the demographic profile, epidemiology of LRTIs, distribution of risk factors and weak health systems in many settings.[2] Several risk factors for LRTI have been identified, but there are limited data on the interaction or cumulative effects of these on incidence and long-term health. Further, there are few data from LMICs on the impact of immunisation with conjugate vaccines against *Streptococcus pneumoniae* and *Haemophilus influenzae* b on the burden and aetiology of LRTI.[3]

Several birth cohort studies in high-income countries have investigated the determinants of chronic respiratory illness, including wheezing illness or asthma.[4] These suggest that LRTI early in life may result in chronic illness. A meta-analysis reported a 5%–14% risk of developing a major respiratory sequelae following childhood LRTI.[5] Malnutrition, poverty or crowded living conditions increase this risk. Other factors such as allergen, biomass or bacterial exposure may also lead to chronic disease dependent on the intensity, duration or timing of exposure and genetic susceptibility. Although the burden of LRTI is especially high in African children, there have been no similar cohort studies conducted in this setting.

The Drakenstein Child Health Study (DCHS) aims to investigate the epidemiology, risk factors, aetiology and long-term impact of early LRTI on child health in an LMIC. This study is unique given its location in a poor African community and the ability to undertake sophisticated measures of disease and of risk factors longitudinally from the antenatal period through early childhood. The focus on LRTI addresses a global priority in child health, for which no similar birth cohort studies have been undertaken.

METHODS
The DCHS is a population-based birth cohort study in the Drakenstein area in Paarl, a peri-urban area, 60 km outside Cape Town, South Africa. Pregnant women are enrolled in their second trimester and followed through childbirth; thereafter mother–child pairs are followed until children are at least 5 years old (figure 1). Maternal, paternal and child health are investigated through longitudinal measurements of risk factors in seven areas (environmental, infectious, nutritional, genetic, psychosocial, maternal and immunological) that may impact on child health. Intensive aetiological and risk factor investigations are done during an episode of childhood LRTI.

Study population
The local community of approximately 200 000 people is of low socio-economic status, live in informal housing or crowded conditions and have high levels of unemployment. Infectious diseases including pneumonia, HIV (antenatal prevalence approximately 30%) and tuberculosis (annual reported incidence 293/100 000) are common. There is a high prevalence of tobacco smoke exposure, alcohol misuse, malnutrition and other poverty-related exposures. Pneumonia is the predominant cause of childhood hospitalisation and death, with the estimated incidence similar to the reported LMIC incidence of 0.22 per child-year in early life.[2] The population is stable, with little immigration or emigration. More than 90% of the population access healthcare in the public sector including antenatal and child health services. The public health system comprises 23 primary health clinics and one hospital, Paarl Hospital, where all births and hospital care occur. The well-established, free primary healthcare system provides childhood

Confidential: For Review Only

Downloaded from http://thorax.bmj.com/ on March 5, 2018 - Published by group.bmj.com

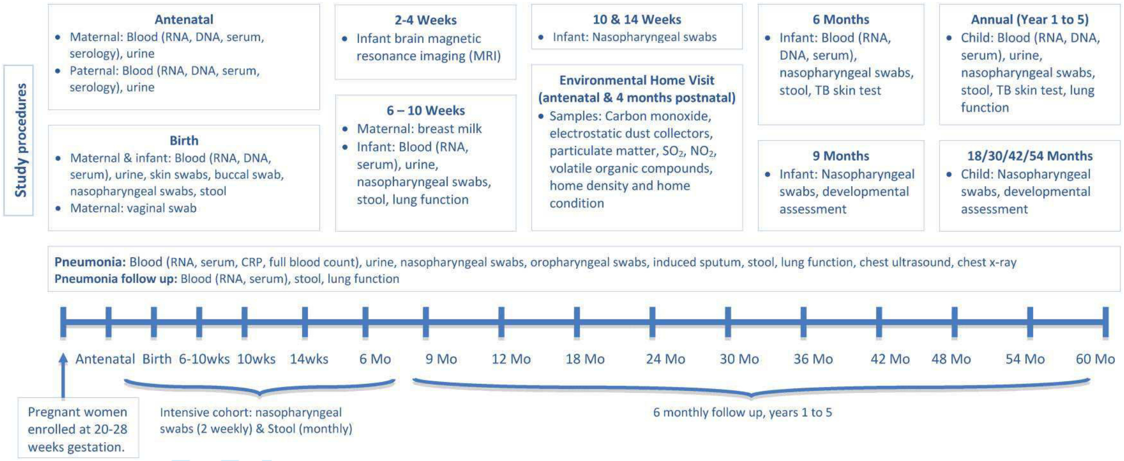

Figure 1 Outline of study visits and samples collected.

immunisations including 13-valent pneumococcal and *H influenzae* b vaccines as part of the national immunisation schedule.

Consenting pregnant women are enrolled from two primary health clinics serving different populations—TC Newman (serving a mixed race population) and Mbekweni (serving a black African population). Pregnant women who are not enrolled are included in a control cohort; these mother–infant pairs are followed annually to compare outcomes with the active cohort.

Study procedures and follow-up

Antenatal and postnatal visits are at primary healthcare clinics, while birth, 6-week and annual study visits occur at Paarl hospital (figure 1). Fathers, as identified by mothers, are invited to participate in an antenatal study visit. Infants attend study visits synchronised with the national programme where feasible at 6, 10 and 14 weeks, and 6, 9, 18, 30, 36, 42, 54 and 60 months. Two home visits (antenatally and 4 months postnatally) are done to investigate environmental risk factors.

Measures

Comprehensive data including biomedical, environmental, psychosocial, demographic, physical and mental health of the mother, father and child and intercurrent morbidity are collected. Specimens (blood, urine, stool, respiratory) are longitudinally taken (figure 1). Urine cotinine, to investigate tobacco smoke exposure, is longitudinally measured. Monitors measuring nitrogen dioxide, sulfur dioxide, carbon monoxide, volatile organic compounds and particulate matter (PM10) exposure over 24 h to 2 weeks are placed in homes; electrostatic dust collectors collect household dust over 2 weeks.

Infant lung function, undertaken for the first time in an African setting, is measured at 6 weeks and annually at Paarl hospital. State-of-the-art measurements in unsedated children during sleep include tidal breathing, exhaled nitric oxide, forced oscillation technique and sulfur hexafluoride multiple breath washout. Lung function is also measured during a LRTI and 4–6 weeks thereafter. Chronic respiratory disease measurements include symptoms, clinical data, lung function and chest X-ray and ultrasound (during an LRTI).

Child neurodevelopmental outcomes are assessed longitudinally with a subsample of infants undergoing brain MRI.

All children have six monthly nasopharyngeal swabs (NPs) and stool specimens collected, while a subset intensive cohort have two weekly NPs and monthly stool samples in the first year. These specimens will enable longitudinal delineation of the child's nasopharyngeal and stool microbiome using targeted (bacterial culture, multiplex real-time PCR for viral and bacterial pathogens) and non-targeted approaches (16srRNA gene sequencing). A similar approach is used for detailed investigation of LRTI aetiology on NP and induced sputum specimens. The maternal microbiome (stool, vaginal, skin, breast milk, NPs) is also studied perinatally (figure 1). The predictive value of the child's microbiome for development of LRTI or chronic respiratory illness is a key area of study.

Specimens from mothers, fathers, children and the environment are processed in a central research laboratory and stored at −80°C, creating a large biobank for future studies.

Surveillance, community engagement and cohort retention

Measurement of LRTI includes ambulatory and hospitalised pneumonia cases, severe or very severe pneumonia, as defined by WHO criteria. Strong surveillance systems have been established using healthcare workers, cell phones and active surveillance at health facilities. Trained community field workers promote community engagement and enable home visits even in areas where violent crime is common. Several strategies to promote cohort retention are used including automated study visit reminders, a close working relationship with clinical staff, a cell phone system enabling two-way communication with study participants at all times and regular follow-up synchronised with routine visits.

Ethics

Written informed consent from mothers is renewed annually; informed consent is also obtained from fathers. The study was approved by the Ethics Committee of the Faculty of Health Sciences, University of Cape Town, by Stellenbosch University and the Western Cape Provincial Research committee.

Sample size

The sample size of 1000 mother-infant pairs is designed to provide at least 550 pneumonia episodes for analyses of LRTI incidence and determinants. We estimate cumulative attrition over 5 years of 20% (including losses due to child mortality) and an expected incidence of LRTI similar to that reported in LMIC.[2] This sample will provide adequate statistical power to detect relative associations of at least 1.5-fold for prevalent risk factors.

Confidential: For Review Only

Downloaded from http://thorax.bmj.com/ on March 5, 2018 - Published by group.bmj.com

CONCLUSION

The DCHS is a unique birth cohort that enables repeated assessments over time to investigate the incidence, aetiology, determinants and long-term impact of early childhood LRTI, on child health in a LMIC. Prenatal recruitment allows assessment of exposures during gestation and after birth, use of a population-based sample helps to eliminate selection biases inherent in case-based approaches and the cohort design ensures clear identification of the time-order of associations. Early, repeated measurement of a broad range of risk factors by a multidisciplinary team and of outcomes with the ability to perform sophisticated measures such as infant lung function, neuroimaging and microbiome investigations are further strengths of the study.

A key focus is the ability to investigate in detail episodes of ambulatory and hospitalised LRTI with state-of-the-art aetiological investigations in the context of high coverage with new conjugate vaccines. Further, the ability to longitudinally measure chronic lung disease presents the opportunity to investigate a key association between childhood communicable and non-communicable diseases. This is a critical area given the importance of chronic respiratory illness as a major non-communicable disease. The study also provides a platform for additional and future studies of child health through the creation of a large, comprehensive biobank.

The study is unique in undertaking longitudinal measurements of child health in the context of a poor African community with a high burden of disease. While most LRTI occurs in LMICs, this is one of the first birth cohort studies in such a setting to investigate these crucial issues using objective, state-of-the-art measures to identify new, improved strategies for prevention and treatment of disease.

Acknowledgements We thank the study staff, the clinical and administrative staff of the Western Cape Government Health Department at Paarl Hospital and at the clinics for support of the study. We thank our collaborators and the masters, doctoral and postdoctoral students for work on the study. We acknowledge the advice received from members of the study International Advisory Board.

Contributors HJZ is the principal investigator of the DCHS who conceived and designed the study and obtained core funding, together with LM who leads the epidemiology and data aspects, DJS who leads the psychosocial aspects and MPN who leads the microbiological aspects. HJZ provides overall oversight. WB is the study coordinator responsible for operational aspects. All authors contributed to the final version of the manuscript.

Funding The study is funded by the Bill and Melinda Gates Foundation (OPP 1017641). Environmental aspects are funded by the National Research Foundation (NRF), the Discovery Foundation, the South African Medical Research Council (SA-MRC) and the Clinical Infectious Diseases Research Initiative at the University of Cape Town (UCT). Lung function has been funded through Wellcome Trust (098479/Z/12/Z), a UCT Research award, a Thrasher Research award and a World Wide University network grant. Microbiome studies are supported by the NIH, USA (U01AI110466), and Wellcome Trust (102429/Z/13/Z). Neuroimaging and neurodevelopmental aspects have been funded through the ABMRF/Foundation for Alcohol Research, USA, the SA-MRC and the NRF. Psychosocial aspects have been funded through NIH grant (R21 MH102001). DJS is supported by the SA-MRC.

Competing interests None declared.

Ethics approval Human Research Ethics Committee, Faculty of Health Sciences, University of Cape Town, by Stellenbosch University and the Western Cape Provincial Research Committee.

Provenance and peer review Not commissioned; internally peer reviewed.

Open Access This is an Open Access article distributed in accordance with the terms of the Creative Commons Attribution (CC BY 4.0) license, which permits others to distribute, remix, adapt and build upon this work, for commercial use, provided the original work is properly cited. See: http://creativecommons.org/licenses/by/4.0/

REFERENCES

1 Zar HJ, Ferkol TW. The global burden of respiratory disease-impact on child health. *Pediatr Pulmonol* 2014;49:430–4.
2 Rudan I, O'Brien KL, Nair H, *et al*. Epidemiology and etiology of childhood pneumonia in 2010: estimates of incidence, severe morbidity, mortality, underlying risk factors and causative pathogens for 192 countries. *J Glob Health* 2013;3:010401.
3 Zar HJ, Madhi SA, Aston SJ, *et al*. Pneumonia in low and middle income countries—progress and challenges. *Thorax* 2013;68:1052–56.
4 Sears MR, Greene JM, Willan AR, *et al*. A longitudinal, population-based, cohort study of childhood asthma followed to adulthood. *N Engl J Med* 2003;349:1414–22.
5 Edmond K, Scott S, Korczak V, *et al*. Long term sequelae from childhood pneumonia; systematic review and meta-analysis. *PLoS One* 2012;7:e31239.

Confidential: For Review Only

Downloaded from http://thorax.bmj.com/ on March 5, 2018 - Published by group.bmj.com

THORAX

Investigating the early-life determinants of illness in Africa: the Drakenstein Child Health Study

H J Zar, W Barnett, L Myer, D J Stein and M P Nicol

*Thorax*2015 70: 592-594 originally published online September 16, 2014
doi: 10.1136/thoraxjnl-2014-206242

Updated information and services can be found at:
http://thorax.bmj.com/content/70/6/592

These include:

References This article cites 5 articles, 1 of which you can access for free at:
http://thorax.bmj.com/content/70/6/592#ref-list-1

Open Access This is an Open Access article distributed in accordance with the terms of the Creative Commons Attribution (CC BY 4.0) license, which permits others to distribute, remix, adapt and build upon this work, for commercial use, provided the original work is properly cited. See:
http://creativecommons.org/licenses/by/4.0/

Email alerting service Receive free email alerts when new articles cite this article. Sign up in the box at the top right corner of the online article.

Topic Collections Articles on similar topics can be found in the following collections

Open access (276)
Drugs: infectious diseases (968)
Epidemiologic studies (1829)
TB and other respiratory infections (1273)

Notes

To request permissions go to:
http://group.bmj.com/group/rights-licensing/permissions

To order reprints go to:
http://journals.bmj.com/cgi/reprintform

To subscribe to BMJ go to:
http://group.bmj.com/subscribe/

Confidential: For Review Only

BMJ Paediatrics Open

Confidential

Protocol: The Drakenstein Child Health Study (DCHS)- Investigating determinants of early child development and cognition

Journal:	*BMJ Paediatrics Open*
Manuscript ID	bmjpo-2018-000282.R1
Article Type:	Protocol
Date Submitted by the Author:	04-May-2018
Complete List of Authors:	Donald, Kirsten; University of Cape Town Department of Paediatrics and Child Health, Division of Developmental Paediatrics<br>Hoogenhout, Michelle; University of Cape Town Department of Paediatrics and Child Health, Division of Developmental Paediatrics<br>du Plooy, Christopher; University of Cape Town Department of Paediatrics and Child Health, Division of Developmental Paediatrics<br>Wedderburn, Catherine; University of Cape Town Department of Paediatrics and Child Health, Division of Developmental Paediatrics; London School of Hygiene and Tropical Medicine Department of Clinical Research<br>Nhapi, Raymond; South African Medical Research Council, Unit on Child & Adolescent Health<br>Barnett, Whitney; South African Medical Research Council, Unit on Child & Adolescent Health; University of Cape Town Department of Paediatrics and Child Health<br>Hoffman, Nadia; UCT Department of Psychiatry and Mental Health<br>Malcolm-Smith, Susan; University of cape town Department of Psychology, Applied Cognitive Science and Experimental Neuropsychology Team<br>Zar, Heather; South African Medical Research Council, Unit on Child & Adolescent Health; University of Cape Town Department of Paediatrics and Child Health<br>Stein, Dan; UCT Department of Psychiatry and Mental Health; South African Medical Research Council, Unit on Risk and Resilience in Mental Disorders
Keywords:	Neurodevelopment, Child Psychiatry

SCHOLARONE™
Manuscripts

Protocol: The Drakenstein Child Health Study (DCHS)-Investigating determinants of early child development and cognition

Author List: Kirsten A Donald[1], Michelle Hoogenhout[1], Christopher P du Plooy[1], Catherine J Wedderburn[1,2], Raymond T Nhapi[3], Whitney Barnett[3,4], Nadia Hoffman[5], Susan Malcolm-Smith[6], Heather J Zar[3,4], Dan J Stein[5,7].

Affiliation:

[1]Division of Developmental Paediatrics, Department of Paediatrics & Child Health, Red Cross War Memorial Children's Hospital, University of Cape Town, South Africa

[2]Department of Clinical Research, London School of Hygiene & Tropical Medicine, UK

[3]South African Medical Research Council (SAMRC), Unit on Child & Adolescent Health, Cape Town, South Africa

[4]Department of Paediatrics & Child Health, Red Cross War Memorial Children's Hospital, University of Cape Town, South Africa

[5]Department of Psychiatry & Mental Health, University of Cape Town, South Africa

[6]Applied Cognitive Science and Experimental Neuropsychology Team, Department of Psychology, University of Cape Town, South Africa

[7]South African Medical Research Council (SAMRC), Unit on Risk and Resilience in Mental Disorders, Cape Town, South Africa

Corresponding author:

Kirsten A Donald

Email: Kirsty.Donald@uct.ac.za

Phone: 021 6585322

Word count: 2325

Confidential: For Review Only

What is known about the subject?

There is growing awareness that psychosocial risk and resilience factors in early life play a key role in influencing later health.

Most work has been done in high income settings, rather than low- and middle-income countries (LMICs), where the majority of the global childhood population resides.

The few studies with well-defined cohorts in LMICs have employed various methods and measures, making comparisons across studies challenging.

What this study hopes to add

The Drakenstein Child Health Study (DCHS) aims to provide an understanding of the effects of multiple risk and mitigating factors on child health and development in a LMIC.

For Peer Review Only

Abstract

Introduction: There is growing awareness that psychosocial risk and resilience factors in early life play a key role in influencing later health. Most work has been done in high income settings, rather than low- and middle-income countries (LMICs), where the majority of the global childhood population resides. The few studies with well-defined cohorts in LMICs have employed various methods and measures, making comparisons across studies challenging. This presentation describes the methodology for infant and child developmental measures used in the Drakenstein Child Health Study (DCHS), a multi-disciplinary longitudinal birth cohort study in South Africa.

Methods and analysis. We outline a multilevel approach combining a range of measures including parental reports, behaviour observations, clinician-administered scales and brain imaging. Using this approach, we aim at a longitudinal perspective of developmental, cognitive, socioemotional and neurophysiological outcomes in a birth cohort of children in a LMIC.

Ethics and dissemination: The study was approved by the faculty of Health Sciences, Human Research Ethics Committee, University of Cape Town (401/2009), by Stellenbosch University (N12/02/0002), and the Western Cape Provincial Health Research committee (2011RP45).

Discussion: Children in the DCHS develop in a context typical of many communities in South Africa and other LMICs. There is a critical need for research in LMICs to elucidate underlying factors that inform risk for, and resilience to, poor developmental outcomes in infants born into high risk communities. Such work may inform effective intervention strategies appropriate to this context.

Keywords*:* global health, child development, assessment, cognition, socio-emotional development, resilience

Confidential: For Review Only

Introduction

Risk and resilience factors encountered during the early years of life have an enduring influence on later physiological and psychological outcomes [1-3]. A number of risk factors are already apparent in utero; for example, antepartum maternal psychological distress and depression can adversely affect infant physical, neurocognitive and socioemotional developmental outcomes [4-6]. During early childhood, exposure to stressors such as familial violence and abuse has been associated with increased risk of behaviour problems, autoimmune disorders, cardiovascular disease, and premature mortality [7-10]. In LMICs, key risk factors such as HIV infection and prenatal maternal malnutrition are responsible for millions of children failing to reach their full developmental potential [11-13]. Poor child outcomes may have intergenerational effects, so exacerbating their impact [12].

At the same time, protective factors may be associated with increased resilience, and so with positive mental health and developmental outcomes in the face of stressors [14]. Resilience is thought to arise from the interplay between factors at the individual, family and community levels [15]. Protective factors can be highly context-specific and can exert different effects at different timepoints [15 16]. Thus, longitudinal studies provide the best opportunities to identify protective factors at various stages of development, as well as sensitive periods for intervention. However, most studies on resilience have been done in high-income countries, where contextual factors may be different.

Indeed, the vast majority of previously reported studies have focused on psychobiological and psychosocial risk profiles in well-resourced countries. These profiles differ considerably in LMICs. For instance, there is, in general, considerably higher prevalence of low birth weight, childhood malnutrition and infectious diseases in LMICs [17 18]. In addition, critical psychosocial factors that are known to have impact on child development

Confidential: For Review Only

such as maternal depression and exposure to violence frequently have a greater prevalence in these high risk communities [12 13 19]. There is considerable work in LMICs, including work that is longitudinal and that is culturally appropriate. However, various methods and measures haven been used, so that cross-cohort comparison is not always possible.

The Drakenstein Child Health Study (DCHS) is a multi-disciplinary longitudinal study investigating the early life determinants of child health in two peri-urban communities in the Western Cape Province, South Africa [20]. The early-life component focuses on a broad spectrum of child health outcomes, including physical health and growth as well as neurodevelopmental, cognitive and psychological health. The study investigates the role and interaction of environmental, infectious, psychosocial, nutritional, genetic, maternal and immunological risk and protective factors for development. The DCHS follows an extensively phenotyped cohort over critical early years, aiming to provide an understanding into the effects of multiple risk and mitigating factors, and their interactions, on child health and development in a LMIC. This paper describes the methodology for the infant and child measures of the psychosocial component of the DCHS. By documenting the measures used and our reasons for choosing these, we hope to improve future harmonisation between cohort studies.

Confidential: For Review Only

Methods and analysis

Design and Setting

The DCHS is located in the peri-urban Drakenstein district, Western Cape, South Africa. The communities surrounding the Mbekweni and TC Newman clinics are ethnically, culturally and linguistically heterogeneous. Mbekweni consists of a mostly isiXhosa-speaking black African population, whereas TC Newman consists of a mostly Afrikaans-speaking mixed

race population [20]. However, both communities are characterised by low socio-economic status and feature a high prevalence of multiple psychosocial risk factors; including single parent households, high rates of psychological distress, and exposure to violence, HIV and substance abuse[21]. In particular, risk factors that may be highly prevalent in these communities and similar communities in the region include high rates of HIV exposure [20 22 23], high prevalence of maternal psychological distress and depression [5 21 24-26], high rates of drug and alcohol usage [21 24 27 28], high levels of violence and intimate partner violence [29], and low levels of employment and educational attainment [21]. The population is stable, with little immigration or emigration. More than 90% of people in the district use the public health system. In this regard, the communities are representative of many other communities in South Africa and other low and middle-income countries (LMICs).

Participants

Pregnant women were recruited while attending routine antenatal care at Mbekweni or TC Newman clinic between March 2012 and March 2015. Women were enrolled in the DCHS at 20 to 28 weeks' gestation and were followed through birth and postnatally. Pregnant mothers were eligible for the study if they were 18 years or older, planned to attend antenatal care at one of the two clinics and intended to remain in the area for at least a year. Expecting mothers provided informed written consent at enrolment and were re-consented annually following childbirth.

Mothers were provided informed consent in their preferred language, English, Afrikaans or isiXhosa, by trained study staff from the community. Informed consent forms described the scope and aims of the study, including potential harm or benefits. In total, 1137 mother-child dyads were enrolled in the study; of which four mothers had twins and one had

Confidential: For Review Only

triplets. Thus, 1143 children were enrolled in the study. A conservative cumulative attrition of 20% over five years was estimated, and the sample size was calculated accordingly. Enrolment criteria was broad to ensure generalizability and that the cohort would be representative of the general population. Majority of Drakenstein sub-district births occur at Paarl Hospital, with an average of 4800 births per year, the study enrolled approximately 10% of catchment area births. Of pregnant women in the catchment area, who were provided information relating to study enrolment, 1471 mothers were determined to be ineligible (based on age, gestation or non-attendance at study clinics). A further 674 mothers were eligible but were not interested in study enrolment. Where data available for the Cape Winelands district, the study population has comparable levels of education, partnership status and household size. Based on these sociodemographic variables and the broad enrolment criteria used, we believe that the study population is representative of the source population.

Measures

Mothers were followed during pregnancy and childbirth. Following birth, infants and mothers returned to the clinics and were asked to complete self-report and clinician-administered measures at time points ranging from 2-4 weeks to 5.5 years (see Figure 1). At the time of submission, the oldest children in the cohort are 5 years old and the youngest children are currently 2 years old.

[Fig. 1 here]

The infant and child developmental and psychosocial measures are described here. The overview methods of the larger DCHS are described elsewhere [20], as is the maternal and paternal psychosocial evaluation component of the study [21]. Broadly, the child measures

For Review Only

assessed (1) social and biological risk and protective factors, (2) general neurobiological development, (3) cognitive development, and (4) socio-emotional development. These measures were chosen for their ability to identify multivariate risk and protective factors for child health outcomes. Measures were translated from English to isiXhosa and Afrikaans using the standard forward and backwards-translation method [30]. Assessments were conducted at community centres located near the two clinics.

The tests used are detailed in Table 1 with detailed description in supplementary appendix. The measures of social and biological risk and protective factors (e.g. exposure to violence, alcohol and tobacco use, parenting practices, and attachment) were completed by the mothers during several antenatal and postnatal study visits. Additionally, dyadic interactions between the mother and child were recorded. Basic demographic data and health information for the infants were obtained from participants' hospital records. The child's cognitive and general development was assessed directly at several different time points. Early child development was assessed by trained physio and occupational therapists at the clinics supervised by a paediatric neurodevelopment specialist. Cognitive assessments which include language, fine motor, executive functioning, memory and general and social cognitive ability, were administered by trained research assistants, with the aid of trained interpreters when necessary. Child socio-emotional development was captured through a combination of observational, self-report and parent-report measures (see supplementary materials). The socio-emotional assessment included measures of emotion regulation, affective arousal and social attention-allocation, empathy, morality, prosocial behaviour, temperament and callous-unemotional traits. Additionally, a subgroup of children in the Drakenstein Child Health Study cohort underwent multimodal neuroimaging assessment at the Cape Universities Brain Imaging Centre (CUBIC). The imaging modalities performed included: (1) structural magnetic resonance imaging (MRI) with T1 and T2-weighting to

Confidential: For Review Only

examine cortical and subcortical volumes; (2) diffusion tensor imaging (DTI) for white matter microstructure; (3) magnetic resonance spectroscopy (MRS) and; (4) resting state functional MRI for regional connectivity.

[Table 1 here]

Descriptive statistics

We use descriptive statistics (medians, interquartile ranges, counts & percentages) to present the sociodemographic variables of the cohort. These statistics are presented on the two study sites (Mbekweni and TC Newman) as well as the overall cohort. Chi-Square and Mann-Whitney tests were used to test for differences between the sites. As the study is still ongoing, the data presented here was collected antenatally and at birth.

Child sociodemographic characteristics

The cohort followed 1137 mothers over the course of three years during the initial recruitment period. During this time there was a total of 1143 live births including 4 sets of twins and 1 set of triplets (see Table 2). Within this group, 17.2% of children were born preterm (defined here as less than 37 weeks' gestation). The birth weight and lengths of the children have been converted to z-scores according to WHO standardisation and adjusted for gestation. In line with our previous reports [31 32], the children at TC Newman clinic were born significantly smaller in weight compared to Mbekweni children; however, the IQR falls within two z-scores of 0 in both clinics.

[Table 2 here]

Confidential: For Review Only

Maternal and family sociodemographic characteristics

The maternal and family characteristics are presented in Table 3. Socioeconomic status was low across both clinics as shown by the low household income levels, maternal employment rates of 27% and maternal educational attainment. The median maternal age at enrolment was 26 years (IQR 22–31) and the majority of mothers had completed some secondary schooling by this point. The mothers reported that over 65% of pregnancies were unplanned. Approximately 40% were currently married or cohabiting with their partner, and a high proportion reported that partners were supportive, although this differed across clinics.

[Table 3 here]

Mothers and children in both communities were frequently exposed to community violence (see Table 4). Levels of violence exposure were higher in TC Newman than in Mbekweni, but both communities from this cohort reported greater exposure to violence than reported in a previous study in South Africa [33]. Exposure to violence is thus highly prevalent in these communities both within the home and external community environment. Both communities reported less consistent parenting (i.e. higher consistency scores), but otherwise similar parenting styles and family adjustment, compared to European samples [34]. Mothers in Mbekweni reported more coercive parenting, less consistency and less encouragement, but also better family relationships and parental teamwork than in TC Newman.

[Table 4 here]

Confidential: For Review Only

Confidential: For Review Only

Ethics and dissemination

The study was approved by the faculty of Health Sciences, Human Research Ethics Committee, University of Cape Town (401/2009), by Stellenbosch University (N12/02/0002), and the Western Cape Provincial Health Research committee (2011RP45).

Confidential: For Review Only

Discussion

This paper highlights the rationale and approach to assessing both psychosocial risk and protective factors impacting the development of children in a high-risk South African communities. The study follows a multilevel approach that targets developmental, cognitive, socioemotional and physiological outcomes. . As can be seen from some of our baseline data (n = 1143), there are a broad number of sociodemographic risk and resilience factors for children in this region. Demographic and sociodemographic data show that although these communities are in close proximity, they differ substantively in social and financial resources.

Given these sorts of risk and resilience factors, it is important to assess child outcomes using a multidimensional approach [35]. This includes three important components which are built into this cohort. Firstly, the DCHS collects biomedical and psychosocial risk factors across a wide range of factors in the prenatal period and first years of life. These include both factors which are known to put children at risk for poor outcome such as maternal mental health, substance use disorders, poor nutrition and factors known to be protective or hypothesised to potentially support development including early infant feeding choices, pregnancy support and maternal bonding and attachment styles. Secondly, the outcome measures described in this manuscript are also multidimensional and allow examination of outcomes in terms of the dyadic relationship and the family system into which these children are born. Thirdly, the cohort is following these mother infant pairs over time. Longitudinal data (with repeat measures) allows the investigation of developmental, cognitive and socio-emotional trajectories as well as the interactions between exposures within the context of this cohort. The investigation of the timing of maximum windows of vulnerability becomes possible with this approach.

Confidential: For Review Only

Attention to the ethical issues requires consideration in a study of this type. The DCHS maintains an active programme focused on community engagement, including regular engagement with study participants for feedback on study involvement, active dissemination of research results to key local stakeholders and distribution of health promotion information to study participants. Given the context, a key ethical obligation includes screening within the study population for physical and mental health issues, in both mothers and children. Screening is done in the DCHS in conjunction with an active referral system and is bolstered by close relationships between study staff and provincial health staff. All women involved in the study, independent of identified mental or physical health problems, receive information regarding service providers in the area. The network of investigators in the DCHS with strong and relevant clinical background in the South African public health environment is a strength of the research and has allowed realistic and integrated referral systems to be developed and implemented as part of the study.

Very few cohorts are reported which take into account the composite effects of multiple factors on health and development in the early years. This is especially true of cohorts from LMICs where young children are exposed to overlapping epidemics of infectious and non-communicable diseases. The P-MaMie birth cohort in Ethiopia[36] collected information on maternal mental health as well as growth and developmental outcomes in very young infants, but represent an almost entirely rural community in Africa with attendant risk factors likely to vary from a peri-urban community described in the DCHS. With urbanisation representing a critical current epidemiological phenomenon, documenting communities in this context becomes increasingly important. The Pelotas cohorts in Brazil[37] represent one of the longest standing set of population based cohorts in the global South. These are large cohorts capturing whole communities have been documenting maternal and child outcomes for over 20 years. The most recent of these cohorts starting in 2015 is the first of these to collect

Confidential: For Review Only

prospective antenatal data on the mothers. Though smaller, the DCHS, at present has been able to collect the most comprehensive set of biological, psychosocial, environmental, maternal and child data and so carefully measures outcomes through use of sensitive modalities including brain imaging in a high-risk setting. Sensitivity to exposures, individually and together, in both external and internal environments are different at specific ages and periods in development. The developmental window spanning the 40 weeks of pregnancy and the first years of life appear to be a critical period where environmental exposure may cause embedded effects which may have impact across the lifespan. There is a critical need for research in this field to elucidate the underlying factors that inform risk for and resilience from poor developmental outcomes in infants born into high risk communities which may ultimately inform effective preventative and ameliorating intervention strategies appropriate to this context. From a public health perspective, a better understanding of the relevant mechanisms is critical, as this may ultimately drive preventative and targeted therapeutic approaches.

The United Nation's Sustainable Development Goals (SDGs) were officially adopted in 2015. These cross cutting goals consistently forefront the importance of addressing programmes for targeting maternal and child health, and in particular the theme of early child growth and development, as being key in the global strategy to optimize human health and well-being. In the South African setting, where children make up around a third of the population expectant mothers and their young infants are a particularly important focus. Much more attention is needed to address maternal and infant health, in order to decrease early mortality and later morbidity in this vulnerable population.

Limitations

Confidential: For Review Only

Limitations of the study include the fact that though extremely well characterised, the cohort is a modest size. Though measures investigating aspects of child health, development and cognition have been administered to as much of the cohort as possible, certain measures have been administered only on a subset of the group (e.g., neuroimaging) due to participant burden and the cost of assessment. Although care was taken to translate measures into Afrikaans and isiXhosa, there will always be some difficulty in interpreting the results of measures designed in a different language and cultural context. Every effort was made to use tools which minimised problems in this area.

Confidential: For Review Only

Data sharing statement

Collaborations for the analysis of data are welcome. The DCHS has a large and active group of investigators and postgraduate students and many have successfully partnered with researchers from other institutions. In particular, we encourage collaborations that lead to skills transfer and capacity building for postgraduate students. Researchers who are interested in datasets or collaborations can contact the PI, Heather Zar [heather.zar@uct.ac.za] with a concept note outlining the request. More information can be found on our website [http://www.paediatrics.uct.ac.za/scah/dclhs].

Confidential: For Review Only

References

1. Miller GE, Chen E, Parker KJ. Psychological stress in childhood and susceptibility to the chronic diseases of aging: moving toward a model of behavioral and biological mechanisms. *Psychol Bull* 2011;137(6):959.
2. Gluckman PD, Hanson MA, Cooper C, et al. Effect of in utero and early-life conditions on adult health and disease. *N Engl J Med* 2008;359(1):61-73. doi: 10.1056/NEJMra0708473 [published Online First: 2008/07/04]
3. Heim C, Shugart M, Craighead WE, et al. Neurobiological and psychiatric consequences of child abuse and neglect. *Dev Psychobiol* 2010;52(7):671-90. doi: 10.1002/dev.20494
4. Kingston D, Tough S, Whitfield H. Prenatal and postpartum maternal psychological distress and infant development: a systematic review. *Child Psychiatry Hum Dev* 2012;43(5):683-714.
5. Gelaye B, Rondon M, Araya R, et al. Epidemiology of maternal depression, risk factors, and child outcomes in low-income and middle-income countries. *Lancet Psychiatry* 2016;3(10):973-82. doi: 10.1016/S2215-0366(16)30284-X
6. Davalos DB, Yadon CA, Tregellas HC. Untreated prenatal maternal depression and the potential risks to offspring: a review. *Arch Womens Ment Health* 2012;15(1):1-14.
7. Dong M, Giles WH, Felitti VJ, et al. Insights into causal pathways for ischemic heart disease adverse childhood experiences study. *Circulation* 2004;110(13):1761-66.
8. Dube SR, Fairweather D, Pearson WS, et al. Cumulative childhood stress and autoimmune diseases in adults. *Psychosom Med* 2009;71(2):243.
9. Anda RF, Dong M, Brown DW, et al. The relationship of adverse childhood experiences to a history of premature death of family members. *BMC Public Health* 2009;9(1):1.
10. Chander P, Kvalsvig J, Mellins CA, et al. Intimate Partner Violence and Child Behavioral Problems in South Africa. *Pediatrics* 2017 doi: 10.1542/peds.2016-1059
11. Grantham-McGregor S, Cheung YB, Cueto S, et al. Developmental potential in the first 5 years for children in developing countries. *The Lancet* 2007;369:60-70. doi: 10.1016/S0140-6736(07)60032-4
12. Walker SP, Wachs TD, Meeks Gardner J, et al. Child development: risk factors for adverse outcomes in developing countries. *The Lancet* 2007;369:145-57. doi: 10.1016/S0140-6736(07)60076-2
13. Walker SP, Wachs TD, Grantham-McGregor S, et al. Inequality in early childhood: risk and protective factors for early child development. *The Lancet* 2011;378(9799):1325-38.
14. Luthar SS, Cicchetti D, Becker B. The construct of resilience: A critical evaluation and guidelines for future work. *Child Dev* 2000;71:543-62. doi: 10.1111/1467-8624.00164
15. Ungar M, Ghazinour M, Richter J. Annual Research Review: What is resilience within the social ecology of human development? *J Child Psychol Psychiatry* 2013;54:348-66. doi: 10.1111/jcpp.12025
16. Masten AS. Global perspectives on resilience in children and youth. *Child Dev* 2014;85:6-20. doi: 10.1111/cdev.12205
17. Black RE, Victora CG, Walker SP, et al. Maternal and child undernutrition and overweight in low-income and middle-income countries. *The Lancet* 2013;382:427-51. doi: 10.1016/S0140-6736(13)60937-X
18. Remais JV, Zeng G, Li G, et al. Convergence of non-communicable and infectious diseases in low- and middle-income countries. *Int J Epidemiol*:221-27. doi: 10.1093/ije/dys135
19. Rahman A, Iqbal Z, Harrington R. Life events, social support and depression in childbirth: perspectives from a rural community in the developing world. *Psychol Med* 2003;33 doi: 10.1017/s0033291703008286
20. Zar HJ, Barnett W, Myer L, et al. Investigating the early-life determinants of illness in Africa: the Drakenstein Child Health Study. *Thorax* 2014:thoraxjnl-2014-206242.

Confidential: For Review Only

21. Stein DJ, Koen N, Donald KA, et al. Investigating the psychosocial determinants of child health in Africa: the Drakenstein Child Health Study. *J Neuroscience methods* 2015;70(6):592-94.
22. Zar HJ, Barnett W, Stadler A, et al. Aetiology of childhood pneumonia in a well vaccinated South African birth cohort: a nested case-control study of the Drakenstein Child Health Study. *The Lancet Respiratory Medicine* 2016;4(6):463-72.
23. Shisana O, Rehle T, Simbayi LC, et al. South African national HIV prevalence, incidence and behaviour survey, 2012. 2014
24. Dewing S, Tomlinson M, le Roux IM, et al. Food insecurity and its association with co-occurring postnatal depression, hazardous drinking, and suicidality among women in peri-urban South Africa. *J Affect Disord* 2013;150(2):460-5. doi: 10.1016/j.jad.2013.04.040 [published Online First: 2013/05/28]
25. Hartley M, Tomlinson M, Greco E, et al. Depressed mood in pregnancy: prevalence and correlates in two Cape Town peri-urban settlements. *Reproductive health* 2011;8(1):9.
26. Roos A, Faure S, Lochner C, et al. Predictors of distress and anxiety during pregnancy. *African journal of psychiatry* 2013;16(2):118-22.
27. Petersen Williams P, Jordaan E, Mathews C, et al. Alcohol and other drug use during pregnancy among women attending midwife obstetric units in the Cape Metropole, South Africa. *Advances in preventive medicine* 2014;2014
28. Vythilingum B, Roos A, Faure SC, et al. Risk factors for substance use in pregnant women in South Africa. *SAMJ: South African Medical Journal* 2012;102(11):853-54.
29. Groves AK, Moodley D, McNaughton-Reyes L, et al. Prevalence, rates and correlates of intimate partner violence among South African women during pregnancy and the postpartum period. *Matern Child Health J* 2015;19(3):487-95. doi: 10.1007/s10995-014-1528-6 [published Online First: 2014/06/04]
30. Smit J, Van den Berg C, Bekker L, et al. Translation and cross-cultural adaptation of a mental health battery in an African setting. *African Health Sciences* 2006;6(4)
31. Brittain K, Myer L, Koen N, et al. Risk Factors for Antenatal Depression and Associations with Infant Birth Outcomes: Results From a South African Birth Cohort Study. *Paediatric and perinatal epidemiology* 2015;29(6):505-14.
32. Vanker A, Barnett W, Brittain K, et al. Antenatal and early life tobacco smoke exposure in an African birth cohort study. *The International Journal of Tuberculosis and Lung Disease* 2016;20(6):729-37.
33. Martin L, Revington N, Seedat S. The 39-item Child Exposure to Community Violence (CECV) Scale: Exploratory factor analysis and relationship to PTSD symptomatology in trauma-exposed children and adolescents. *International journal of behavioral medicine* 2013;20(4):599-608.
34. Mejia A, Filus A, Calam R, et al. Measuring parenting practices and family functioning with brief and simple instruments: validation of the Spanish version of the PAFAS. *Child Psychiatry & Human Development* 2015;46(3):426-37.
35. Robinson O, Basagaña X, Agier L, et al. The pregnancy exposome: Multiple environmental exposures in the INMA-Sabadell birth cohort. *Environ Sci Technol* 2015;49:10632-41. doi: 10.1021/acs.est.5b01782
36. Servili C, Medhin G, Hanlon C, et al, Maternal common mental disorders and infant development in Ethiopia: the P-MaMiE Birth Cohort. *BMC Public Health* 2010, 10:693. http://www.biomedcentral.com/1471-2458/10/693
37. Hallal P, Bertoldi A, Domingues M et al. Cohort Profile: The 2015 Pelotas (Brazil) Birth Cohort Study. International Journal of Epidemiology, dyx219, https://doi.org/10.1093/ije/dyx219

Confidential: For Review Only

Confidential: For Review Only

Authors' contributions

This study requires multidisciplinary expertise in the execution of measures of this type. DJS is PI of the psychosocial aspects of the DCHS cohort and contributed to the design and decision making involving psychosocial tools and measures used as well as general study design. HJZ is PI of the umbrella DCHS cohort and played a central role in the operational aspects and design of the study. KAD, PI of the child psychosocial aspects of the study, was involved in the design of the study, operational aspects of the study, and played a key role in the child psychosocial measures used. SMS, MH, and CP contributed to decision making involving tools used. NH, WB, and CJW contributed to the operational aspects of the study, QC of data described and data management. RTN contributed to data management. Authors contributed to sections relating to their area of expertise in the manuscript. All authors reviewed and approved the final version of this manuscript.

Confidential: For Review Only

Funding statement

This work was supported by the Bill and Melinda Gates Foundation [OPP 1017641]. Additional support for HJZ and DJS by the MRC of South Africa. Additional aspects of the work reported here are supported by the South African NRF and MRC, by an Academy of Medical Sciences Newton Advanced Fellowship (NAF002\1001), funded by the UK Government's Newton Fund, by NIAAA via (R21AA023887) and by the Collaborative Initiative on Fetal Alcohol Spectrum Disorders (CIFASD) developmental grant (U24 AA014811), by the US Brain and Behaviour Foundation Independent Investigator grant (24467). WB is supported by the SAMRC National Health Scholars programme, and CJW is supported by the Wellcome Trust through a Research Training Fellowship [203525/Z/16/Z].

Confidential: For Review Only

Competing interests statement

The authors declare no competing interests.

Confidential: For Review Only

Acknowledgements

We thank the study staff in Paarl, the study data team and lab teams, the clinical and administrative staff of the Western Cape Government Health Department at Paarl Hospital and at the clinics for support of the study. We acknowledge the advice from members of the study International Advisory Board and thank our collaborators. We thank the families and children who participated in this study.

Confidential: For Review Only

Table 1 Child psychosocial and neurodevelopmental measures

Domain	Measure
**Demographic data**	Household income and assets, maternal education, employment
**Infant health information**	Weight, height, diagnoses
**Infant neurodevelopment**	MRI, DTI, MRS, fMRI
**Antenatal risk factors**	Birth planning; partner support; maternal depression; alcohol, nicotine and illicit substance use; maternal childhood trauma; intimate partner violence; exposure to stressful events; psychological distress; symptoms of peri- and posttraumatic stress
**Trauma and exposure**	
Emotional distress	Paediatric emotional distress scale
Exposure to violence	Child exposure to community violence
	Survey for exposure to community violence
**Parenting**	
Dyadic interaction[a]	Global rating scale
	Emotional availability scale
Parenting practices	Parenting and family adjustment scale
Attachment	Brockington postpartum bonding questionnaire
**Resilience**	Child and youth resilience measure
**General development**	Western Cape screen
	Bayley Scales of Infant and Toddler Development, Third Edition (Bayley-III)
**General cognitive function**	Kaufman assessment battery for children (KABC-II)
Problem solving	KABC-II Conceptual thinking
Visual-spatial memory/processing	KABC-II Face recognition
Visual-spatial processing and problem solving	KABC-II Triangles
Working memory and motor sequencing	KABC-II Hand movements
**Language**	Peabody picture vocabulary test, fourth edition (PPVT-IV)
	KABC-II Expressive vocabulary
**Memory**	
Learning	KABC-II Atlantis
Delayed recall	KABC-II Atlantis delayed
**Executive function**	
Working memory	Wechsler preschool and primary scale of intelligence, fourth edition (WPPSI-IV): Picture memory
Inhibition	Day-night task
Cognitive flexibility	Dimension change card sort
Attention	Test of everyday attention (TEA-Ch): Sky search
**Motor control**	Bayley-III: Fine motor
	Grooved peg board

Confidential: For Review Only

**Social cognition**	
Theory of mind	Diverse desires
	Diverse beliefs
	Understanding intentions
	Perception-leads-to-knowledge
	Location-change false belief
	Unexpected contents false belief
	Belief emotion
	Hidden emotions
Emotion recognition	NEPSY-II: Affect recognition
**Effortful control / emotion regulation**	Snack delay
	Gift-in-bag
	Whisper
	Rydell's emotion questionnaire
**Social competence**	
Affective arousal	Pupil dilation
Attention allocation	Gaze fixation
Empathy	Chicago empathy for pain task
	Questionnaire of cognitive and affective empathy
Prosocial behaviour	Dictator game
Internalising and externalising behaviour	Child behavior checklist
**Temperament/ Personality**	
Temperament	Rothbart infant and early child behavior questionnaires[a]
Callous-unemotional traits	Callous unemotional screening device

Note. References for the measures are given in the appendix. MRI: Magnetic Resonance Imaging, DTI: Diffusion Tensor Imaging, MRS: Magnetic Resonance Spectroscopy, fMRI: functional Magnetic Resonance Imaging
[a]very short forms

Confidential: For Review Only

Table 2 Child sociodemographic characteristics

**Variable**	**Mbekweni**	**TC Newman**	**Total**	***p*-value**
	Number (% of each clinic)			
Mothers	628	509	1137	
Live births	634	509	1143	
Twin sets	4	0	4	
Triplet sets	1	0	1	
Preterm births	107 (16.88)	83 (16.31)	190 (16.32)	0.797
Child's Race				
Black	627 (99.05)	6 (0.95)	633 (55.38)	
Mixed	7 (1.11)	503 (98.82)	510 (44.62)	< 0.001
	Median (IQR)			
Birth weight z-score [a]	-0.4 (-1.2 – 0.2)	-0.7 (-1.4 – -0.1)	-0.5 (-1.3 – 0.1)	< 0.001
Birth length z-score [a]	0.1 (-0.9 – 1.0)	-0.03 (-0.9 – 0.8)	0.003 (-0.9 – 0.9)	0.151

[a] Adjusted for gestation

Confidential: For Review Only

Table 3 Maternal and family sociodemographic characteristics

**Variable**	**Cape Winelands***	**Mbekweni**	**TC Newman**	**Total**	***p*-value**
	n=787,490	Number (% of each clinic)			
Maternal educational attainment					0.038
Primary		49 (7.8)	37 (7.3)	86 (7.6)	
Some secondary		340(54.1)	266 (52.3)	606 (53.3)	
Completed secondary	193,723 (24.6)	189 (30.1)	183 (36.0)	372 (32.7)	
Any tertiary	82,686 (10.5)	50 (8.0)	23 (4.5)	73 (6.4)	
Currently employed	675,666 (85.8)	157 (25.0)	149 (29.3)	306 (27.0)	0.106
Married / Cohabiting	282,709 (35.9)	237 (37.8)	221 (43.4)	458 (40.3)	0.054
Unplanned pregnancy		366 (68.0)	286 (62.7)	652 (65.6)	0.079
Partner support					< 0.001
Not at all / slightly supportive		42 (7.8)	46 (10.13)	88 (8.9)	
Moderately supportive		86 (16.0)	19 (4.2)	105 (10.6)	
Considerably / extremely supportive		409 (76.1)	389 (85.7)	798 (80.5)	
Reliance on partner for help					< 0.001
Not at all / not very often		53 (9.9)	46 (10.1)	99 (10.0)	
Some of the time		170 (31.7)	28 (6.2)	198 (20.0)	
Most / all of the time		314 (58.5)	380 (83.7)	694 (70.0)	
Monthly income	119,536/yr (avg)				< 0.001
< R1000 (< $75)		263 (41.9)	167 (32.8)	430 (37.8)	
R1000-R5000 ($75 - $374)		299 (47.6)	254 (49.9)	553 (48.6)	
> R5000 (> $374)		66 (10.5)	88 (17.3)	152 (13.5)	
		Median (IQR)			
Household size	3.7 persons	4 (3-6)	5 (4-7)	4 (3-6)	< 0.001
Mother's age at enrolment		27 (22 –32)	25 (21 –29)	26 (22 –31)	< 0.001

Census 2011 Municipal Report Western Cape. Statistics South Africa. Report No, 03-01-49. www.statssa.gov.za.

Confidential: For Review Only

Table 4 Family risk and protective factors

Variable	Mbekweni	TC Newman	Total	*p*-value
	Mean (SD)			
PAFAS Parenting				
Consistency	7.95 (2.01)	6.24 (2.92)	7.17 (2.61)	< 0.001
Coercive parenting	6.24 (3.38)	5.19 (3.98)	5.76 (3.70)	< 0.001
Encouragement	1.23 (1.66)	1.00 (1.50)	1.13 (1.59)	0.113
Parent-child relationship	0.63 (1.48)	1.37 (1.76)	0.97 (1.65)	< 0.001
PAFAS Family Adjustment				
Parental adjustment	2.72 (2.67)	2.36 (2.86)	2.56 (2.76)	0.025
Family relationships	1.46 (2.01)	2.45 (2.85)	1.91 (2.48)	< 0.001
Parental teamwork	1.32 (1.67)	1.93 (2.08)	1.59 (1.89)	< 0.001
SECV Total	21.27 (6.57)	26.92 (7.46)	23.84 (7.53)	< 0.001
CECV Total	38.65 (3.88)	40.13 (4.21)	38.65 (3.88)	< 0.001

Note. The questionnaires were administered at 2.5 years. Higher scores on the PAFAS indicate higher levels of dysfunction, i.e. higher consistency scores indicate less consistent parenting and higher coercive parenting scores indicate more coercive parenting.
PAFAS: Parenting and Family Adjustment Scale, SECV: Survey for Exposure to Community Violence, CECV: Child Exposure to Community Violence Checklist

Confidential: For Review Only

Figure captions

Figure 1 Time line for child assessment

Confidential: For Review Only

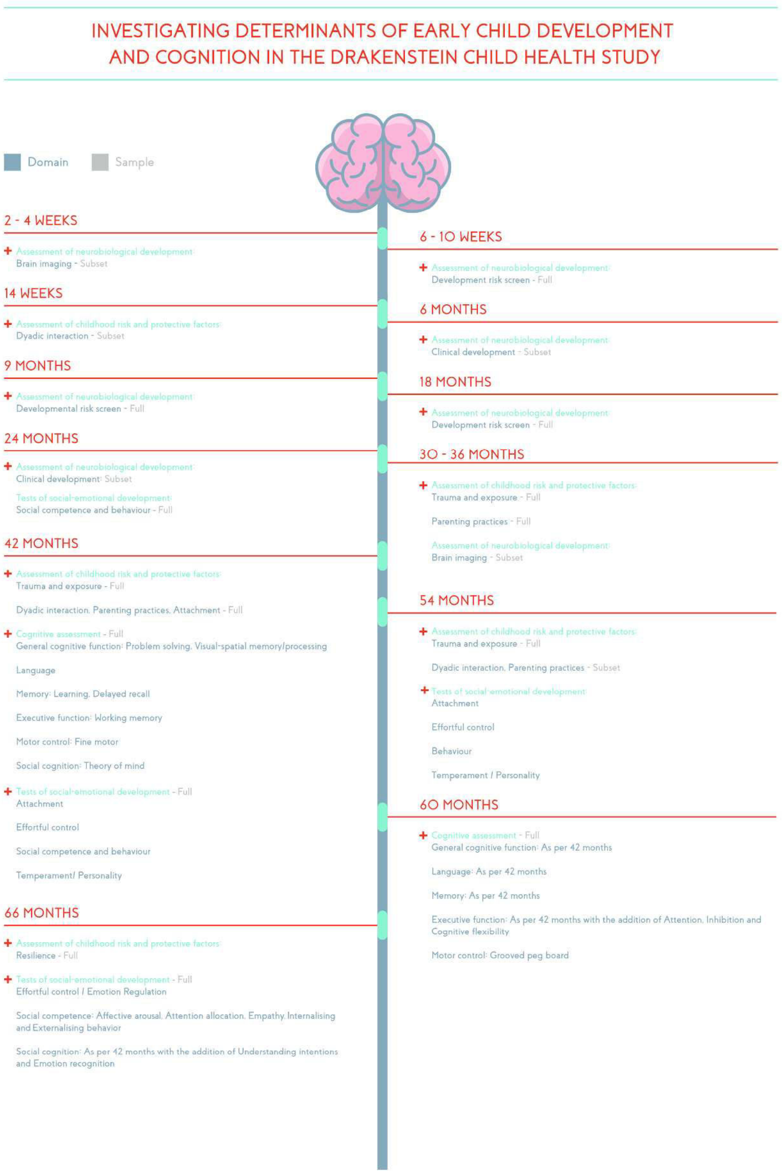

209x296mm (300 x 300 DPI)
